# The *Cymbidium* genome reveals the evolution of unique morphological traits

**DOI:** 10.1038/s41438-021-00683-z

**Published:** 2021-12-01

**Authors:** Ye Ai, Zhen Li, Wei-Hong Sun, Juan Chen, Diyang Zhang, Liang Ma, Qing-Hua Zhang, Ming-Kun Chen, Qing-Dong Zheng, Jiang-Feng Liu, Yu-Ting Jiang, Bai-Jun Li, Xuedie Liu, Xin-Yu Xu, Xia Yu, Yu Zheng, Xing-Yu Liao, Zhuang Zhou, Jie-Yu Wang, Zhi-Wen Wang, Tai-Xiang Xie, Shan-Hu Ma, Jie Zhou, Yu-Jie Ke, Yu-Zhen Zhou, Hsiang-Chia Lu, Ke-Wei Liu, Feng-Xi Yang, Gen-Fa Zhu, Laiqiang Huang, Dong-Hui Peng, Shi-Pin Chen, Siren Lan, Yves Van de Peer, Zhong-Jian Liu

**Affiliations:** 1 Key Laboratory of National Forestry and Grassland Administration for Orchid Conservation and Utilization at College of Landscape Architecture, Fujian Agriculture and Forestry University, Fuzhou, China; 2 Department of Plant Biotechnology and Bioinformatics, Ghent University, Gent, Belgium; 3 VIB Center for Plant Systems Biology, Gent, Belgium; 4 College of Forestry, Fujian Agriculture and Forestry University, Fuzhou, China; 5 Management Office of Yushan Scenic Area, Fuzhou, China; 6 College of Agriculture and Biotechnology, Zhejiang University, Hangzhou, China; 7 Key Laboratory of Plant Resource Conservation and Sustainable Utilization, South China Botanical Garden, Chinese Academy of Sciences, Guangzhou, China; 8 PubBio-Tech, Wuhan, China; 9 Tsinghua-Berkeley Shenzhen Institute (TBSI), Center for Biotechnology and Biomedicine and Shenzhen Key Laboratory of Gene and Antibody Therapy, State Key Laboratory of Chemical Oncogenomics, State Key Laboratory of Health Sciences and Technology, Institute of Biopharmaceutical and Health Engineering (iBHE), Shenzhen International Graduate School, Tsinghua University, Shenzhen, China; 10 Guangdong Key Laboratory of Ornamental Plant Germplasm Innovation and Utilization, Environmental Horticulture Research Institute, Guangdong Academy of Agricultural Sciences, Guangzhou, China; 11 Department of Biochemistry, Genetics and Microbiology, University of Pretoria, Pretoria, South Africa; 12 College of Horticulture, Academy for Advanced Interdisciplinary Studies, Nanjing Agricultural University, Nanjing, China; 13 Henry Fok College of Biology and Agriculture, Shaoguan University, Shaoguan, China; 14 Institute of Vegetable and Flowers, Shandong Academy of Agricultural Sciences, Jinan, China

**Keywords:** Genome duplication, Genome

## Abstract

The marvelously diverse Orchidaceae constitutes the largest family of angiosperms. The genus *Cymbidium* in Orchidaceae is well known for its unique vegetation, floral morphology, and flower scent traits. Here, a chromosome-scale assembly of the genome of *Cymbidium ensifolium* (Jianlan) is presented. Comparative genomic analysis showed that *C. ensifolium* has experienced two whole-genome duplication (WGD) events, the most recent of which was shared by all orchids, while the older event was the τ event shared by most monocots. The results of MADS-box genes analysis provided support for establishing a unique gene model of orchid flower development regulation, and flower shape mutations in *C. ensifolium* were shown to be associated with the abnormal expression of MADS-box genes. The most abundant floral scent components identified included methyl jasmonate, acacia alcohol and linalool, and the genes involved in the floral scent component network of *C. ensifolium* were determined. Furthermore, the decreased expression of photosynthesis-antennae and photosynthesis metabolic pathway genes in leaves was shown to result in colorful striped leaves, while the increased expression of MADS-box genes in leaves led to perianth-like leaves. Our results provide fundamental insights into orchid evolution and diversification.

## Introduction

With more than 25,000 species, Orchidaceae is the largest angiosperm family^1^, representing a staggering 8–10% of flowering plants. Orchids are renowned for their specialized flowers, showing extremely high diversity of epiphytic and terrestrial growth forms, and they are successful colonizers of a wide variety of different habitats^2^. Orchids share a similar morphology; their flowers are composed of three sepals, three petals (one modified to form a lip), and a column consisting of fused stamens and pistils^3^. Many species have floral scents to attract pollinators^4,5^. The evolution and molecular mechanisms of these and other traits of orchids are poorly understood, although the recent sequencing of several orchid genomes has started to shed light on their evolution and unique morphology and lifestyle^2,6–8^.

*Cymbidium* is a renowned genus of the orchid family that consists of 68 species, mainly distributed in tropical and subtropical areas of Asia, although some have also expanded into Papua New Guinea and Australia^9^. *Cymbidium* spp. exhibit various floral morphologies and unique floral scents and vegetation traits, thus attracting the interest of botanists and hobbyists^9^. Many species of *Cymbidium* have been cultivated and hybridized as well-known ornamental orchids for many centuries. Here, a complete genome sequence is presented for *C. ensifolium*, an herbaceous orchid growing in shaded environments that exhibits a floral shape inclined to mutation, floral scent variation, and diverse leaf forms, making it a typical species of *Cymbidium*. The sequencing of the *C. ensifolium* genome revealed key innovations in the evolution of *Cymbidium* and provided novel and fundamental insights into the evolution and diversification of orchids.

## Results and discussion

### Genome sequencing and genome characteristics

**Fig. 1:**
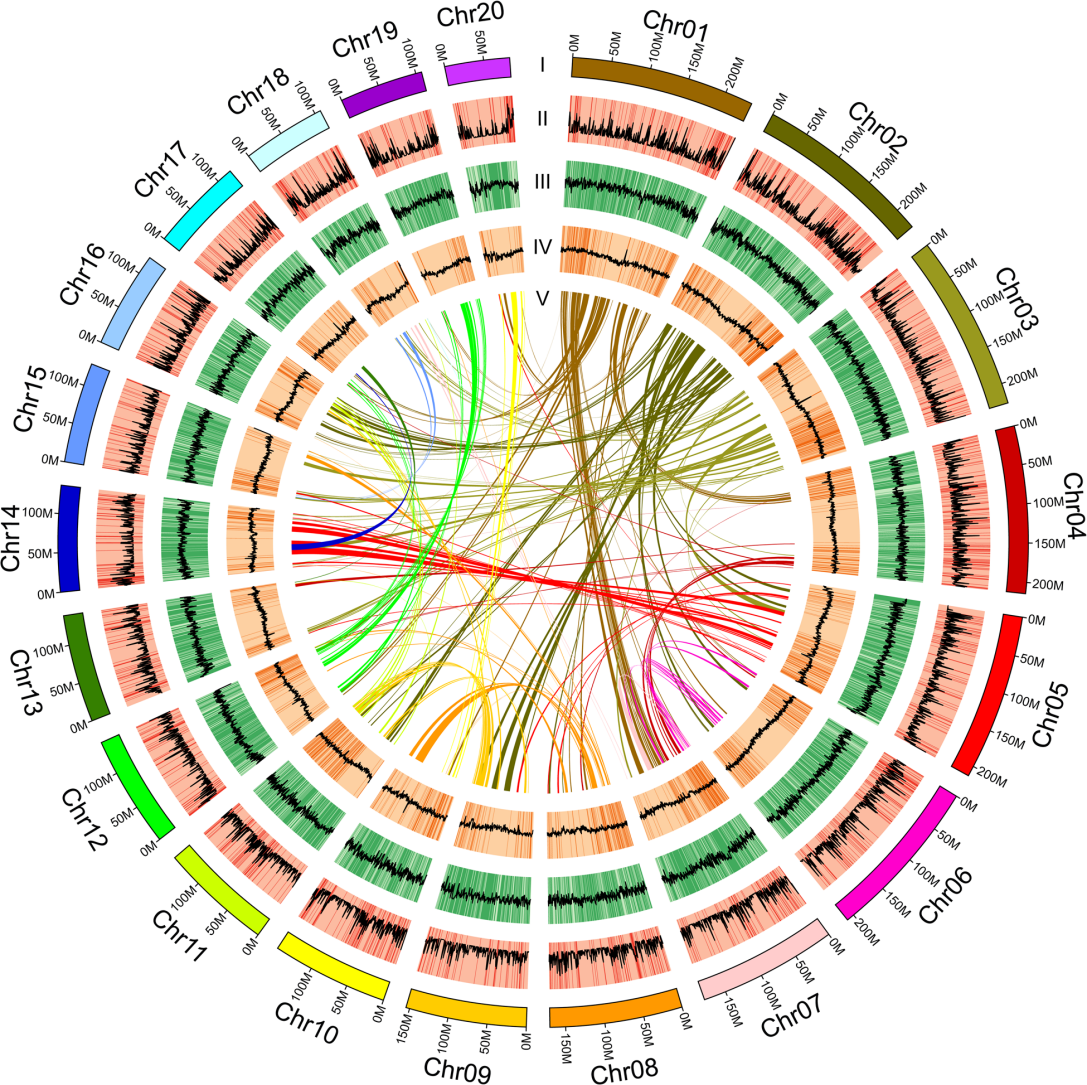
High-quality assembly of the *C. ensifolium* genome. **I** Chromosome numbers, **II** Gene density, **III** Repeat sequence density, **IV** GC content density, **V** Collinear regions. Each line connects a pair of homologous genes (from outside to inside)

*C. ensifolium* has a karyotype of 2 N = 2X = 40 with chromosomes of different lengths^10^. A total of 84.89 Gb of Illumina clean reads were obtained to assess the genome size of *C. ensifolium* (Supplementary Table 1). *K-*mer analysis showed that the genome size was 3.56 Gb, with a heterozygosity of 1.40% (Supplementary Fig. 1 and Supplementary Table 2). To obtain a better assembly, PacBio technology was employed, and 351 Gb of clean data was generated (Supplementary Table 3). The total length of the final assembly was 3.62 Gb, with a corresponding contig N50 value of 1.21 Mb (Supplementary Table 4). The Benchmarking Universal Single-Copy Orthologs (BUSCO)^11^ assessment indicated that the completeness of the assembled genome was 87.00% (Supplementary Table 5), and the Illumina read comparison rate was 98.87% (Supplementary Table 6). To assess the chromosome-level diploid genome, high-throughput/resolution chromosome conformation capture (Hi-C) technology was adopted. Based on 348.93 Gb of Hi-C clean reads, a 3.63 Gb genome was assembled, with a scaffold N50 value of 154.88 Mb (Supplementary Table 7). The 3.21 Gb of reads were anchored to 20 pseudochromosomes, and the length of pseudochromosomes ranged from 83.29 to 235.64 Mb (Supplementary Table 8). All of the contigs were mapped to the 20 pseudochromosomes. The chromatin interaction data suggested a high quality of our Hi-C assembly (Supplementary Fig. 2). These results indicated that the *C. ensifolium* genome assembly was relatively complete and presented high quality (Fig. 1).

### Gene prediction and annotation

A total of 29,073 protein-coding genes were confidently annotated in *C. ensifolium* (Supplementary Tables 9 and 10). BUSCO assessment indicated that the completeness of the genome was 78.19% (Supplementary Table 11). In addition to the higher number of genes in *C. ensifolium* than in *Apostasia shenzhenica*^2^, *Phalaenopsis equestris*^6^, *Dendrobium catenatum*^7^, and *Gastrodia elata*^8^ and most other angiosperms, the average length of genes and introns was also longer in *C. ensifolium* (Supplementary Fig. 3 and Supplementary Table 9). Considering that the average length of the gene sequences in *C. ensifolium* was longer than that in other angiosperms, a greater average intron length might be a unique feature of Orchidaceae, as it has previously also been observed in *A. shenzhenica*^2^, *P. equestris*^6^, *D. catenatum*^7^ and *G. elata*^8^ (Supplementary Fig. 3 and Supplementary Table 9). Among the 29,073 predicted genes, 28,739 genes (98.85%) could be functionally annotated, among which 26,803 were Kyoto Encyclopedia of Genes and Genomes (KEGG) terms and 25,721 were Gene Ontology (GO) terms, while only 334 genes could not be functionally annotated (Supplementary Table 12). Furthermore, 71 microRNAs, 2,018 transfer RNAs, 782 ribosomal RNAs and 139 small nuclear RNAs were identified (Supplementary Table 13).

Based on a combination of homology-based searches and *de novo* prediction, 80.58% of the *C. ensifolium* genome was estimated to consist of repetitive sequences (Supplementary Figs. 4 and 5, and Supplementary Table 14), which is a higher proportion than the 62% in *P. equestris*^6^, 78.10% in *D. catenatum*^7^, and 42.05% in *A. shenzhenica*^2^. Retrotransposable elements, which are the dominant form of repeats in angiosperm genomes, constituted a large part of the *C. ensifolium* genome (71.76%) and included the most abundant subtypes (Supplementary Table 15). In addition, the percentage of *de novo-*predicted repeats (70.30%) was notably higher than the percentage of repeats predicted based on Repbase11^12^ (Supplementary Table 15), indicating that the *C. ensifolium* genome contains more unique repeats than other sequenced orchid genomes; e.g., 57.88% in *P. equestris*^6^, 63.02% in *D. catenatum*^7^, and 39.35% in *A. shenzhenica*^2^. Among the combined transposable elements, long terminal repeats (LTRs) were dominant, accounting for 48.98% of the genome of *C. ensifolium*, which was greater than the repeated percentages of 22.06% in *A. shenzhenica*^2^ and 46% in *P. equestris*^6^ and *D. catenatum*^7^ (Supplementary Table 15).

### Evolution of gene families

**Fig. 2:**
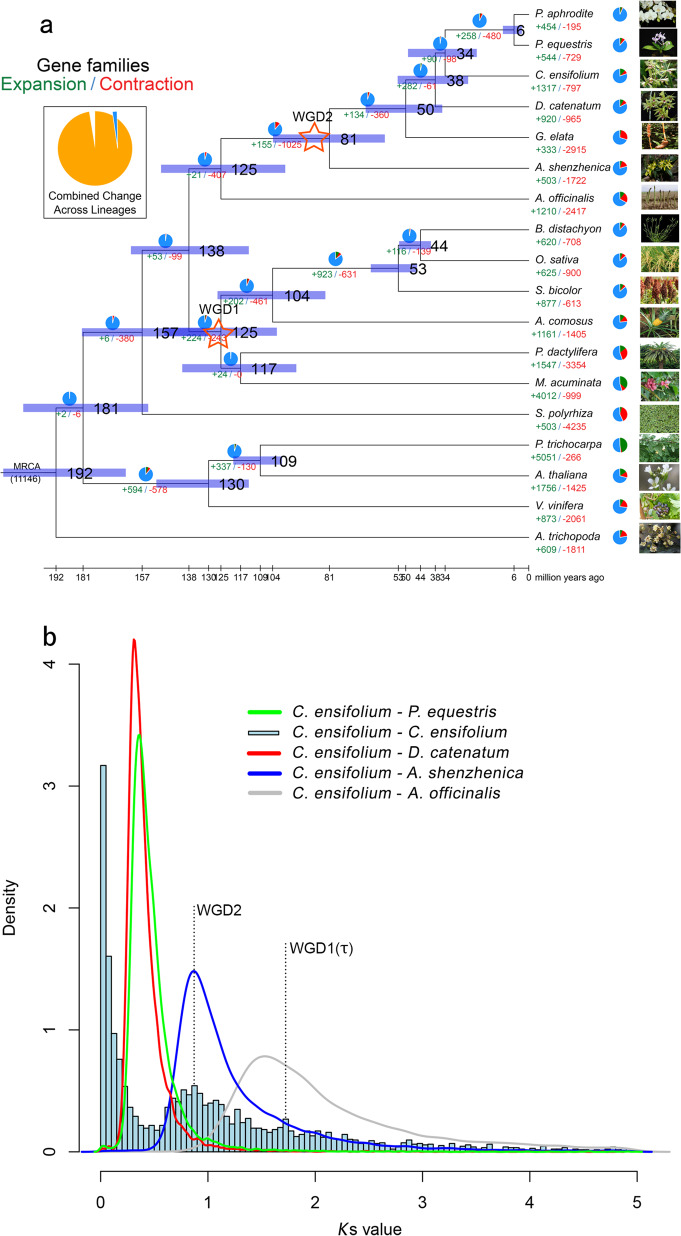
Evolution of gene families and whole-genome duplication (WGD) in *C. ensifolium*. **a** Expansion and contraction of gene families and phylogenetic relationships and divergence times between *C. ensifolium* and other plant species. The green numbers represent the numbers of expanded gene families, and the red numbers represent the numbers of contracted gene families. Blue in the circle indicates the gene families with a constant copy number, while orange indicates the proportions of 11,968 gene families of most recent common ancestors (MRCAs) that expanded or contracted during late differentiation. **b** *K*s distribution and WGD events in *C. ensifolium*. The *Ks* distribution of *C. ensifolium* showed two peaks, one at approximately 0.9 (WGD2), indicating that *C. ensifolium* experienced the last WGD event shared by all orchids, and another at approximately 1.7 (WGD1), which was likely the more ancient τ event shared by most monocots. The red stars represent the WGD events

A highly reliable phylogenetic tree was constructed, and the divergence times of 18 different plant species were estimated based on genes extracted from a total of 277 single-copy families (Supplementary Figs. 6 and 7, and Supplementary Table 16). As expected, *C. ensifolium* was placed as a sister group to the clade formed by *P. equestris* and *P. aphrodite* within the Orchidaceae clade (Supplementary Fig. 8). The estimated divergence time of Orchidaceae was 125 million years ago (Mya) (95% CI; 98–149 Mya). The divergence time between subfamilies Apostasioideae and Epidendroideae was 81 Mya (95% CI; 58–104 Mya). The divergence time between *C. ensifolium* and *D. catenatum* was 38 Mya (95% CI; 25–53 Mya), and the divergence time between *C. ensifolium* and *Phalaenopsis* was 34 Mya (95% CI; 21–49 Mya) (Fig. 2a). Furthermore, the expansion and contraction of orthologous gene families were determined. According to our analyses, 155 gene families were expanded in the lineage leading to Orchidaceae, whereas 1,025 gene families were contracted (Fig. 2a).

GO and KEGG enrichment analyses of the expanded gene families were performed, and significantly expanded gene families were shown to be especially enriched in the GO terms ‘oxidoreductase activity,’ ‘terpene synthase activity,’ ‘nitrogen compound transport,’ and ‘cellular lipid metabolic process’ and in the KEGG pathways ‘ABC transporters,’ ‘terpene backbone biosynthesis,’ ‘circadian rhythm’ and ‘fatty acid elongation’ (Supplementary Tables 17 and 18). Many terpene synthase (*TPS*) genes and genes involved in terpene backbone biosynthesis were included in the above enriched KEGG pathways and GO terms, in line with the biosynthesis of specific terpenoid scents of *C. ensifolium* flowers^13,14^. Additionally, we found that the significantly contracted gene families were especially enriched in the GO terms ‘myosin complex,’ ‘motor activity,’ ‘ATP binding,’ ‘ion binding’ and ‘protein binding’ (Supplementary Table 19).

The enrichment analyses also showed that the 786 unique gene families of *C. ensifolium* were specifically enriched in the GO terms ‘cysteine-type peptidase activity,’ ‘acetylglucosaminyltransferase activity,’ and ‘plastoquinol-plastocyanin reductase activity’ and in the KEGG pathways ‘thiamine metabolism,’ ‘cutin, suberin and wax biosynthesis’ and ‘RNA degradation’ (Supplementary Tables 16, 20 and 21).

The chloroplast and mitochondrial genomes of *C. ensifolium* were also assembled using the genome sequencing data. The chloroplast genome of *C. ensifolium* was 150,257 bp in length, which was consistent with previous results^15^. The mitochondrial genome was 766,026 bp in length, with 43 contigs. The genes that were lost from the chloroplast genome (*ndhF* and *ndhH*) were not found in the nuclear genome. Mitochondrial genes were not lost in *C. ensifolium*. Total DNA was extracted from flesh leaf cells, and plastid-like and mitochondrial-like reads were filtered in the genome assembly (Supplementary Fig. 9 and Supplementary Tables 22 and 23).

### Collinearity analysis and whole-genome duplication

Angiosperms are rife with whole-genome duplication (WGD) events^16^. Analyses of the *A. shenzhenica* genome showed that the most recent common ancestor of extant orchids experienced a WGD event^2^. Therefore, as expected, the distribution of synonymous substitutions per synonymous site (*K*_S_) for all paralogs in the *C. ensifolium* genome showed a peak suggestive of a WGD with a similar *K*_S_ value (~ 0.9) to that of other sequenced orchid genomes (Fig. 2b). Furthermore, relative to other published orchid genomes, the chromosome-level assembly of the *C. ensifolium* genome provides more solid evidence of synteny or collinearity supporting a WGD in orchids. Considering intragenomic collinearity, nearly all *C. ensifolium* chromosomal regions contained one other collinear region in the genome resulting from the orchid WGD, while some chromosomes, such as Chr02, had up to three such homologous collinear regions, providing support for an even more ancient WGD (Supplementary Fig. 10), likely the more ancient τ event shared by most monocots^17^.

The chromosome-level assembled *C. ensifolium* and *P. aphrodite* genomes^18^ allowed further comparisons of genomic changes after the divergence of *Cymbidium* and *Phalaenopsis*. Most chromosomes from the two species showed good one-to-one correspondence, but Chr02, Chr05, Chr11, and Chr16 in *C. ensifolium* shared orthologous collinear regions with more than one chromosome from *P. aphrodite*, suggesting the occurrence of chromosome fission or fusion after the divergence of the two species. Chromosome inversions were also observed between *C. ensifolium* and *P. aphrodite*, including inversions on Chr02, Chr06, Chr14, Chr17, Chr18, Chr19, and Chr20 (Supplementary Fig. 10b).

### MADS-box genes and the evolution of the orchid flower

#### Wild-type flowers and related genes

MADS-box genes are considered to be involved in many important processes of plant development, especially flower development. Since orchids are renowned for their flower morphology, we focused on the more detailed identification and characterization of MADS-box genes. A total of 71 putative functional MADS-box genes and 15 pseudogenes were identified in *C. ensifolium* (Table 1 and Supplementary Table 24). The number of functional MADS-box genes in *C. ensifolium* was higher than that in other genome-sequenced orchids^2,6,7^. *C. ensifolium* had 38 type II MADS-box genes, which was greater than the number found in *P. equestris* (29 members) and *A. shenzhenica* (27 members) and comparable to that in *D. catenatum* (35 members) (Table 1). Phylogenetic analysis (Supplementary Fig. 11) showed that most of the genes in the type II MADS-box clades had been duplicated, except for those in the B-PI, ANR1, and MADS32 clades, similar to the situation in other sequenced orchids. Among the duplicated type II clades, the B-class AP3 (four members), Bs (seven members), AGL6 (three members), and MIKC* clades (four members) contained more genes than in *A. thaliana* and rice^19,20^. However, genes from the FLC, AGL12, and AGL15 clades could not be found in *C. ensifolium* and other genome-sequenced orchids, suggesting that orthologs of FLC, AGL12, and AGL15 might have been specifically lost in orchids. Similar to the epiphytic orchids *P. equestris* and *D. catenatum*, terrestrial *C. ensifolium* has lost *AGL12*-like genes. Furthermore, the roots of *C. ensifolium* have a special outer tissue layer, the velamen radicum, similar to what is found in epiphytic *Phalaenopsis* and *Dendrobium*. Although *AGL12*-like genes (*XAL1* in *A. thaliana*) are necessary for root development and flowering^21^, a different mechanism seems to have evolved in *C. ensifolium* for root function. Genes in the B-class AP3 and AGL6 clades have been well studied in *P. equestris* and *Oncidium*^22,23^. These expanded clades, including members with differential expression patterns in orchid floral organs as well as divergent encoded protein domains, support the unique evolutionary routes of these floral organ identity genes related to unique lip innovation in orchids^22–25^. Combinatorial protein interaction networks among the members of the expanded B-AP3 and AGL6 clades might shape the unique novelties of orchid floral morphology.

**Table 1:** MADS gene families of four orchid species.

Category	*A. shenzhenica*		*P. equestris*		*D. catenatum*		*C. ensifolium*	
	Functional	Pseudo	Functional	Pseudo	Functional	Pesudo	Functional	Pesudo
Type II (Total)	27	4	29	1	35	11	38	7
MIKCc	25	3	28	1	32	9	34	
A	2		3		4		4	
AGL12	1		0		0		0	
C/D	4		5		4		4	
SOC1	2		2		2		3	
SVP	2		1		3		2	2
ANR1	4		2		3		1	
Bs	1		1		2		7	
B-PI	1		1		1		1	
AP3	2		4		4		4	
OsMADS32	1		0		1		1	
AGL6	2		3		3		3	4
E	3		6		5		4	1
FLC	0		0		0		0	
AGL15	0		0		0		0	
MIKC*	2	1	1	0	3	2	4	
Type I (Total)	9	0	22	8	28	1	33	8
Ma	5	0	10	6	15	1	27	
Mβ	0	0	0	0	0	0	0	
Mγ	4	0	12	2	13	0	6	
Total	36	4	51	9	63	12	71	15

Thirty-three putative functional type I MADS-box genes and eight pseudogenes were found in *C. ensifolium* (Table 1 and Supplementary Table 24). The increase in the number of type I genes in the α group (type I Mα) may have been caused by tandem gene duplication (Supplementary Fig. 12), suggesting that the type I MADS-box genes have experienced smaller-scale and more recent duplications^26^. Similar to the other genome-sequenced orchids, the *C. ensifolium* genome does not contain the β group of type I MADS-box genes (type I Mβ), although these genes do exist in *A. thaliana* and rice (Supplementary Fig. 13). This group of type I MADS-box genes may have been lost in the most recent common ancestor (MRCA) of orchids. The interaction among type I MADS box genes plays an important role in the initiation of endosperm development^27^.

**Fig. 3:**
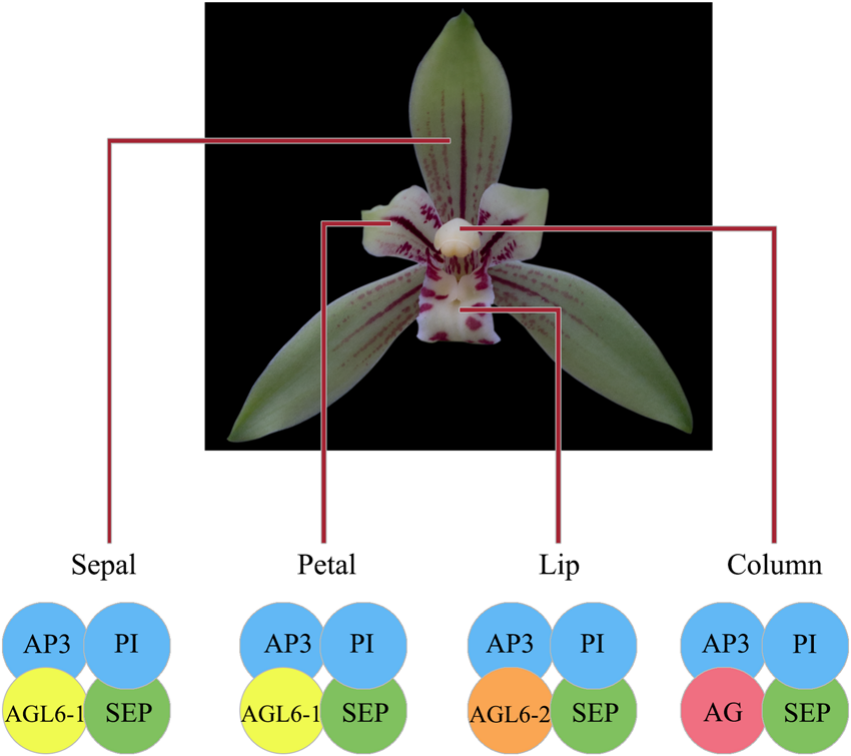
MADS-box model of *C. ensifolium* flowers. **AP3**: CeAP3-1, CeAP3-2, CeAP3-3, and CeAP3-4. **PI**: CePI. **SEP**: CeSEP-1, CeSEP-2, CeSEP-3, and CeSEP-4. **AGL6-1**: CeAGL6-1. **AGL6-2**: CeAGL6-2. **AG**: CeAG-1, CeAG-2, and CeAG-3. See Gene ID in Supplementary Table 24

The ABC model of floral organ development considers the formation of four-whorl floral organs in plants to be controlled by class A, B, and C genes. Class A genes are expressed in sepals and petals, class B genes are expressed in petals and stamens, and class C genes are expressed in stamens and pistils^28^. In *C. ensifolium*, the class C and D genes were only expressed in the column, while class A, B, and E genes were expressed in the three-whorl flower organs (Supplementary Figs. 14 and 15). Several other MADS-box genes were also expressed in various floral organs, such as *AGL6*-, *SOC1*-, and *OsMADS32*-like genes (Supplementary Figs. 14 and 15). These results indicate that the flower development model of Orchidaceae is not limited to the ABC model but does include characteristics of this model. Mondragón-Palomino and Theißen^29,30^ found that AP3/DEF genes had experienced two duplication events during evolution, forming four clades. Clade 1 and 2 genes are expressed in sepals, petals, and lips; clade 3 genes are expressed in petals and lips; and clade 4 genes are only expressed in lips. The ‘Homeotic Orchid Tepal’ (HOT) model considers all class B genes to be involved in determining the morphology of the orchid perianth in the early stage of flower primordium development, but the expression of class B genes in each whorl is restricted in the late stage of floral development^24^. All four clades of AP3/DEF genes were found to be expressed in the three-whorl flower organs (bud 1–5 mm long) of *C. ensifolium*, while one clade 3 AP3/DEF gene (*JL012707*) was mainly expressed in the petals, lips, and columns, and one clade 4 AP3/DEF gene (*JL000566*) was mainly expressed in the lips and columns in the mature flower organs of *C. ensifolium* (Supplementary Figs. 14 and 15). Notably, with the development of the flower organs, the expression of *AGL6* decreased. In mature flower organs, *CeAGL6-3* expression was very low in three-whorl flower organs, *CeAGL6-1* was mainly expressed in the sepals and petals, and *CeAGL6-2* was mainly expressed in the lips (Supplementary Fig. 15). The MADS-box model for *C. ensifolium* flowers is presented in Fig. 3. These results fit the model of perianth formation in orchids^23^.

#### *C. ensifolium* flower mutants and MADS-box genes

**Fig. 4:**
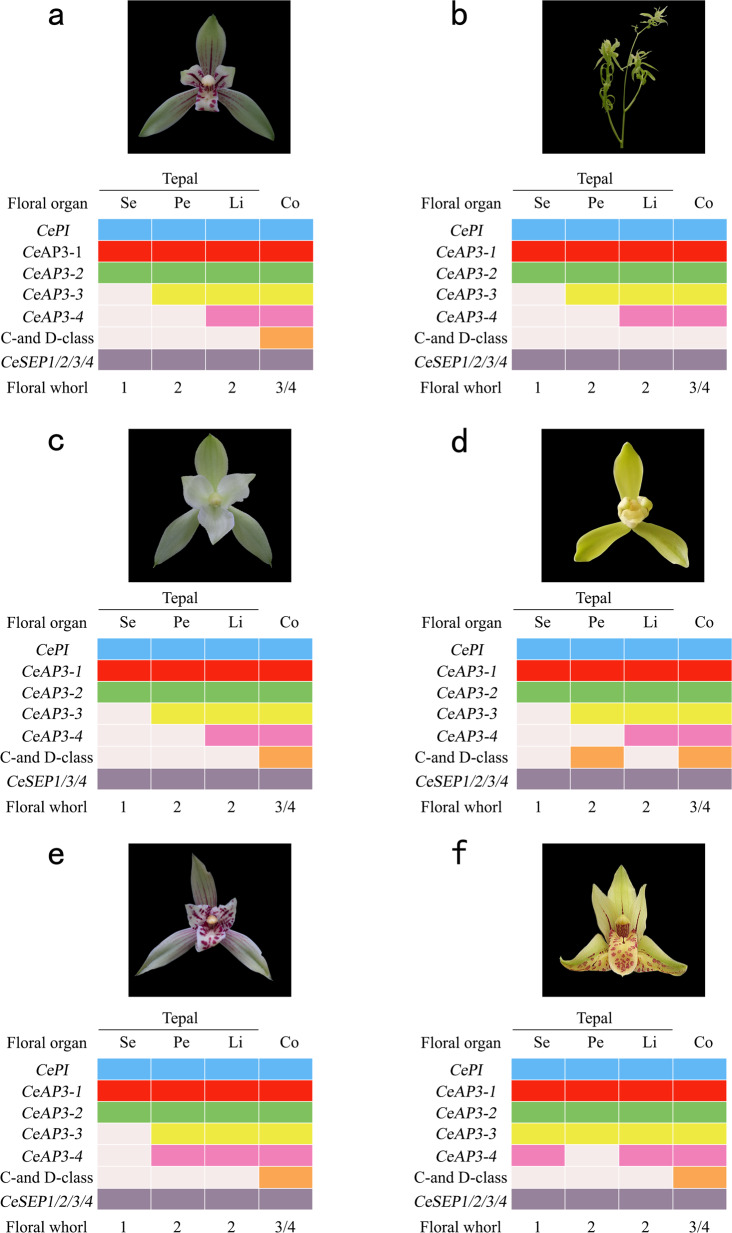
Flower morphology and the expression patterns of regulatory genes of different mutants of *C. ensifolium*. **a** Wild-type flowers and the expression patterns of regulatory genes^24^. **b** Branched inflorescence with multitepal flowers and the expression patterns of regulatory genes. **c** Peloric flower mutant and the expression patterns of regulatory genes. **d** Column-like petal mutant and the expression patterns of regulatory genes. **e** Lip-like petal mutant and the expression patterns of regulatory genes. **f** Lip-like sepal mutant and the expression patterns of regulatory genes. Se, sepal; Pe, petal; Li, lip; Co, column. The rectangles of different colors (blue, red, green, yellow, pink, orange and gray) indicate that the genes were expressed in the floral organs, while a white rectangle indicates that the gene was not expressed or was expressed at a low level in that floral organ. See Gene ID in Supplementary Table 24

The floral types of *C. ensifolium* are very diverse (Fig. 4). Based on morphological observations and transcriptome analysis, floral type mutations in *C. ensifolium* were found to be associated with the abnormal expression of MADS-box genes.

##### Branched inflorescence with multitepal flowers

The wild-type inflorescences of *C. ensifolium* were racemes, usually including 3–6 flowers. The wild-type flowers included three whorls of flower organs: the first whorl consisted of three sepals, the second whorl consisted of two petals and a lip, and the central whorl was a column (Fig. 4a). However, the branched inflorescence mutants showed a completely different morphology; they had indeterminant and branched inflorescences with multitepal flowers. The mutant flowers did not have a column instead of several whorls of perianths in the central whorl. The entire inflorescence formed a multibranched tree structure (Fig. 4b). The comparison of transcriptomic data between the wild-type and mutant floral organs indicated that the expression of C class genes (*CeAG-1* and *CeAG-2*) was dramatically decreased in the mutant flower buds (Supplementary Fig. 16). It has also been reported that the elimination of C class gene expression is responsible for multitepal flower generation in *Cymbidium*^31^.

##### Peloric flower mutant

Observations revealed that this mutant had three petals, did not show specialized lip differentiation, and displayed a peloric flower shape (Fig. 4c), similar to that of *A. shenzhenica*. It has been suggested that the adaxial petal does not differentiate into a specialized lip in *A. shenzhenica* due to the loss of class B-AP3 and E genes^2^. Our research revealed that there was no loss of B and E clade genes in *C. ensifolium* (Table 1 and Supplementary Table 24). Transcriptomic analysis showed that the expression levels of MADS-box genes in the peloric adaxial petals were the same and that all class B and E genes were expressed except for *CeSEP-2* (Supplementary Fig. 17). Therefore, it was suggested that the upregulation of *CeSEP-2* is important for the development of a specialized lip in *Cymbidium* orchids, while its downregulation results in the formation of a peloric flower shape in *C. ensifolium*.

##### Column-like petal mutant

Morphological observations showed that the petals of this mutant were transformed into a structure similar to a column, which was cylindrical, and it was defined as a column-like petal mutant (Fig. 4d). A previous study showed that C-class genes are mainly expressed in columns and participate in column formation without being expressed in normal petals^31^. However, transcriptomic analysis showed that the expression of the C-class genes *CeAG-1* and *CeAG-2* was increased significantly (heterotopic expression) in column-like petals. It was concluded that the ectopic expression of C-class genes at petal positions may contribute to this mutant phenotype (Supplementary Fig. 18).

##### Lip-like petal mutant

The petals of this mutant were transformed into a lip structure, so that there were three lips on a flower, and it was defined as a lip-like petal mutant (Fig. 4e). Previous studies have shown that the structure of lips is controlled by *CeAP3-1*, *CeAP3-2*, *CeAP3-3*, *CeAP3-4* and *CeAGL6-2*, while *CeAP3-1*, *CeAP3-2*, *CeAP3-3* and *CeAGL6-1* determine the formation of petals^22–24,29,30^. Transcriptomic analysis indicated that the expression of *CeAP3-3*, *CeAP3-4* and *CeAGL6-2* was upregulated in lip-like petal mutants relative to wild-type petals. The expression of the genes that control the lip flap on the petals caused this mutant phenotype (Supplementary Fig. 19).

##### Lip-like sepal mutant

The sepals of this mutant were partially transformed into a lip structure with bright spots, and it was defined as a lip-like sepal mutant (Fig. 4f). Transcriptomic analysis showed that there was low or no expression of the *CeAP3-3*, *CeAP3-4*, and *CeAGL6-2* genes in wild-type sepals. However, the expression of the *CeAP3-3*, *CeAP3-4*, and *CeAGL6-2* genes increased in lip-like sepals. Additionally, the expression of the *CeAP3-3*, *CeAP3-4*, and *CeAGL6-2* genes differed within a sepal, where high expression was associated with a higher degree of the lip and vice versa. The expression of the genes that control the lip flap on the sepals caused this mutant phenotype (Supplementary Fig. 20).

In conclusion, our results agree with the ‘Orchid Code’ model^29,30^ and the HOT model^23^. C class genes were only expressed in the column in the wild type. When the C class genes *CeAG-1* and *CeAG-2* showed high expression levels in the petals of *C. ensifolium*, the petals were transformed into column-like petals. When the expression of the C genes *CeAG-1* and *CeAG-2* in flower buds was dramatically decreased, the flower did not contain a column instead of several whorls of perianths and formed branched inflorescences with multitepal flowers. The upregulation of *CeSEP-2* was important for developing a specialized lip in orchids, while its downregulation allowed the formation of peloric flower shapes in *C. ensifolium*. The structure of the lips was controlled by the *CeAP3-1*, *CeAP3-2*, *CeAP3-3*, *CeAP3-4* and *CeAGL6-2* genes controlling the lip flap, but their expression in petals or sepals caused a lip-like petal mutant phenotype or a lip-like sepal mutant phenotype, respectively (Fig. 4).

### Floral fragrance biosynthesis

The emission of floral scents is an important strategy for ensuring fertilization^32^. The floral scent of *C. ensifolium* was analyzed by GC-MS. A total of 12 volatile substances were detected, mainly including fatty acids, monoterpenes and sesquiterpenes (Supplementary Table 25). There were three kinds of volatile substances with high relative contents: methyl jasmonate, acacia alcohol (sesquiterpene), and linalool (monoterpene), with relative contents of 12.10, 1.23, and 1.01%, respectively (Supplementary Table 25).

**Fig. 5:**
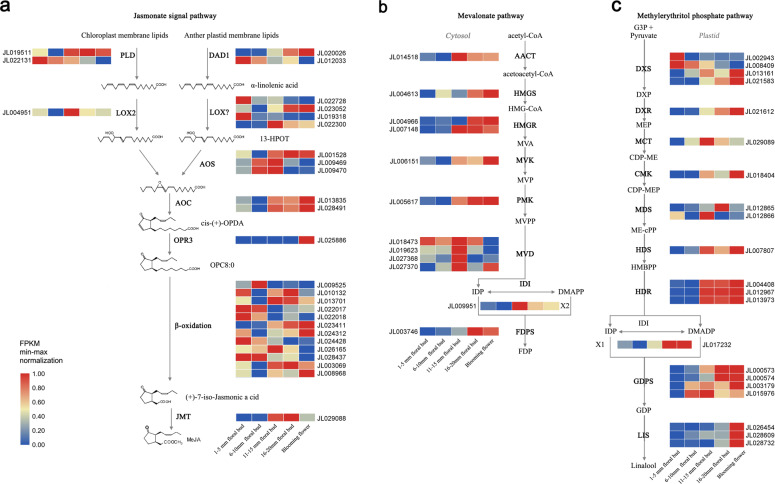
Expression levels of genes encoding enzymes involved in jasmonate biosynthesis and terpene backbone biosynthesis. **a** Jasmonate signal pathway^33^. **b** Mevalonate pathway^36^. **c** Methylerythritol phosphate pathway^36^. The heat map was plotted based on FPKM values, and min-max normalization was performed. Red indicates high levels of expression, while blue indicates low levels of expression. The abbreviated names of the enzymes (full name see Supplementary Table 26) involved in each catalytic step are shown in bold. The intermediate compounds in the pathways are OPDA, 12-oxo-phytodienoic acid; HMG-CoA, S-3-hydroxy-3-methylglutaryl-CoA; MVP, mevalonate-5-phosphate; MVPP, mevalonate diphosphate; CDP-ME, 4-diphosphocytidyl-2-C-methylerythritol; CDP-MEP, 4-diphosphocytidyl-2-C-methyl-D-erythritol 2-phosphate; DXP, 1-deoxy-D-xylulose 5-phosphate; G3P, glyceraldehyde-3-phosphate; HMBPP, 4-hydroxy-3-methyl-but-2-enyl pyrophosphate; and ME-cPP, 2-C-methyl-D-erythritol 2,4-cyclodiphosphate

The GC-MS results showed that methyl jasmonate accounted for the highest proportion of the volatile composition of *C. ensifolium* flowers. Through transcriptome analysis, high expression of genes related to the methyl jasmonate synthesis pathway was detected in flower buds and blooming flowers, indicating that methyl jasmonate (MeJA) production was developmentally regulated through *C. ensifolium* floral development (Fig. 5a). In addition, the expression of these genes was restricted to the perianth, suggesting that the production of MeJA in *C. ensifolium* flowers is floral organ specific (Supplementary Fig. 21). MeJA is a volatile signal involved in the response to many biotic and abiotic stresses (particularly herbivory and wounding)^33–35^. The synthesis of large amounts of MeJA at the perianth of mature flower buds and blooming flowers in *C. ensifolium* might be correlated with the protection of reproductive organs for successful pollination.

GC-MS results showed that volatile terpenoids, primarily monoterpenes (C10, e.g., linalool C_10_H_18_O) and sesquiterpenes (C15, e.g., acacia alcohol C_15_H_26_O), existed in the volatile components of *C. ensifolium* flowers (Supplementary Table 25). Genes related to the terpenoid biosynthesis pathways^36^ were identified from the genome, and the transcriptome data were used to analyze their expression in various floral developmental stages and different organs. In contrast to the low levels of expression observed in the roots, pseudobulbs, and leaves, the genes related to terpenoid backbone biosynthesis metabolic pathways were highly expressed in the mature floral buds, blooming flowers and perianths, indicating that volatile terpenoid compounds were mainly produced in flowers (Supplementary Fig. 22). In addition, the expression levels of three important genes that participate in the biosynthesis of monoterpenes or sesquiterpenes (geranyl diphosphate synthase (*GDPS*), farnesyl diphosphate synthase (*FDPS*), and linalool synthase (*LIS*)) reached a maximum in 15–20 mm flower buds and mature flowers, indicating that the production of monoterpenes and sesquiterpenes occurred mainly in the late stage of flower development and blooming (Fig. 5b, c). Furthermore, *FDPS* and *GDPS* were mainly expressed in the sepals, petals, and lips, indicating that terpenoids are mainly produced in the perianth (Supplementary Fig. 22). *LIS*, a key gene that participates in the generation of linalool, was highly expressed in floral organs and pseudobulbs (Supplementary Fig. 22). Our results supply information at the genomic level for understanding the molecular mechanisms underlying *C. ensifolium* floral scent production (Fig. 5).

### Evolution of the morphology of leaves

#### Colorful leaves

Yellow or white plaques and stripes on the leaves of *Cymbidium* orchids are common (Supplementary Figs. 23–25), and the potential molecular mechanisms underlying these patterns remain unclear. The color variation mechanism of *C. ensifolium* leaves was studied from the perspectives of chloroplast ultrastructure, chlorophyll content, and transcriptomic analysis. According to the mesophyll cell ultrastructure revealed by transmission electron microscopy, the chloroplast structures of wild-type leaves were located close to the cell membrane and had spindle and complete shapes, and the basal granular thylakoids were arranged neatly and tightly (Supplementary Fig. 23). However, in yellow leaves, the chloroplast structure differed significantly and showed an abnormal shape; the basal thylakoids were loosely arranged and disordered; and the number of osmiophilic granules was increased. As the degree of yellowing increased, the chloroplast structure became increasingly incomplete (Supplementary Fig. 23), suggesting that the colored leaves of *C. ensifolium* are a phenotypic mutation resulting from chlorophyll deficiency, and a decrease in the chlorophyll content is the direct cause of leaf color variation.

Chlorophyll is the main pigment in plants and is located in the thylakoid membrane, which contains two important protein complexes involved in photoreactions: photosynthetic systems I and II^37,38^. Transcriptomic analysis results showed that the expression levels of some genes related to photosynthesis-antennae and photosynthesis metabolic pathways in yellow leaves were significantly decreased (Supplementary Fig. 24). Similar results were obtained in white leaf tissues (Supplementary Fig. 25). Therefore, this study indicated that the reduced expression of genes related to photosynthesis leads to the inhibition of the synthesis of photosynthetic protein complexes, resulting in an incomplete chloroplast structure and hindering chlorophyll synthesis, thereby producing leaves of various colors.

### Perianth-like leaves

The leaves of this mutant had the appearance of a perianth (Supplementary Fig. 26). Transcriptomic analysis showed that the expression of MADS-box genes that are closely related to the development of floral organs was significantly higher in perianth-like leaves than in wild-type leaves (Supplementary Fig. 26).

In conclusion, the decreased expression of genes related to the photosynthesis-antennae and photosynthesis metabolic pathways led to the formation of colorful variegated leaves, while when the expression of MADS-box genes related to flower development was significantly increased in leaves, the leaves were transformed into perianth-like leaves.

## Materials and methods

### DNA preparation and sequencing

In this study, all materials used for genome sequencing were collected from an adult wild *C. ensifolium* plant growing in the Gushan Scenic Area, Fuzhou, Fujian Province. Total genomic DNA from young leaves was extracted using the hexadecyltrimethylammonium bromide (CTAB) method for Illumina and PacBio sequencing. The quality and purity of the extracted DNA were tested by using 0.75% agarose electrophoresis and a Nanodrop spectrophotometer, respectively. For Illumina sequencing, DNA was sonicated to a fragment size of 350 bp with an ultrasonicator, and the library was prepared according to Illumina’s instructions. Through a whole-genome shotgun (WGS) strategy, the paired-end sequencing of libraries was performed with an Illumina HiSeq Xten system. A total of 92.60 Gb of raw data were obtained, and unpaired reads, low-quality reads, connector contamination, and duplicated reads were filtered out to obtain clean data (Supplementary Table 1).

A 20-kb insert library was constructed based on the PacBio RSII protocol for PacBio sequencing. DNA was fragmented using a g-TUBE (Covaris), and magnetic beads were used to enrich and purify large fragments of DNA. The fragmented DNA was subjected to damage repair and end repair; stem circular sequencing adapters were connected at both ends of the DNA fragments; and exonuclease was used to remove fragments that failed to connect. An Agilent 2100 Bioanalyzer was used to detect the library fragment size. After the library was qualified, the PacBio Sequel platform was applied to perform 20 kb single-molecule real-time DNA sequencing. A high-quality region finder (HQRF) was applied to the obtained raw data to identify the longest region where the singly loaded enzyme remained active and filtered low-quality regions according to the signal noise ratio (SNR). Finally, a total of 351 Gb of clean data were obtained (mean read length 9.90 kb) (Supplementary Table 3).

### Assessment of genome size

Before genome assembly, the read information obtained by sequencing to estimate genome features was subjected to *K*-mer analysis to estimate the genome size and heterozygosity of *C. ensifolium*. The *K*-mer = 17 distribution map was constructed using Illumina reads. As shown in Supplementary Fig. 1, the average *K*-mer depth corresponding to the main peak was 21, and the genome size was inferred based on the formula ‘*K*-mer number/*K*-mer depth.’ Thus, the *C. ensifolium* genome size was estimated to be 3.56 Gb, with a 1.40% heterozygosity rate using GenomeScope (http://qb.cshl.edu/genomescope/)^39^.

### Genome assembly

Falcon and Wtdbg v1.2.8 (https://github.com/ruanjue/wtdbg)^40^ were used to calibrate and assemble PacBio clean reads, respectively. Briefly, the steps in this process were as follows: a. overlapping of raw subreads (FASTA) for error correction; b. preassembly and error correction; c. overlap detection among the error-corrected reads; d. overlap filtering; e. graph construction from the overlaps; f. contig construction from the graph. To obtain satisfactory assembly results, Wtdbg v1.2.8^39^ was used to select different parameters for assembly comparison among multiple versions, and the selected assembly parameters were ‘-k 0 -p 19 -S 2’. NextPolish (https://github.com/Nextomics/NextPolish)^41^ was then used to calibrate the assembled reference genome by using Illumina data to obtain a final assembled genome size of 3.62 Gb with a Contig N50 value of 1.21 Mb (Supplementary Table 4). In addition, BUSCO v5^11^ was used to assess the quality of the assembled genome (Supplementary Table 11), and SAMtools (http://samtools.sourceforge.net)^42^ was used to compare the short sequences obtained through Illumina sequencing with the assembled genome (Supplementary Table 4).

### Hi-C library construction and chromosome assembly

The Hi-C sequencing experiment mainly included the steps of cell cross-linking, endonuclease digestion, end repair, circularization, DNA purification and capture, and sequencing. A Hi-C sequencing library was created using high-quality DNA extracts from fresh leaves of *C. ensifolium*. The materials were then sequenced on the NovaSeq 5000 Platform. To obtain clean reads, the raw data were filtered with SOAPnuke v1.5.3^43^ (filtration parameters: filter -n 0.01 -l 20 -q 0.4 -d -M 3 -A 0.3 -Q 2 -i -G -seqType 1). Juicer^44^ was employed to compare the clean data with the genome. *Juicer* *+* *3d-dna*^45^ and *JuicerBox* (Juicebox_1.11.08)^44^ were used to cluster and adjust the genome sequences, respectively. To evaluate the Hi-C assembly results, the Hi-C assembly chromosome interaction heat map was constructed.

### Identification of repetitive sequences

Tandem Repeats Finder (v4.07b, http://tandem.bu.edu/trf/trf.html) was employed to predict tandem repeats^46^. Transposable elements (TEs) were first identified using RepeatMasker (http://www.repeatmasker.org, v3.3.0) and RepeatProteinMask based on the Repbase TE library (http://www.girinst. org/Repbase_Update.html)^12^. TE identification in the *C. ensifolium* genome was conducted using RepeatModeler (http://repeatmasker.org/RepeatModeler.html)^47^ and LTR_FINDER (http://tlife.fudan.edu.cn/ltr_finder/)^48^, with repeat sequence identities ≥ 50% grouped into the same classes.

### Gene prediction and annotation

The protein sequences of *Asparagus officinalis*, *D. catenatum*, *P. equestris*, *G. elata*, *A. shenzhenica*, and *Ananas comosus* were compared against the *C. ensifolium* genome using the TBLASTN algorithm. Augustus v3.0.2^49^, Genscan v3.1^50^ and GlimmerHMM^51^ were employed for *de novo* gene prediction. Subsequently, the results of the homology-based and *de novo* gene prediction were merged into a nonredundant gene set using EVM v1.1.1^52^ and MAKER2 (http://www.yandell-lab.org/software/maker.html)^53^. The transcriptome data were assembled by using TopHat v2.0.11^54^ and Cufflinks v2.2.1^55^, and the assembled dataset was used to supplement and perfect the obtained gene set. In addition, the GO (Gene Ontology Consortium), KEGG (http://www.genome.ad.jp/kegg/)^56^, InterPro (http://www.ebi.ac.uk/interpro/)^57^, Swiss-Prot (http://www.exoasy.ch/sport/andhttp://www.ebi.ac.uk/swissprot), and TrEMBL^58^ databases were used for the annotation of the predicted genes. The alignment of the rRNA template sequences from the Rfam database was carried out to identify rRNAs^59^. The tRNAs and other ncRNAs were predicted by using tRNAscan-SE and Infernal-0.81 software against the Rfam database, respectively^60^.

### Genome evolution analysis

OrthoMCL v2.0.9 was used to identify gene families in the genome^61^. Phylogenetic tree construction and divergence time estimation were based on peptide sequences from 277 single-copy gene families. Alignments obtained from MUSCLE v3.8.31 (http://www.drive5.com/muscle)^62^ were converted into coding sequences. A phylogenetic tree was constructed by using PhyML 4.7^63^. Species divergence times were estimated using the Bayesian relaxed molecular clock approach^64^. The ‘correlated molecular clock’ and ‘JC69’ models were used. Published tomato–potato (< 20 Mya, > 10 Mya) and papaya–*Arabidopsis* (< 90 Mya, > 54 Mya) data were used to calibrate divergence times.

The expansion and contraction analysis of orthologous gene families was conducted using the software CAFÉ 3 (https://github.com/hahnlab/CAFE)^65^. The expansion or contraction of gene families was analyzed using genome data from *C. ensifolium*, *Amborella trichopoda*, *Spirodela polyrhiza*, *D. catenatum*, *P. equestris*, *P. aphrodite*, *G. elata*, *A. shenzhenica*, *A. officinalis*, *A. comosus*, *Populus trichocarpa*, *Musa acuminate*, *Phoenix dactylifera*, *Brachypodium distachyon*, *Sorghum bicolor*, *Oryza sativa*, *Vitis vinifera*, and *A. thaliana*. Furthermore, a functional enrichment analysis of the genes of the significantly expanded and contracted gene families in the *C. ensifolium* genome was performed.

### Collinearity analysis and whole-genome duplication

Default parameters of JCVI v0.9.14 (https://pypi.org/project/jcvi/)^66^ were used to analyze the protein sequences of *C. ensifolium*, *P. aphrodite*, *A. officinalis*, *D. catenatum*, *P. equestris* and *A. shenzhenica* and to obtain gene pairs in collinear regions. *K*s (substitutions per synonymous site) distribution analysis was carried out to identify WGD events. DIAMOND (http://www.diamondsearch.org/index.php)^67^ was used for the self-alignment of the protein sequences of *C. ensifolium* and *P. equestris*, *P. aphrodite*, *D. catenatum*, *A. shenzhenica*, *C. ensifolium* and *A. officinalis* and to extract the mutually optimal alignments from the alignment results. The *K*s values were obtained with the Codeml program of the PAML package^68^.

### MADS-box gene family analysis

The MADS-box protein sequences of *A. thaliana* and the HMMER 3.0 profile (PF00319) were used to identify MADS-box transcription factors in *C. ensifolium*. HMMER 3.0 was built with the HMMER software package (version 3.0)^69^ using the seed alignment file, and HMMER 3.0 searches were performed against all predicted *C. ensifolium* proteins with an E-value threshold of 1e^−1^. The NCBI Conserved Domain Database (CDD, http://www.ncbi.nlm.nih.gov/Structure/cdd/wrpsb.cgi)^70^ was used to ensure the existence of the MADS domain. The Simple Modular Architecture Research Tool (SMART, http://smart.embl-heidelberg.de/) was used to further confirm the protein sequences^71^. The MADS-box protein sequences of *C. ensifolium* (Supplementary Data 1), *P. equestris*, *A. shenzhenica*, *A. thaliana* and *O. sativa* were then aligned using Clustal W 2.0, and MEGA 5^72^ was employed to construct an unrooted neighbor-joining phylogenetic tree.

### Transcriptome sequencing and expression analysis

Vegetative and floral organs were collected from the wild type and mutants of *C. ensifolium* (Supplementary Table 27) for RNA extraction by using the RNA Plant Plus Kit (Tiangen, DP473) and Illumina HiSeq 2500 sequencing. RNA extraction was conducted based on the manufacturer’s protocol. Illumina RNA-Seq libraries were prepared and sequenced on a HiSeq 2500 system following the manufacturer’s instructions (Illumina, USA). Gene expression levels were first estimated by using TopHat to map the clean reads of each sample onto the assembled genome. The obtained read counts for each gene were then normalized to FPKM reads^55^. The FPKM method was used to convert the number of read counts per gene into FPKM values representing the expression levels of the genes. Differential gene expression analysis was performed using DESeq based on the negative binomial distribution, and GO and KEGG enrichment analyses were conducted on the differentially expressed genes between samples. The FPKM values of genes were used to generate a heat map with TBtools v1.075^73^.

### Ultrastructural observations of mesophyll cells

Samples of the colorful mutant leaves and wild-type leaves of *C. ensifolium* with a volume not exceeding 1 mm × 1 mm × 1 mm were cut, placed in 2.5% glutaraldehyde and fixed at 4 °C for 4 h. After rinsing with 0.1 mol/L phosphate buffer solution (PBS, pH 7.4), the samples were sequentially submerged in 50, 70, 80, 90, 95, 100, and 100% ethanol for gradient dehydration for 15 min at each concentration. Subsequently, they were permeated overnight with a mixture of acetone and 812 embedding agent (1:1) and with pure 812 embedding agent and polymerized at 60 °C for 48 h. The samples were cut with a microtome to a thickness of 70 nm. Then, the sections were stained via uranium-lead double staining (2% saturated uranium acetate aqueous solution, lead citrate) for 15 min in each step and dried at room temperature overnight. Finally, the cells were observed under a transmission electron microscope (FEI TECNAI G2 20 TWIN), and images were recorded using imaging software.

### Detection of floral substances

The flowers of *C. ensifolium* were used as the materials for these analyses. The flowers were placed in a flask, soaked in anhydrous ethanol, and placed in a Soxhlet extractor equipped with a volatile oil extractor. The flowers were refluxed for 4 h and stored in a refrigerator. Gas chromatography (GC) analysis was performed on an HP6890 gas chromatograph using an FID detector. The experimental conditions were as follows: HP-5 MS capillary column (30 m × 0.25 mm × 0.025 mm); HP7683 autosampler; injection volume: 2 μL; quadrupole temperature: 230 °C; and program temperature: 70 °C for 2 min, then raised at 4 °C/min to 300 °C for 5 min. Gas chromatography-mass spectrometry (GC-MS) analysis was performed on an MSD5793 chromatographic-mass spectrometer. The GC conditions included high-purity nitrogen as a carrier gas and a flow rate of 1.0 mL/min, while other conditions were the same as the GC analysis conditions. The MS conditions were as follows: electron bombardment energy 70 eV; electron multiplier tube pressure 1380 V; scanning mass range 40–500; ion source temperature 250 °C. The collected mass spectrum data were searched and analyzed using the NIST library for the primary volatile oil.

## Conclusion

The genome of a typical oriental orchid, *C. ensifolium*, which has important ornamental and cultural value, was sequenced. The genome of *C. ensifolium* provides strong evidence of two WGD events: a more recent event shared by all orchids and an older event, most likely shared by most monocots. We identified 71 putative functional MADS-box genes and 15 pseudogenes in the genome of *C. ensifolium*. The lack of *AGL12*-like genes shows that *C. ensifolium* is not a terrestrial orchid in the classical sense but an epiphytic orchid that grows on the ground. Our results showed that the flower development model of Orchidaceae is not limited to the ABC model but shows characteristics of this model; this verified that the HOT model is applicable in *C. ensifolium*. Through the morphological observation and transcriptome analysis of mutants with different flower types, flower type mutants of *C. ensifolium* were associated with the abnormal expression of MADS-box genes. Fatty acids, monoterpenes, and sesquiterpenes were the main volatile substances found in *C. ensifolium*, while methyl jasmonate, acacia alcohol, and linalool presented the highest contents, and the fragrant scents of *C. ensifolium* were mainly produced in the perianth. The decreased expression levels of some genes related to the photosynthesis-antennae and photosynthesis metabolic pathways produced colorful striped leaves, and the significantly increased expression of MADS-box genes in leaves produced perianth-like leaves. Our results provide novel and fundamental insights into the origins, evolution, and diversification of orchids.

## Supplementary information

Supplementary information

## Data Availability

All *C. ensifolium* sequences described in this manuscript have been submitted to the National Genomics Data Center (NGDC). The raw genome sequences and raw transcriptome sequences have been deposited in BioProject/GSA under the accession codes PRJCA005355/CRA004327 and PRJCA005426/CRA004351, respectively. The results of the assembly and annotation of the whole-genome data have been deposited at BioProject/GWH under accession codes PRJCA005355/GWHBCII00000000.
