# Correction: The *Cymbidium* genome reveals the evolution of unique morphological traits

**DOI:** 10.1038/s41438-021-00709-6

**Published:** 2021-12-14

**Authors:** Ye Ai, Zhen Li, Wei-Hong Sun, Juan Chen, Diyang Zhang, Liang Ma, Qing-Hua Zhang, Ming-Kun Chen, Qing-Dong Zheng, Jiang-Feng Liu, Yu-Ting Jiang, Bai-Jun Li, Xuedie Liu, Xin-Yu Xu, Xia Yu, Yu Zheng, Xing-Yu Liao, Zhuang Zhou, Jie-Yu Wang, Zhi-Wen Wang, Tai-Xiang Xie, Shan-Hu Ma, Jie Zhou, Yu-Jie Ke, Yu-Zhen Zhou, Hsiang-Chia Lu, Ke-Wei Liu, Feng-Xi Yang, Gen-Fa Zhu, Laiqiang Huang, Dong-Hui Peng, Shi-Pin Chen, Siren Lan, Yves Van de Peer, Zhong-Jian Liu

**Affiliations:** 1 Key Laboratory of National Forestry and Grassland Administration for Orchid Conservation and Utilization at College of Landscape Architecture, Fujian Agriculture and Forestry University, Fuzhou, China; 2 Department of Plant Biotechnology and Bioinformatics, Ghent University, Gent, Belgium; 3 VIB Center for Plant Systems Biology, Gent, Belgium; 4 College of Forestry, Fujian Agriculture and Forestry University, Fuzhou, China; 5 Management Office of Yushan Scenic Area, Fuzhou, China; 6 College of Agriculture and Biotechnology, Zhejiang University, Hangzhou, China; 7 Key Laboratory of Plant Resource Conservation and Sustainable Utilization, South China Botanical Garden, Chinese Academy of Sciences, Guangzhou, China; 8 PubBio-Tech, Wuhan, China; 9 Tsinghua-Berkeley Shenzhen Institute (TBSI), Center for Biotechnology and Biomedicine and Shenzhen Key Laboratory of Gene and Antibody Therapy, State Key Laboratory of Chemical Oncogenomics, State Key Laboratory of Health Sciences and Technology, Institute of Biopharmaceutical and Health Engineering (iBHE), Shenzhen International Graduate School, Tsinghua University, Shenzhen, China; 10 Guangdong Key Laboratory of Ornamental Plant Germplasm Innovation and Utilization, Environmental Horticulture Research Institute, Guangdong Academy of Agricultural Sciences, Guangzhou, China; 11 Department of Biochemistry, Genetics and Microbiology, University of Pretoria, Pretoria, South Africa; 12 College of Horticulture, Academy for Advanced Interdisciplinary Studies, Nanjing Agricultural University, Nanjing, China; 13 Henry Fok College of Biology and Agriculture, Shaoguan University, Shaoguan, China; 14 Institute of Vegetable and Flowers, Shandong Academy of Agricultural Sciences, Jinan, China

**Keywords:** Genome duplication, Genome

Correction to: *Horticulture Research*

10.1038/s41438-021-00683-z published online 1 December 2021

After online publication of the article^1^ the author noticed minor typo in Fig. 4c. *CeSEP1/2/3/4* should be read as *CeSEP1/3/4*. Revised figure supplied during the proof correction was inadvertently not reproduced in the final version.

In addition, the affiliation numbers of the authors revised in the original article during proof correction, but not reflected in the title page in Supplementary Information.

The original article has been corrected.
