# Supplementary material for: The Cymbidium genome reveals the evolution of unique morphological traits
Source: Hortic Res. 2021 Dec 1;8:255. doi: 10.1038/s41438-021-00683-z (PMC8633000; doi:10.1038/s41438-021-00683-z)
Supplement: Supplementary file 1 — Supplementary information [file 41438_2021_683_MOESM1_ESM.docx]

**Supplementary Information**

**The *Cymbidium* genome reveals the evolution of unique morphological traits**

Ye Ai^1*^, Zhen Li^2,3*^, Wei-Hong Sun^4^, Juan Chen^1^, Diyang Zhang^1^, Liang Ma^1^, Qing-Hua Zhang^4^, Ming-Kun Chen^1^, Qing-Dong Zheng^1^, Jiang-Feng Liu^5^, Yu-Ting Jiang^4^, Bai-Jun Li^6^, Xuedie Liu^4^, Xin-Yu Xu^1^, Xia Yu^1^, Yu Zheng^4^, Xing-Yu Liao^4^, Zhuang Zhou^1^, Jie-Yu Wang^7^, Zhi-Wen Wang^8^, Tai-Xiang Xie^1^, Shan-Hu Ma^1^, Jie Zhou^1^, Yu-Jie Ke^1^, Yu-Zhen Zhou^1^, Hsiang-Chia Lu^1^, Ke-Wei Liu^9^, Feng-Xi Yang^10^, Gen-Fa Zhu^10^, Laiqiang Huang^9^, Dong-Hui Peng^1^, Shi-Pin Chen^4^, Siren Lan^1^, Yves Van de Peer^2,3,11,12†^, Zhong-Jian Liu^1,13,14†^

**Running title: The *Cymbidium* genome**

**Content**

[Supplementary Figures 1](#_Toc75011343)

[Supplementary Fig. 1 Genome size and heterozygosity estimation using a 17 *K*-mer distribution. 1](#_Toc75011344)

[Supplementary Fig. 2 Intensity signal heat map of Hi-C chromosome. 2](#_Toc75011345)

[Supplementary Fig. 3 Gene set statistics of gene structure prediction results. 3](#_Toc75011346)

[Supplementary Fig. 4 The sequence divergence rate of four different TEs using RepeatMasker annotation. 4](#_Toc75011347)

[Supplementary Fig. 5 The sequence divergence rate of four different TEs using *de novo* annotation. 5](#_Toc75011348)

[Supplementary Fig. 6 The Venn diagram shows the number of orthologous gene families in *C. ensifolium*, *P. equestris*, *G. elata* and *D. catenatum*. 6](#_Toc75011349)

[Supplementary Fig. 7 Orthologous genes in *C. ensifolium* and other species. 7](#_Toc75011350)

[Supplementary Fig. 8 Phylogenetic tree of 18 species. 8](#_Toc75011351)

[Supplementary Fig. 9 Chloroplast and mitochondrial genome annotation map of *C. ensifolium*. 9](#_Toc75011352)

[Supplementary Fig. 10 The gene pairs in the collinear region among *P. equestris*, *P. aphrodite*, *D. catenatum*, *A. shenzhenica*, *C. ensifolium* and *A. officinalis*. 10](#_Toc75011353)

[Supplementary Fig. 11 Phylogenetic tree of type II MADS genes of *C. ensifolium, P. equestris*, *A. shenzhenica*, *A. thaliana* and *O. sativa*. 11](#_Toc75011354)

[Supplementary Fig. 12 Chromosomal distribution and duplication of MADS-box genes in *C. ensifolium.* 12](#_Toc75011355)

[Supplementary Fig. 13 Phylogenetic tree of type I MADS-box genes of *C. ensifolium*, *P. equestris*, *A. shenzhenica*, *A. thaliana* and *O. sativa*. 13](#_Toc75011356)

[Supplementary Fig. 14 The expression of MADS-box genes in floral organs (bud 1–5 mm long) of *C. ensifolium*. 14](#_Toc75011357)

[Supplementary Fig. 15 The expression of MADS-box genes in mature floral organs of *C. ensifolium*. 15](#_Toc75011358)

[Supplementary Fig. 16 The expression of MADS-box genes in flower buds (1–10 mm long) of the branched inflorescence mutant and wild type of *C. ensifolium*. 16](#_Toc75011359)

[Supplementary Fig. 17 The expression of MADS-box genes in the petal and lip of the peloric flower mutant and wild type of *C. ensifolium*. 17](#_Toc75011360)

[Supplementary Fig. 18 The expression of MADS-box genes in column-like petal mutant and wild type of *C. ensifolium*. 18](#_Toc75011361)

[Supplementary Fig. 19 The expression of MADS-box genes in the lip-like petal mutant and wild type of *C. ensifolium*. 19](#_Toc75011362)

[Supplementary Fig. 20 The expression of MADS-box genes in lip-like sepal mutant of *C. ensifolium*. 20](#_Toc75011363)

[Supplementary Fig. 21 Expression analysis of genes related to the jasmonate synthesis pathway in various floral developmental stages and different organs of *C. ensifolium*. 21](#_Toc75011364)

[Supplementary Fig. 22 Expression analysis of genes related to the terpenoid synthesis pathway in various floral developmental stages and different organs of *C. ensifolium*. 22](#_Toc75011365)

[Supplementary Fig. 23 Morphology and ultrastructure of leaves in different colours of *C. ensifolium*. 23](#_Toc75011366)

[Supplementary Fig. 24 Differentially expressed genes related to photosynthesis-antennae and photosynthesis metabolic pathways in different colour leaves of *C. ensifolium*. 24](#_Toc75011367)

[Supplementary Fig. 25 Differentially expressed genes related to photosynthesis-antennae and photosynthesis metabolic pathways in green and white leaf tissues of the mutant of *C. ensifolium*. 25](#_Toc75011368)

[Supplementary Fig. 26 The expression of MADS-box genes in the perianth-like leaves and the wild-type leaves of *C. ensifolium*. 26](#_Toc75011369)

[Supplementary Tables 27](#_Toc75011370)

[Supplementary Table 1. The statistics of raw sequencing data from Illumina sequencing. 27](#_Toc75011371)

[Supplementary Table 2. *K*-mer statistics of genome sequencing results of *C. ensifolium.* 28](#_Toc75011372)

[Supplementary Table 3. The statistics of raw sequencing data from PacBio sequencing. 29](#_Toc75011373)

[Supplementary Table 4. Assembly statistics of the *C. ensifolium* genome 30](#_Toc75011374)

[Supplement Table 5. BUSCO assessment of the *C. ensifolium* assembled genome. 31](#_Toc75011375)

[Supplementary Table 6. Illumina sequence alignment statistics. 32](#_Toc75011376)

[Supplementary Table 7. The statistical results of Hi-C assembly. 33](#_Toc75011377)

[Supplementary Table 8. Chromosome length by Hi-C assembly. 34](#_Toc75011378)

[Supplementary Table 9. The prediction of gene structures of *C. ensifolium*. 35](#_Toc75011379)

[Supplementary Table 10. The number of protein-coding genes supported by *de novo*, transcriptome data and homology prediction. 36](#_Toc75011380)

[Supplementary Table 11. BUSCO assessment of the *C. ensifolium*’s genome annotation. 37](#_Toc75011381)

[Supplementary Table 12. The statistical results of functional annotation. 38](#_Toc75011382)

[Supplementary Table 13. Statistics on the annotation of non-coding RNA of the *C. ensifolium* genome. 39](#_Toc75011383)

[Supplementary Table 14. The statistical results of repeat sequences. 40](#_Toc75011384)

[Supplementary Table 15. Statistics of repeat sequences in *C. ensifolium*. 41](#_Toc75011385)

[Supplementary Table 16. Statistical results of clustered gene families. 42](#_Toc75011386)

[Supplementary Table 17. GO enrichment of significantly expanded (*p* <0.05) gene families of the *C. ensifolium* genome. 43](#_Toc75011387)

[Supplementary Table 18. KEGG pathway enrichment of significantly expanded (*p* <0.01) gene families of the *C. ensifolium* genome. 48](#_Toc75011388)

[Supplementary Table 19. GO enrichment of significantly contracted (*p* <0.05) gene families of the *C. ensifolium* genome. 49](#_Toc75011389)

[Supplementary Table 20. GO enrichment of unique gene families in the *C. ensifolium* genome. 50](#_Toc75011390)

[Supplementary Table 21. KEGG pathway enrichment of unique gene families in the *C. ensifolium* genome. 51](#_Toc75011391)

[Supplementary Table 22. Chloroplast genes of *C. ensifolium.* 52](#_Toc75011392)

[Supplementary Table 23. Mitochondrial genes of *C. ensifolium*. 53](#_Toc75011393)

[Supplementary Table 24. Analysis of the MADS-box genes of *C. ensifolium*. 55](#_Toc75011394)

[Supplementary Table 25. Volatile constituents of *C. ensifolium*’s flowers. 58](#_Toc75011395)

[Supplementary Table 26. The enzymes related to fragrance regulatory networks in *C. ensifolium*. 59](#_Toc75011396)

[Supplementary Table 27. The wild type and mutants of *C. ensifolium*. 60](#_Toc75011397)

[Supplementary Data 61](#_Toc75011398)

[Supplementary Data S1. MADS-box proteins of *C. ensifolium*. 61](#_Toc75011399)

[Supplementary References 68](#_Toc75011400)

# Supplementary Figures


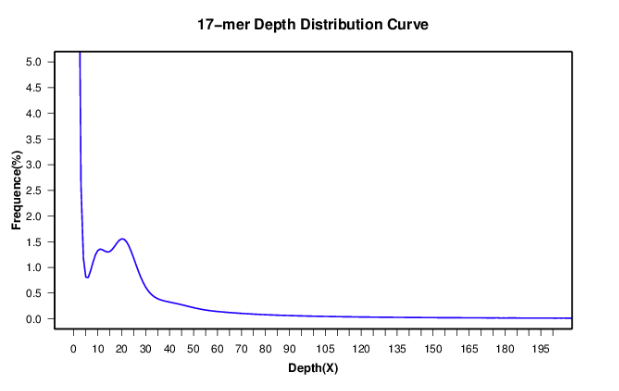


## Supplementary Fig. 1 Genome size and heterozygosity estimation using a 17 *K*-mer distribution.


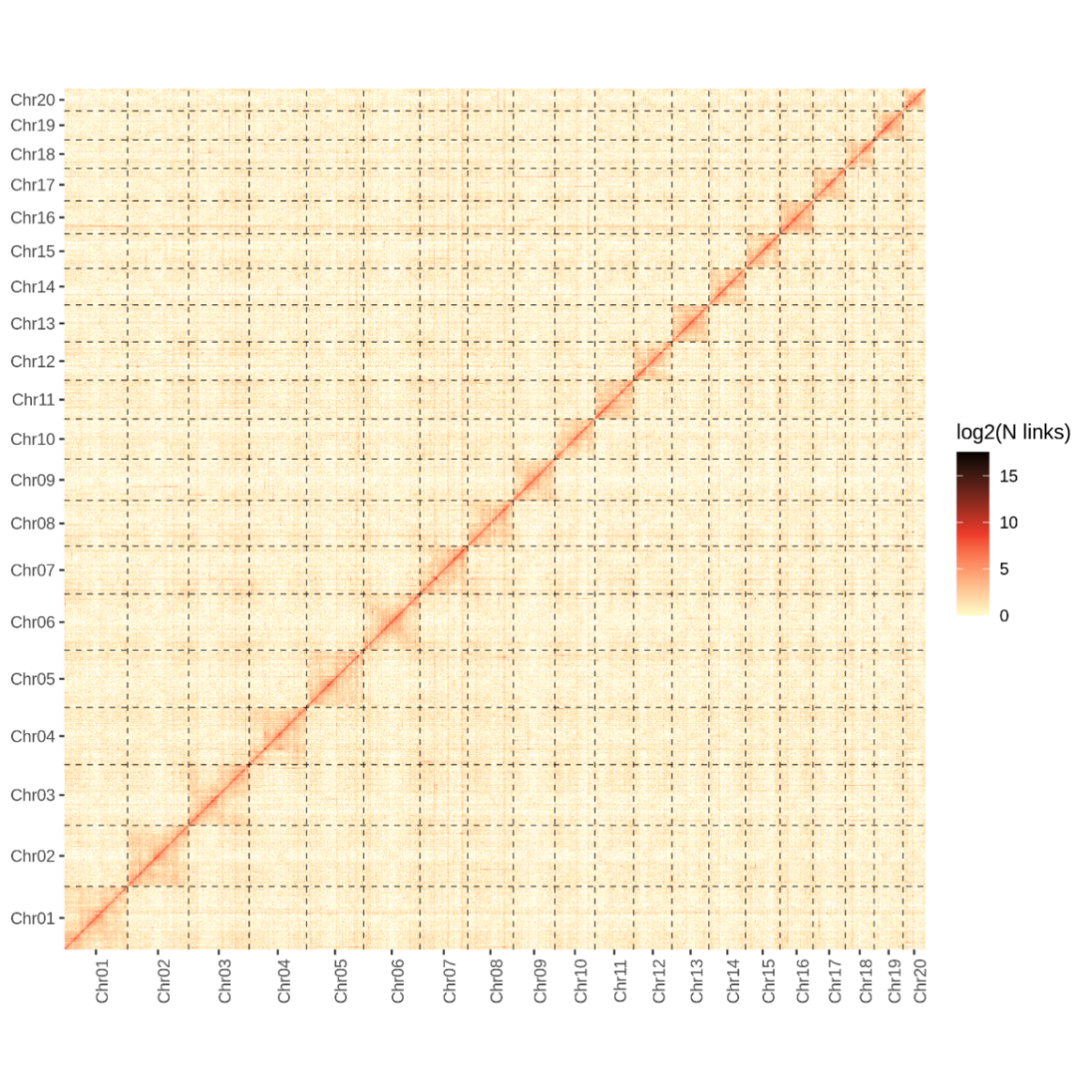


## Supplementary Fig. 2 Intensity signal heat map of Hi-C chromosome.


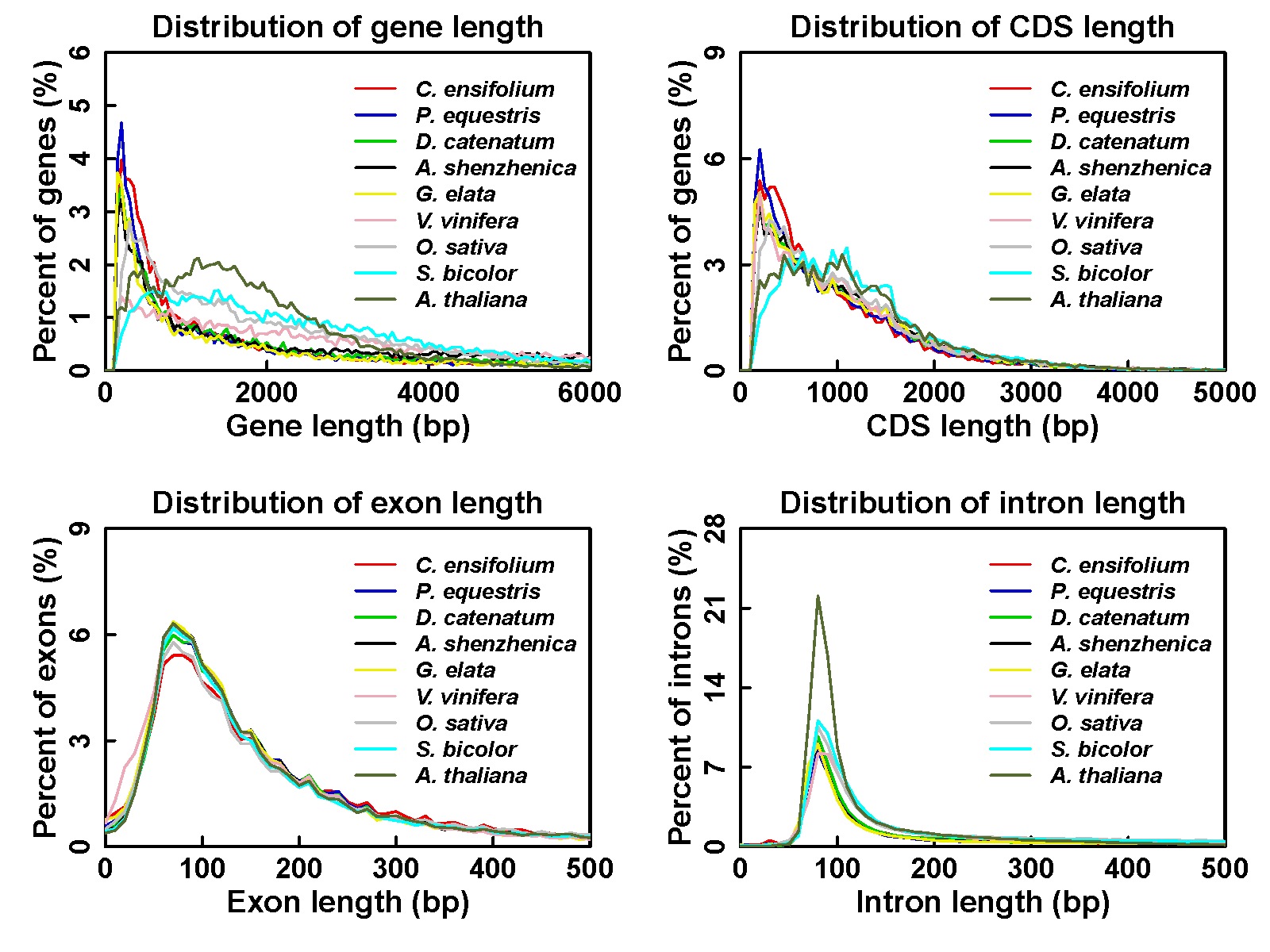


## Supplementary Fig. 3 Gene set statistics of gene structure prediction results. Window refers to the length represented by each point on the abscissa.

**
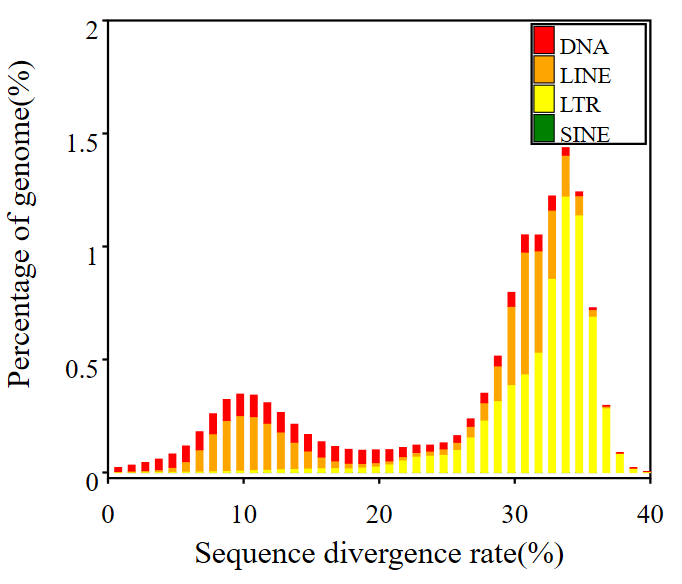
**

## Supplementary Fig. 4 The sequence divergence rate of four different TEs using RepeatMasker annotation.


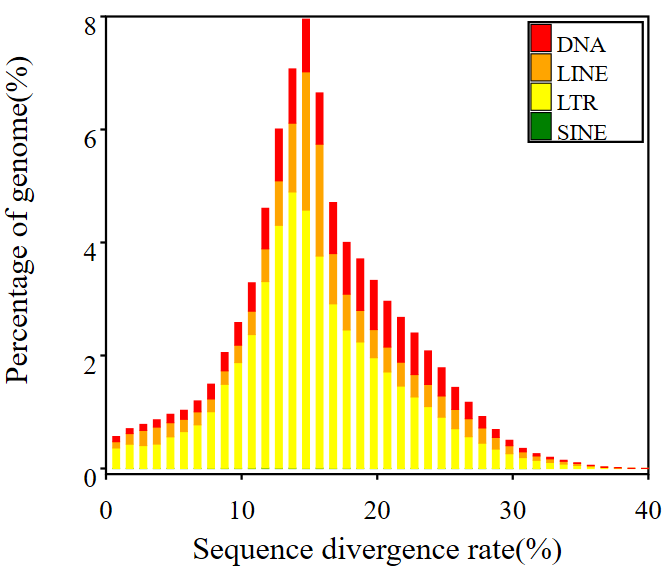


## Supplementary Fig. 5 The sequence divergence rate of four different TEs using *de novo* annotation.


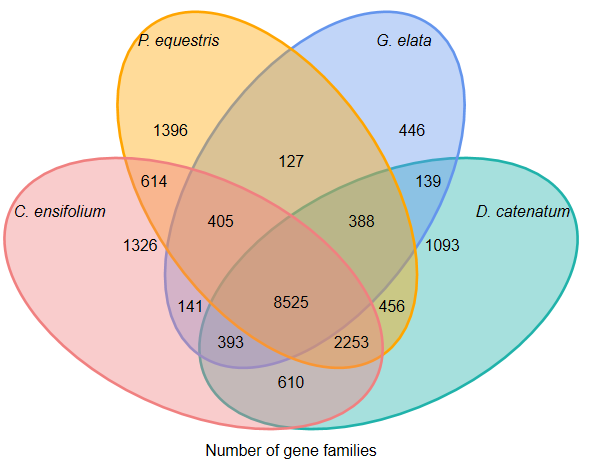


## Supplementary Fig. 6 The Venn diagram shows the number of orthologous gene families in *C. ensifolium*, *P. equestris*, *G. elata* and *D. catenatum*.


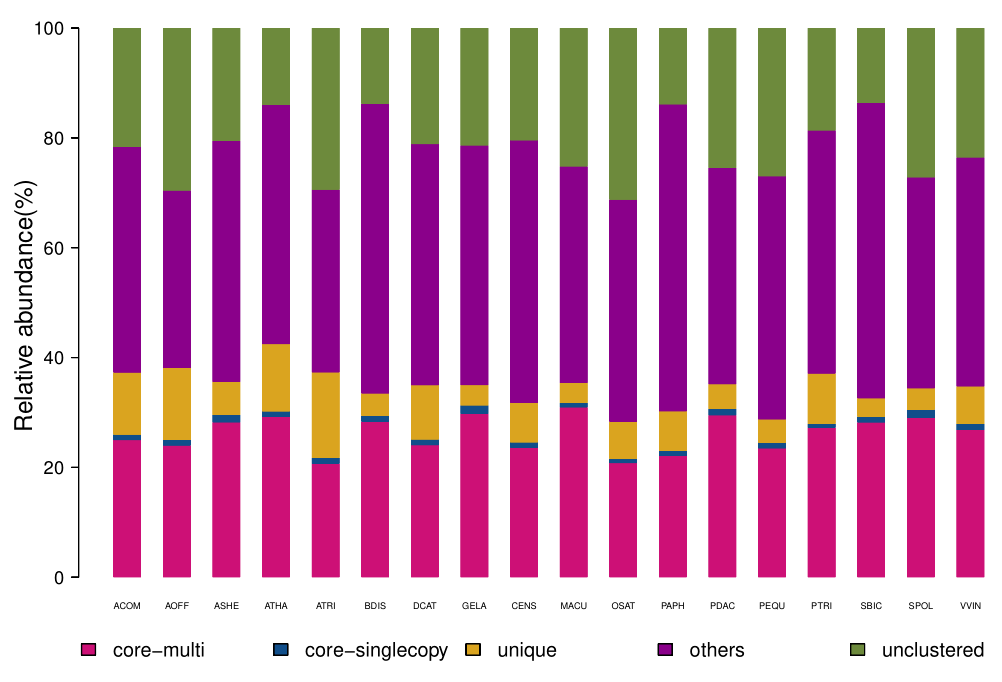


## Supplementary Fig. 7 Orthologous genes in *C. ensifolium* and other species. ACOM, *A. comosus*; AOFF, *A. officinalis*; ASHE, *A. shenzhenica*; ATHA, *A. thaliana*; ATRI, *A. trichopoda*; BDIS, *B. distachyon*; DCAT, *D. catenatum*; GELA, *G. elata*; CENS, *C. ensifolium*; MACU, *Musa acuminate*; OSAT, *Oryza sativa*; PAPH, *P. Aphrodite*; PDAC, *P. dactylifera*; PEQU, *P. equestris*; PTRI, *P. trichocarpa*; SBIC, *S. bicolor*; SPOL, *S. polyrhiza*; VVIN, *V. vinifera*.





## Supplementary Fig. 8 Phylogenetic tree of 18 species. Phylogenetic tree was constructed using phase 1 loci of orthologous genes, with each branch length representing the evolutionary rate.


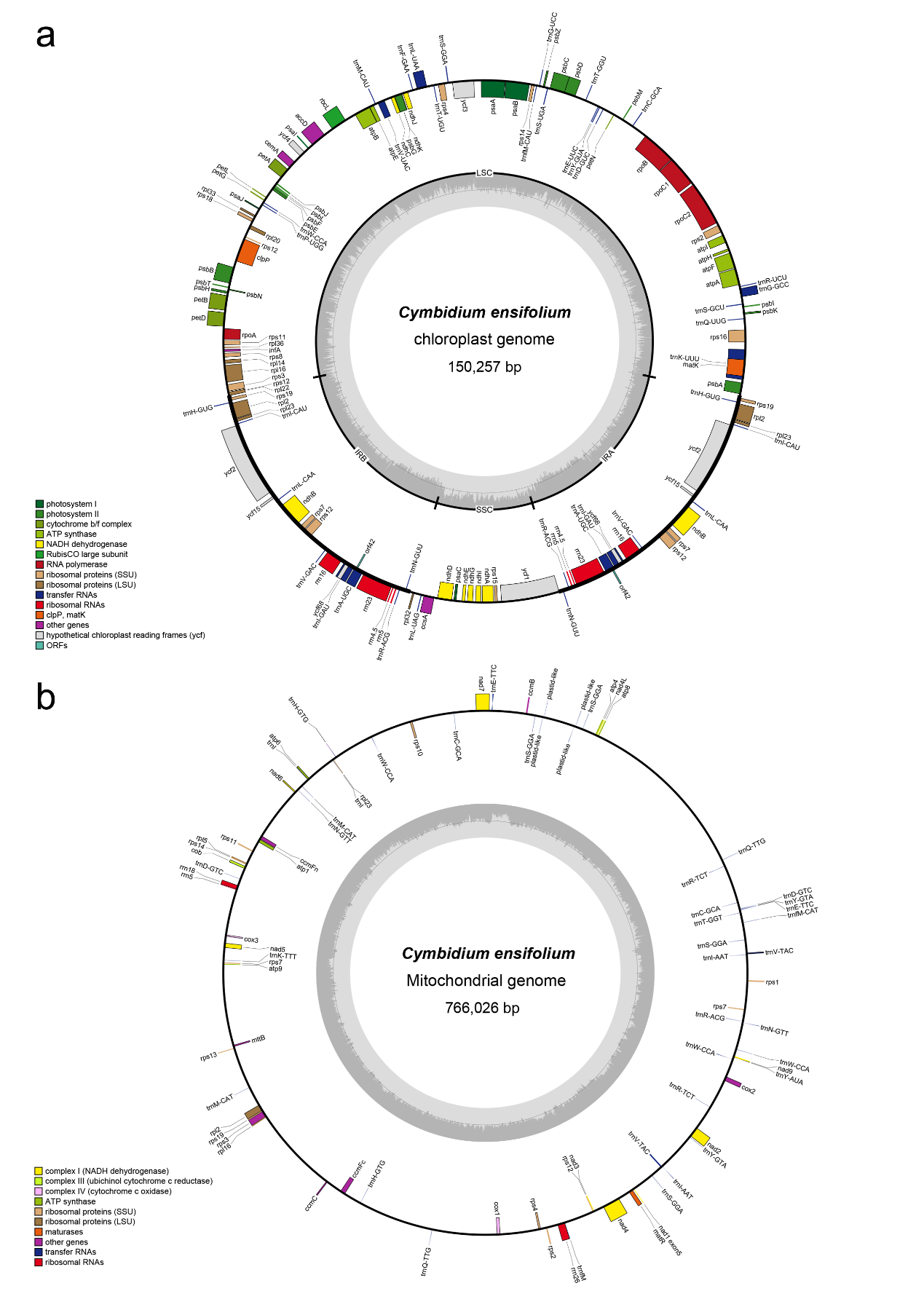


## Supplementary Fig. 9 Chloroplast and mitochondrial genome annotation map of *C. ensifolium*. a. The chloroplast genome annotation map of *C. ensifolium*. b. The mitochondrial genome annotation map of *C. ensifolium*.


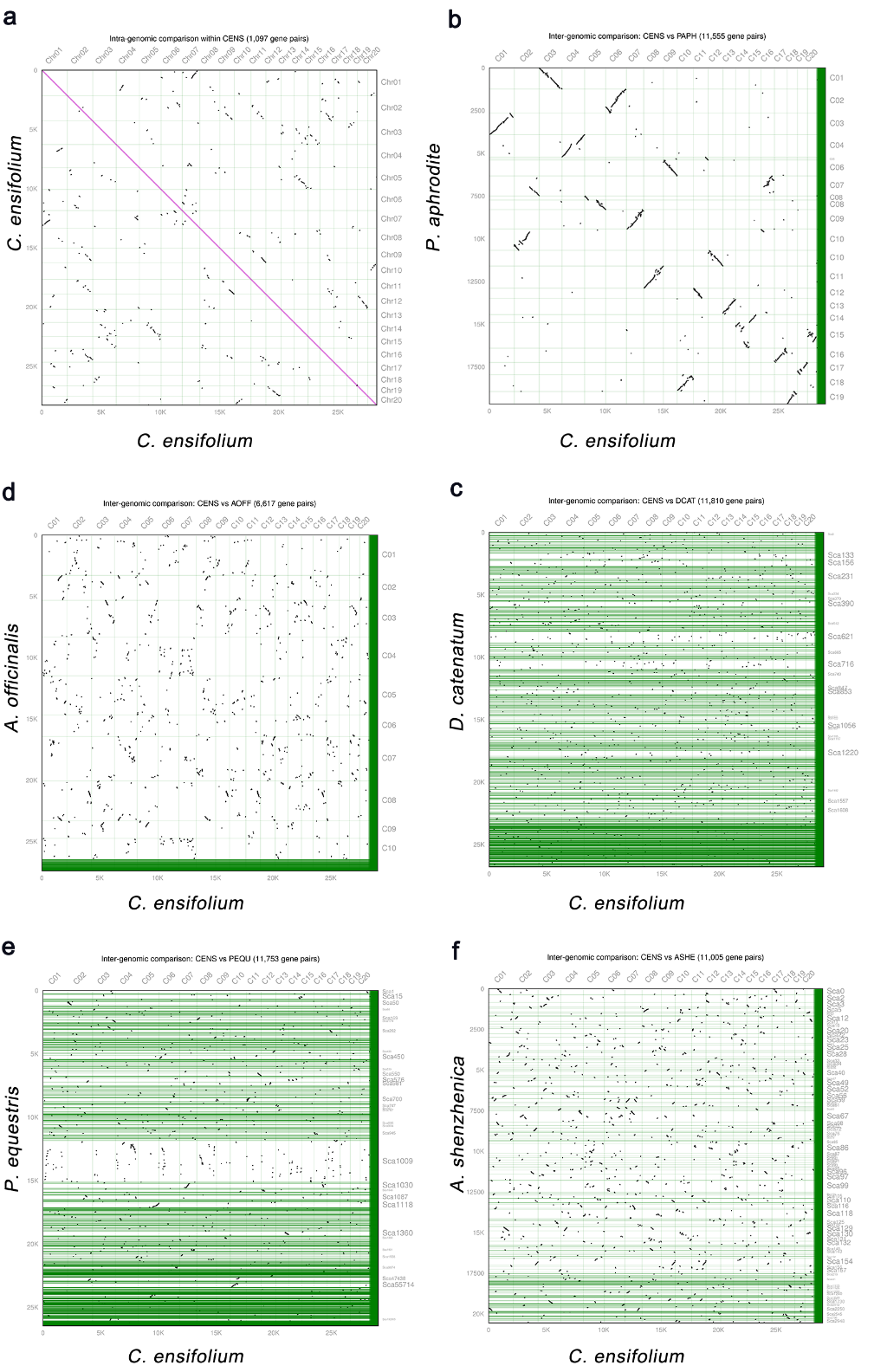


## Supplementary Fig. 10 The gene pairs in the collinear region among *P. equestris*, *P. aphrodite*, *D. catenatum*, *A. shenzhenica*, *C. ensifolium* and *A. officinalis*. a. The gene pairs in the collinear region of *C. ensifolium* and *C. ensifolium*. b. The gene pairs in the collinear region of *C. ensifolium* and *P. aphrodite*. Chromosomes 5, 8 and 10 were each resolved into two linkage groups in the physical mapping of *P. aphrodite* genome^1^. c. The gene pairs in the collinear region of *C. ensifolium* and *A. officinalis*. d. The gene pairs in the collinear region of *C. ensifolium* and *D. catenatum*. e. The gene pairs in the collinear region of *C. ensifolium* and *P. equestris*. f. The gene pairs in the collinear region of *C. ensifolium* and *A. shenzhenica*.





## Supplementary Fig. 11 Phylogenetic tree of type II MADS genes of *C. ensifolium, P. equestris*, *A. shenzhenica*, *A. thaliana* and *O. sativa*.


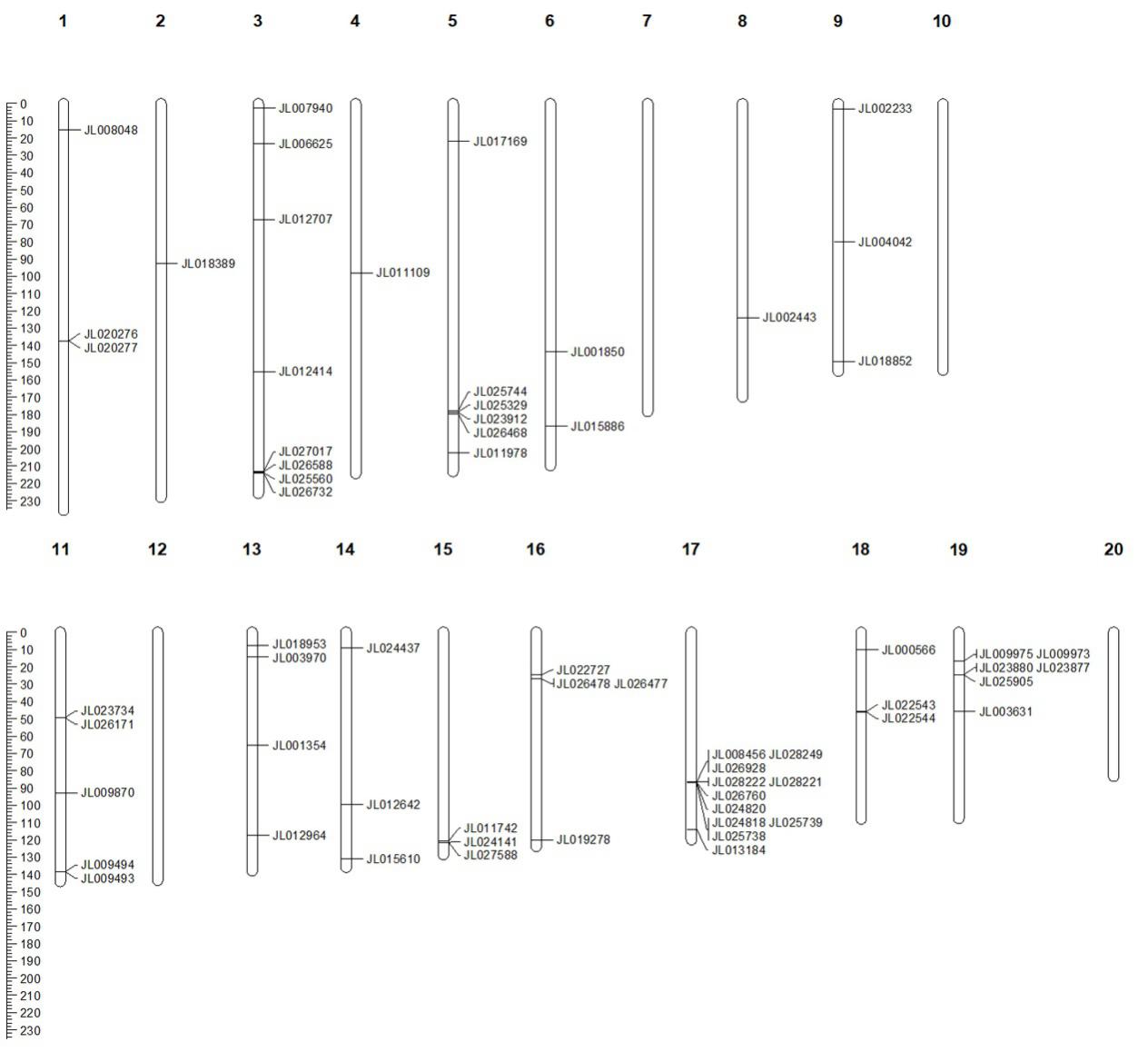


## Supplementary Fig. 12 Chromosomal distribution and duplication of MADS-box genes in *C. ensifolium.*





## Supplementary Fig. 13 Phylogenetic tree of type I MADS-box genes of *C. ensifolium*, *P. equestris*, *A. shenzhenica*, *A. thaliana* and *O. sativa*.


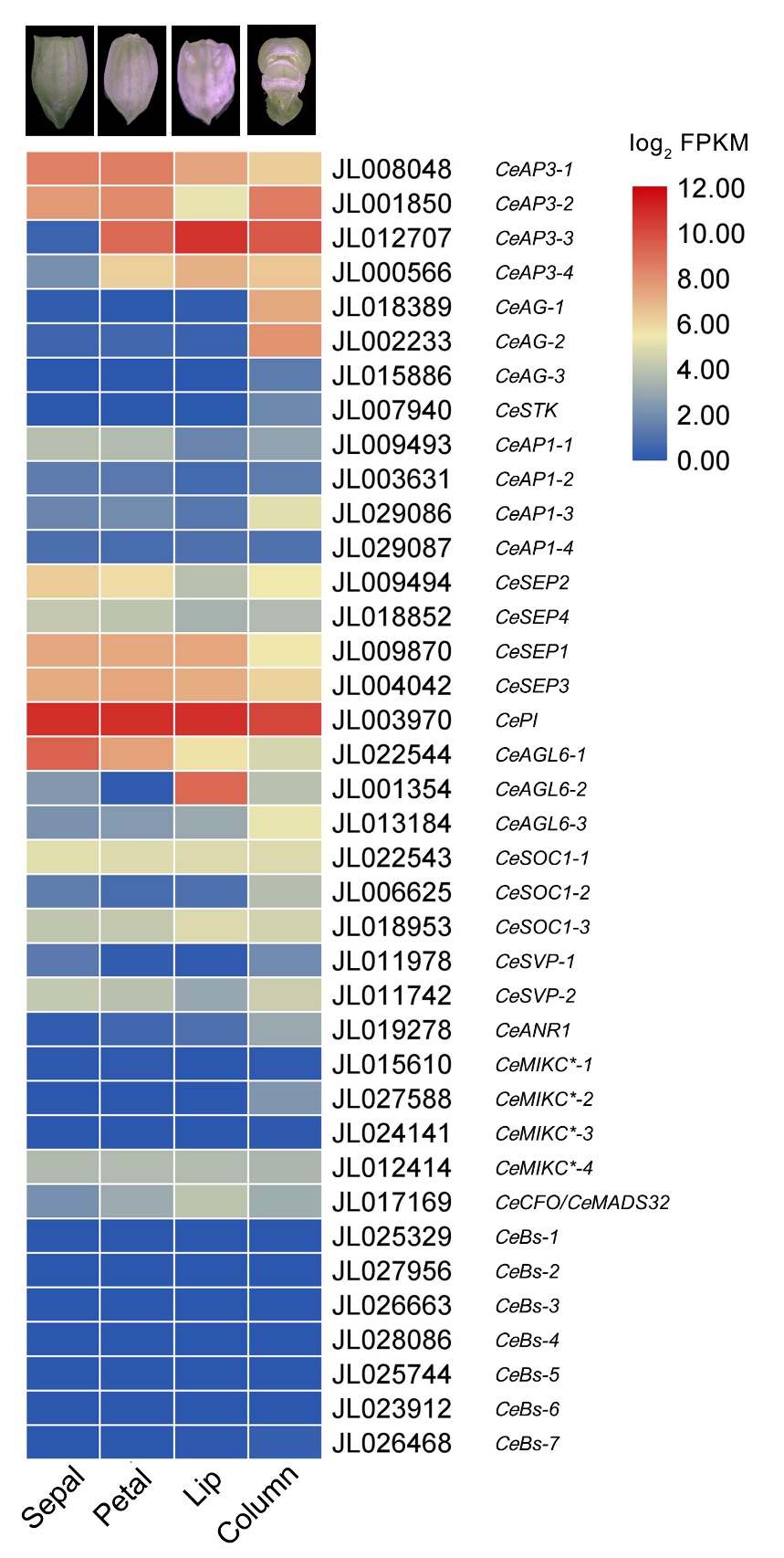


## Supplementary Fig. 14 The expression of MADS-box genes in floral organs (bud 1–5 mm long) of *C. ensifolium*.


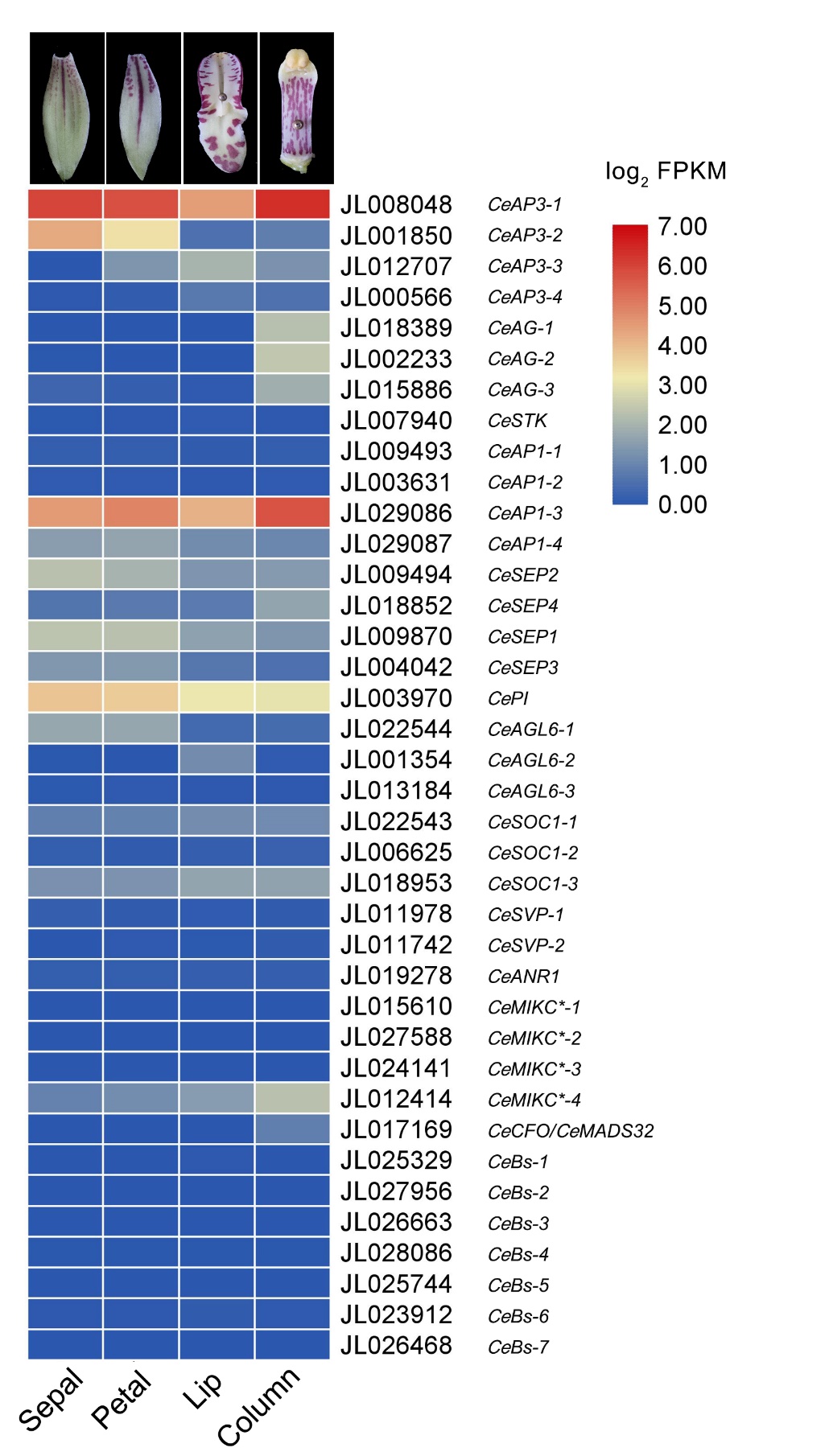


## Supplementary Fig. 15 The expression of MADS-box genes in mature floral organs of *C. ensifolium*.


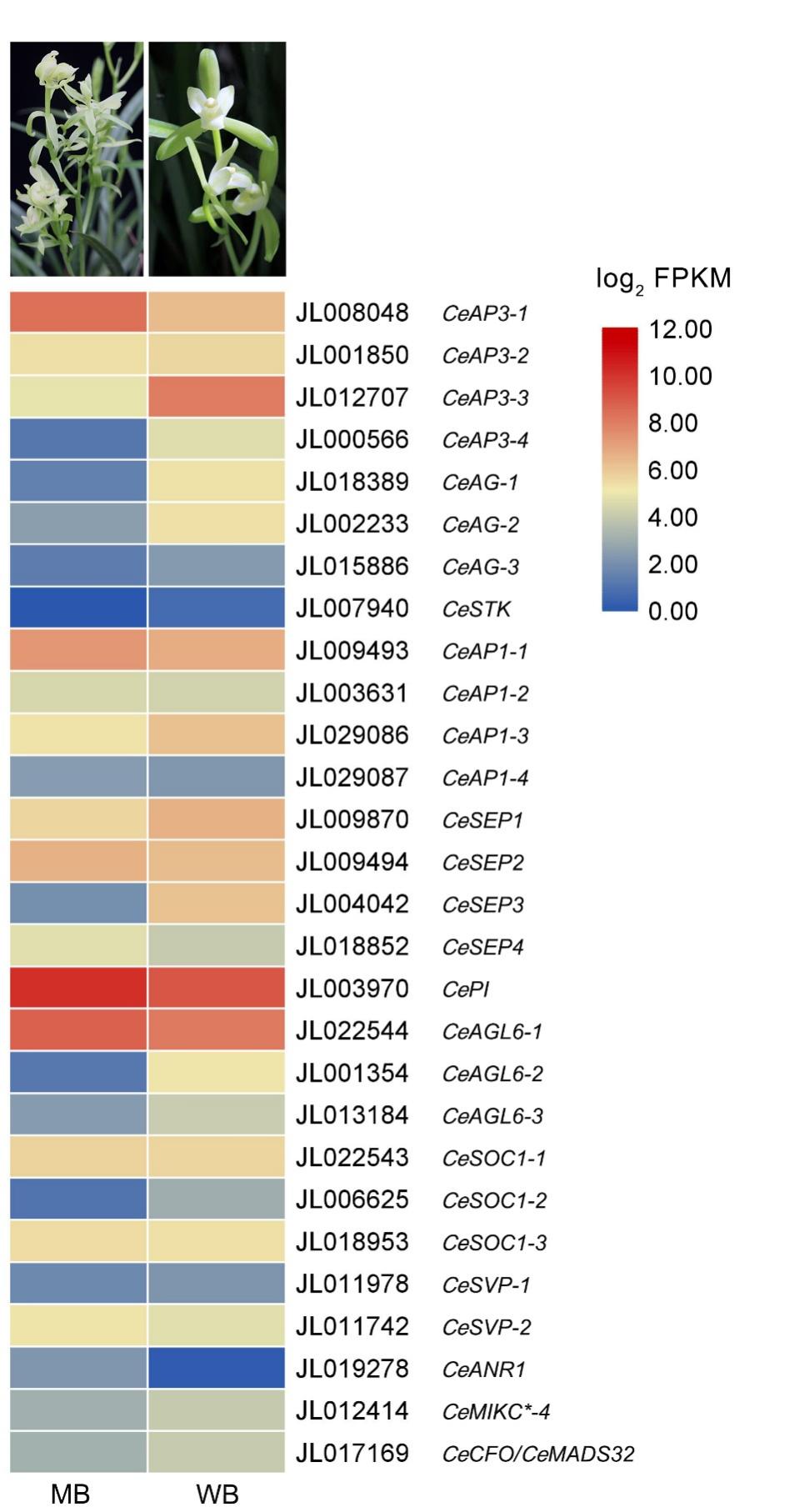


## Supplementary Fig. 16 The expression of MADS-box genes in flower buds (1–10 mm long) of the branched inflorescence mutant and wild type of *C. ensifolium*. MB, flower buds (1–10 mm long) of branched inflorescence mutant of *C. ensifolium*; WB, flower buds (1–10 mm long) of wild type of *C. ensifolium*.


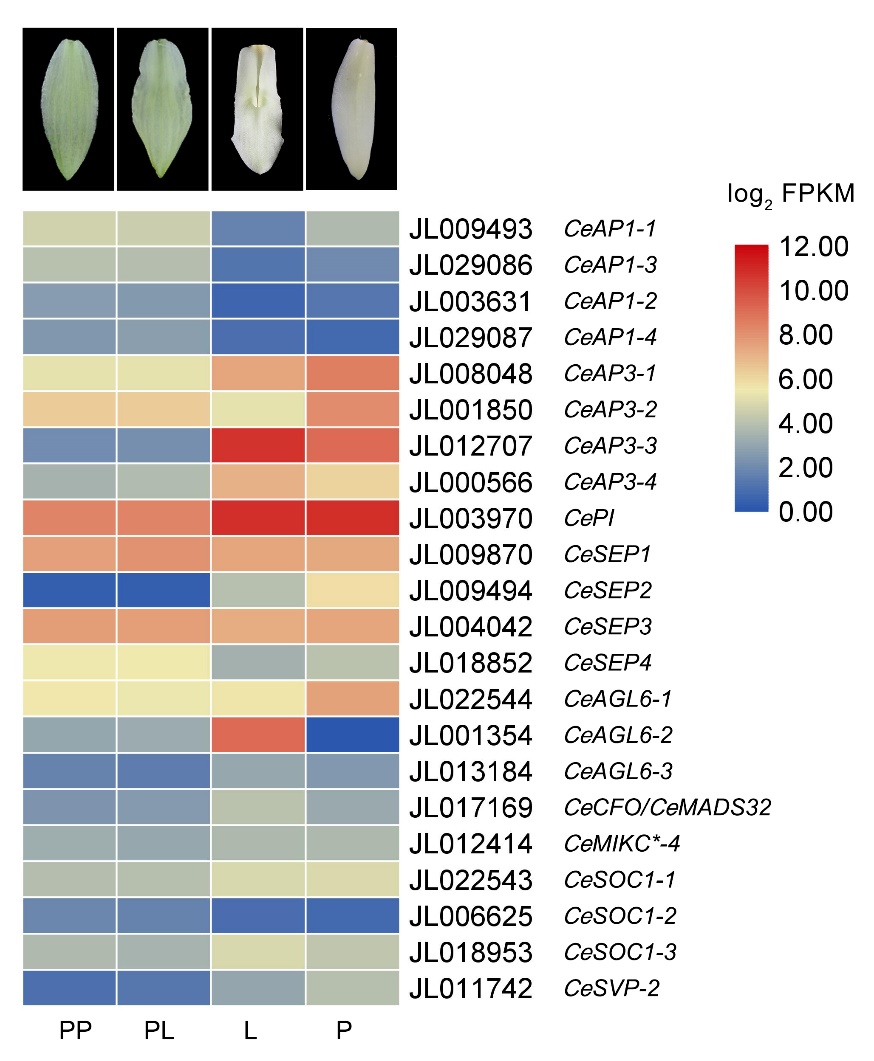


## **Supplementary Fig. 17 The expression of** MADS-box genes **in the petal and lip of the peloric flower mutant an**d wild type of *C. ensifolium***.** PP, petal of the peloric flower mutant; PL, lip of the peloric flower mutant; L, lip of the wild type; P, petal of the wild type.


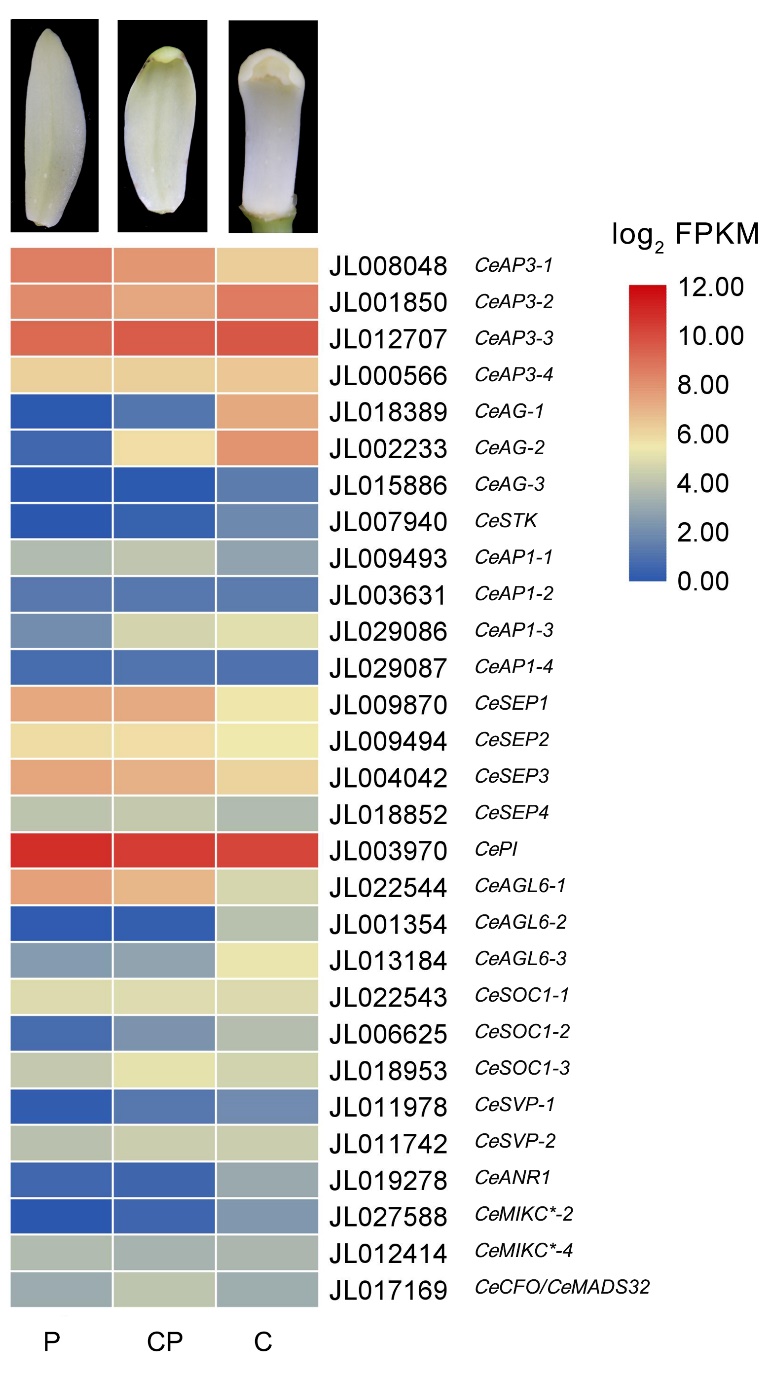


## **Supplementary Fig. 18 The expression of MADS-box genes in column-like petal mutant** and wild type of ***C. ensifolium*.** P, petal of the wild type; CP, column-like petal of the mutant type; C, column of the wild type.


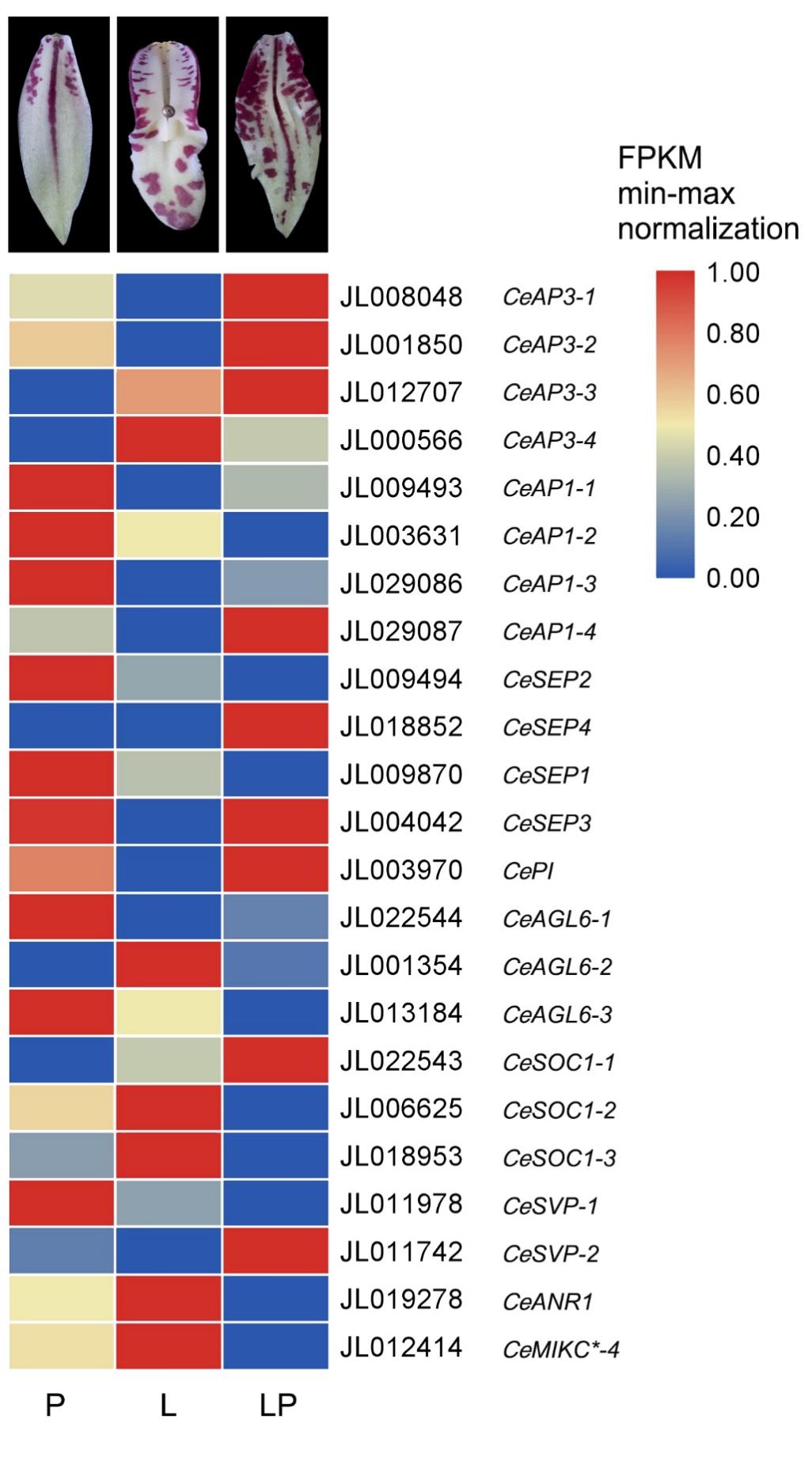


## Supplementary Fig. 19 The expression of MADS-box genes in the lip-like petal mutant and wild type of *C. ensifolium*. P, petal of the wild type; L, lip of wild type; LP, lip-like petal of the mutant type.


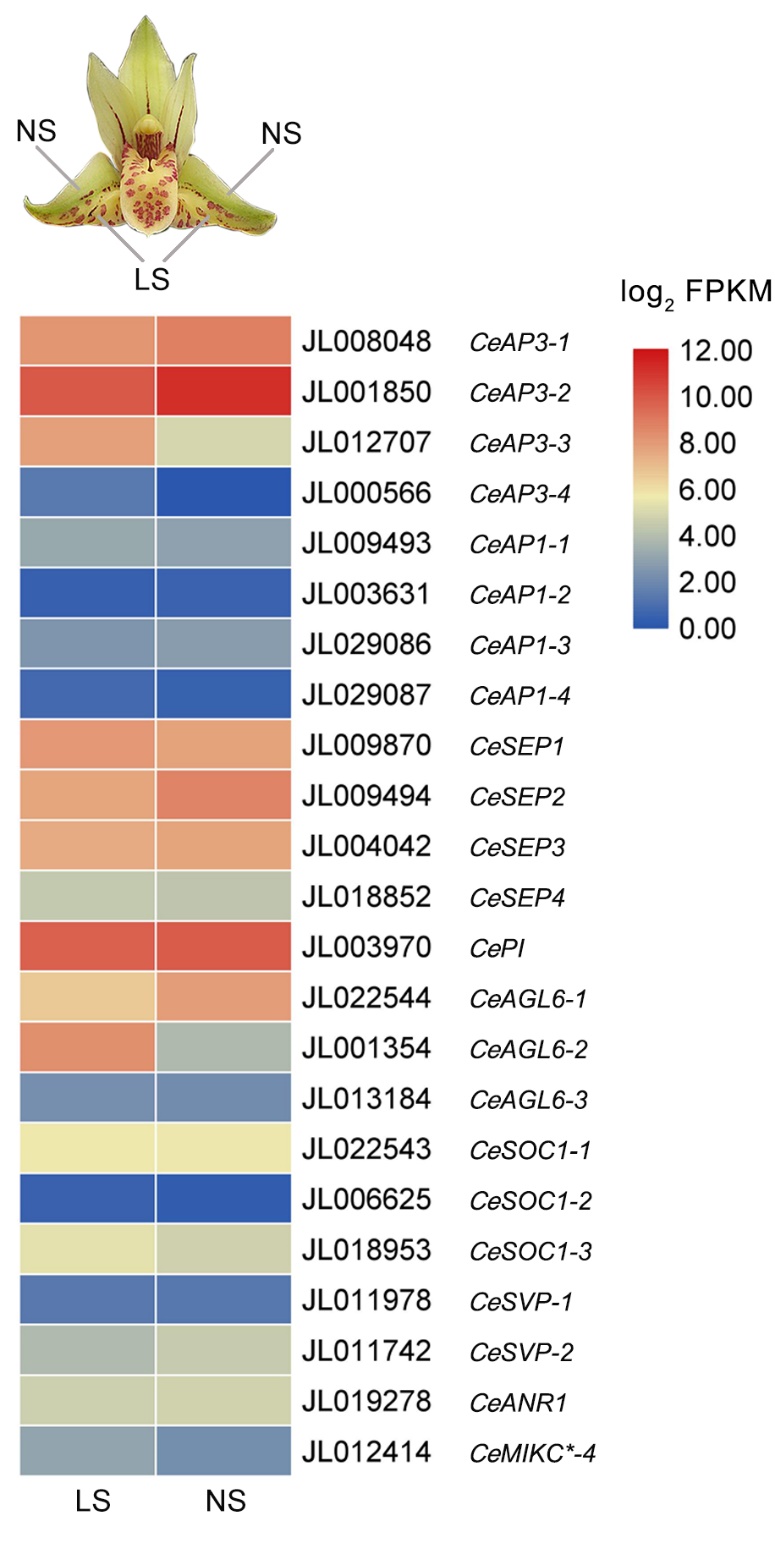


## Supplementary Fig. 20 The expression of MADS-box genes in lip-like sepal mutant of *C. ensifolium*. LS, lip-like sepal of the mutant; NS, normal sepal of the mutant.


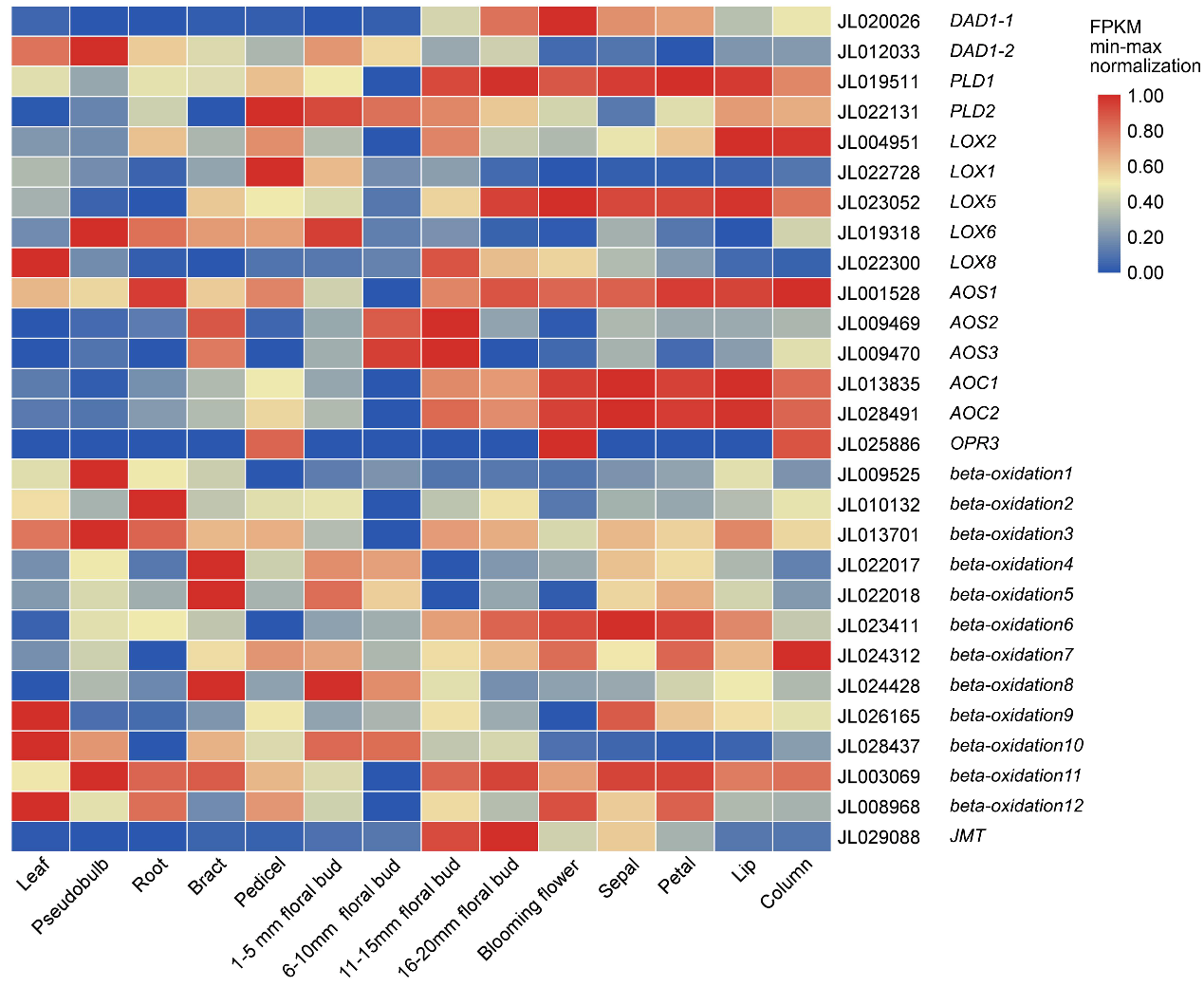


## Supplementary Fig. 21 Expression analysis of genes related to the jasmonate synthesis pathway in various floral developmental stages and different organs of *C. ensifolium*.


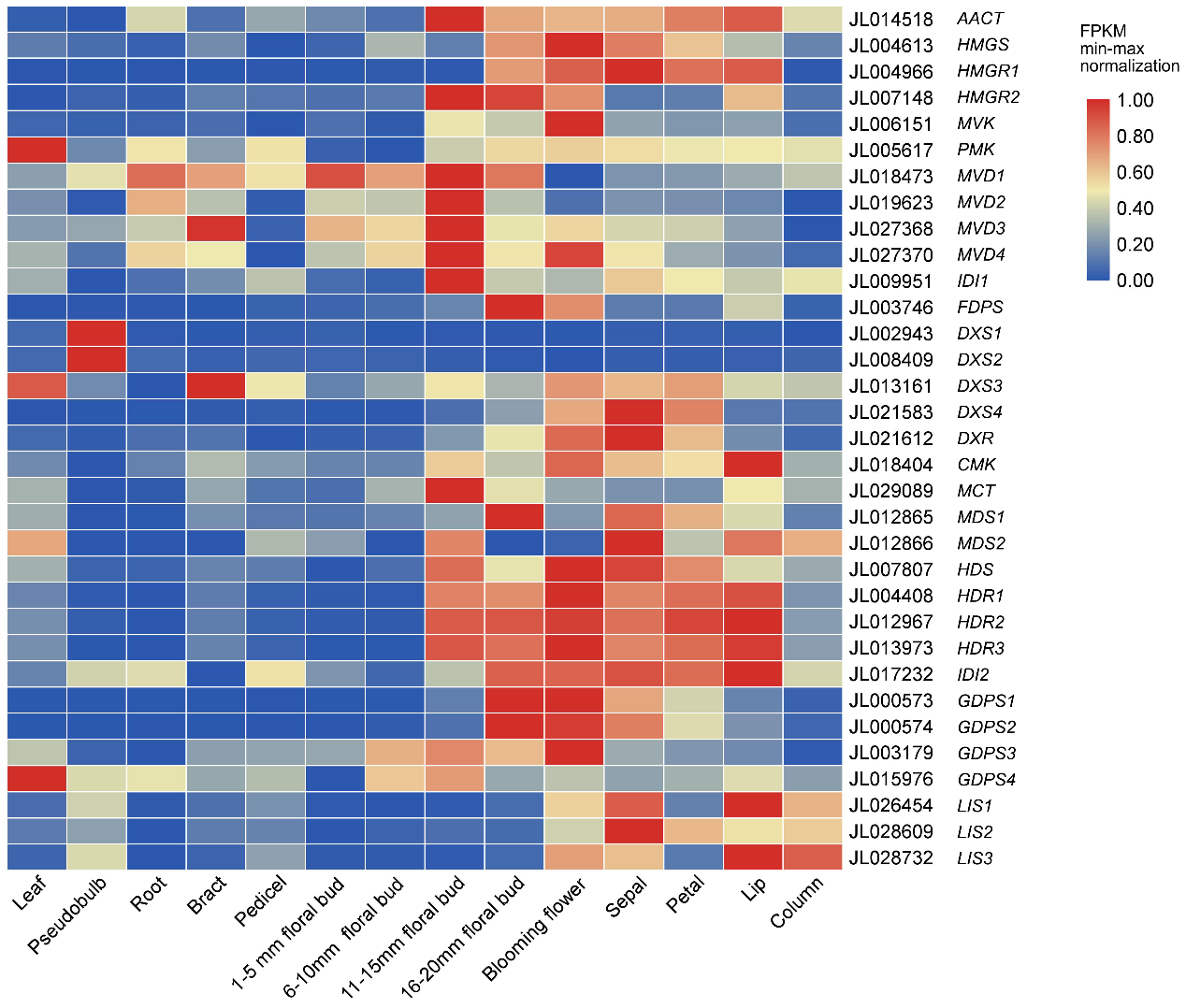


## Supplementary Fig. 22 Expression analysis of genes related to the terpenoid synthesis pathway in various floral developmental stages and different organs of *C. ensifolium*.

**
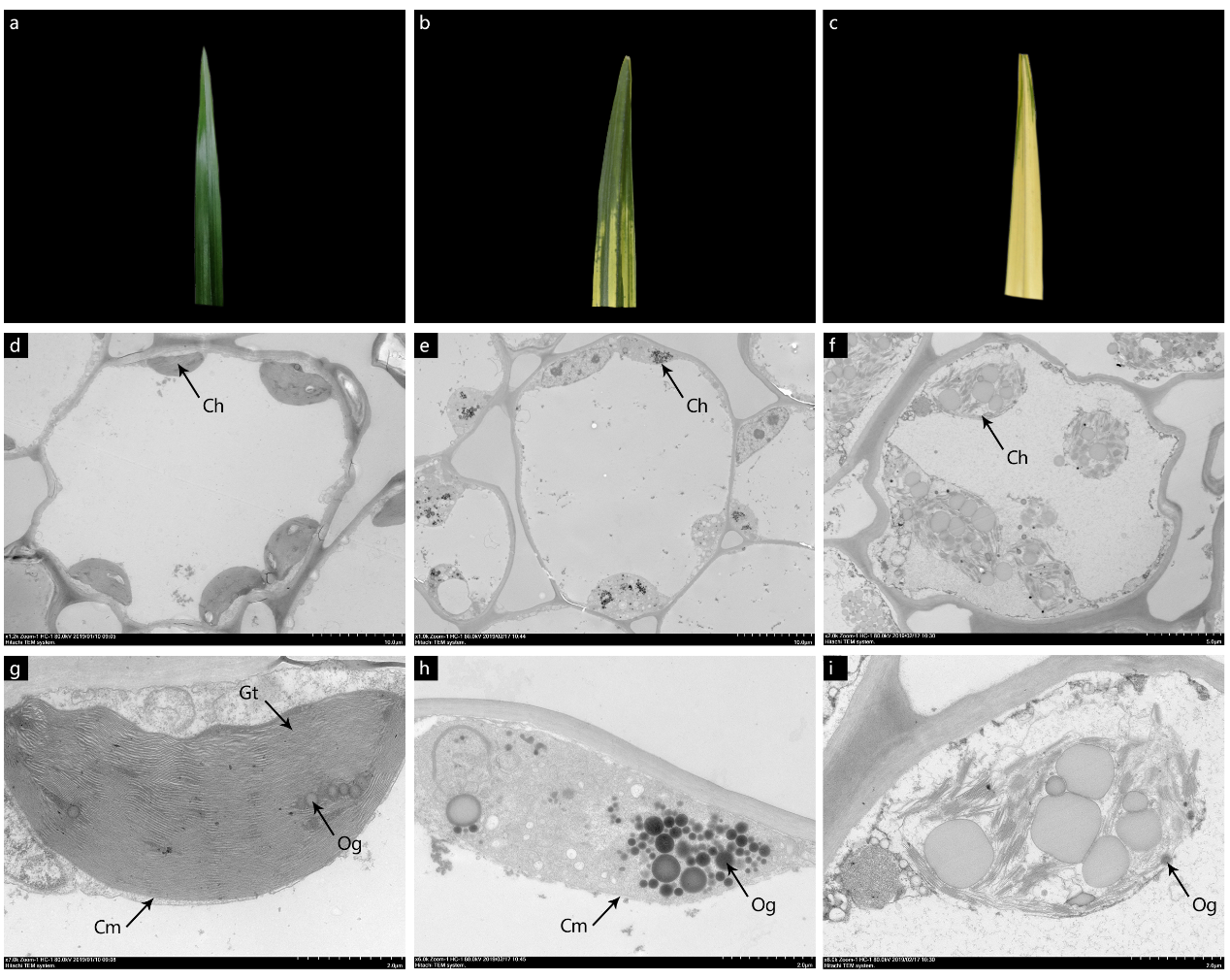
**

## Supplementary Fig. 23 Morphology and ultrastructure of leaves in different colours of ***C. ensifolium***. a. The wild-type green leaf. b. The yellow-green leaf of mutant type. c. The yellow leaf of mutant type. d. The mesophyll cell structure of wild-type green leaf. e. The mesophyll cell structure of yellow-green leaf. f. The mesophyll cell structure of yellow leaf. g. The chloroplast structure of wild-type green leaf. h. The chloroplast structure of yellow-green leaf. i. The chloroplast structure of yellow leaf.


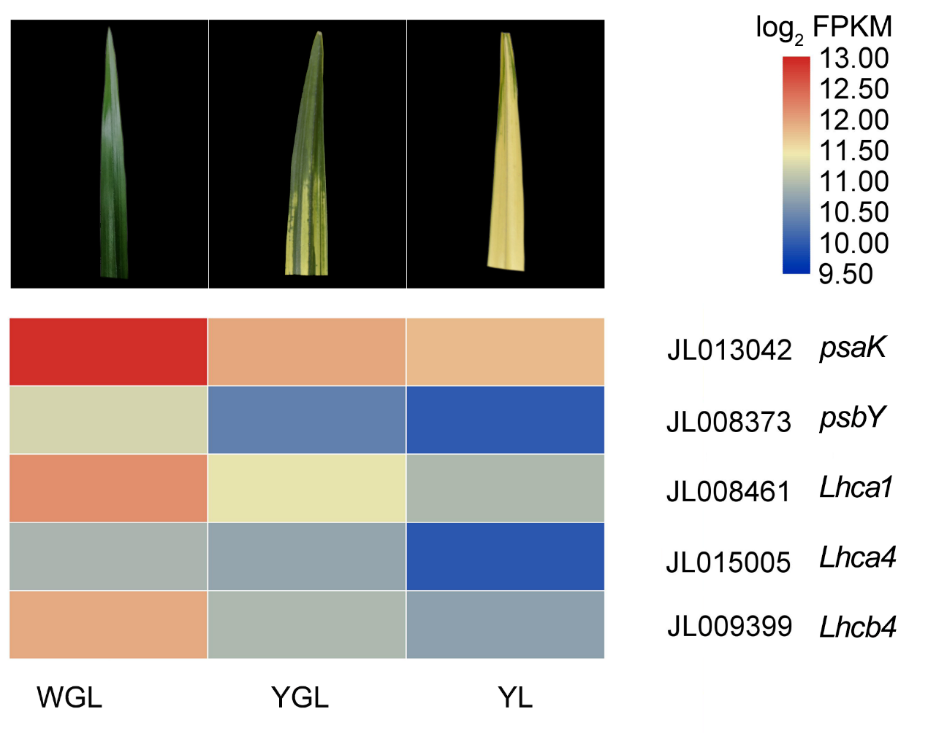


## Supplementary Fig. 24 Differentially expressed genes related to photosynthesis-antennae and photosynthesis metabolic pathways in different colour leaves of *C. ensifolium*. WGL, wild-type green leaf; YGL, yellow-green leaf of mutant type; YL, yellow leaf of mutant type.

**
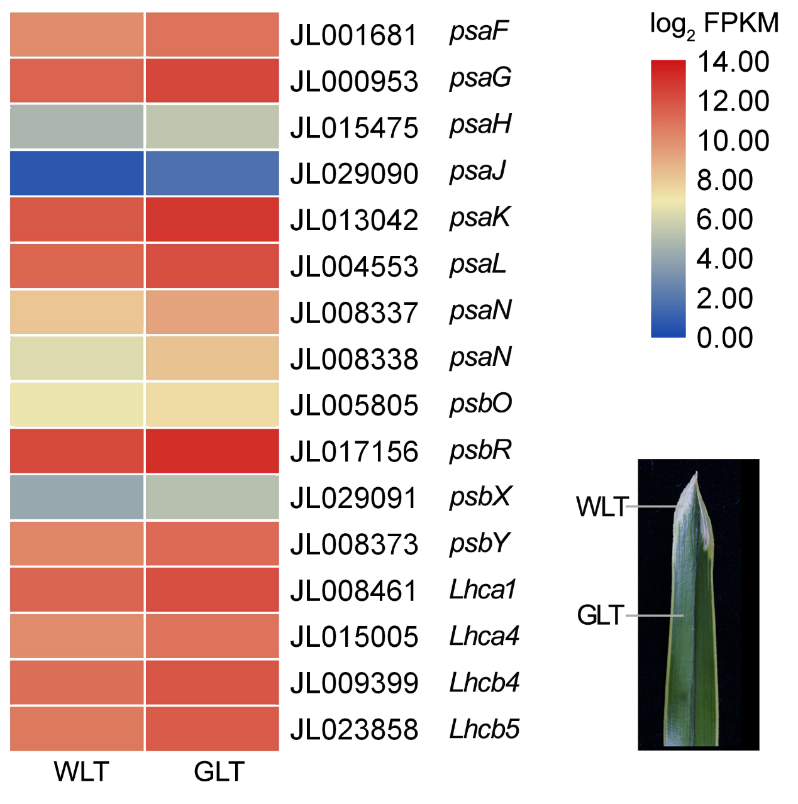
**

## Supplementary Fig. 25 Differentially expressed genes related to photosynthesis-antennae and photosynthesis metabolic pathways in green and white leaf tissues of the mutant of *C. ensifolium*. GLT, green leaf tissues; WLT, white leaf tissues.


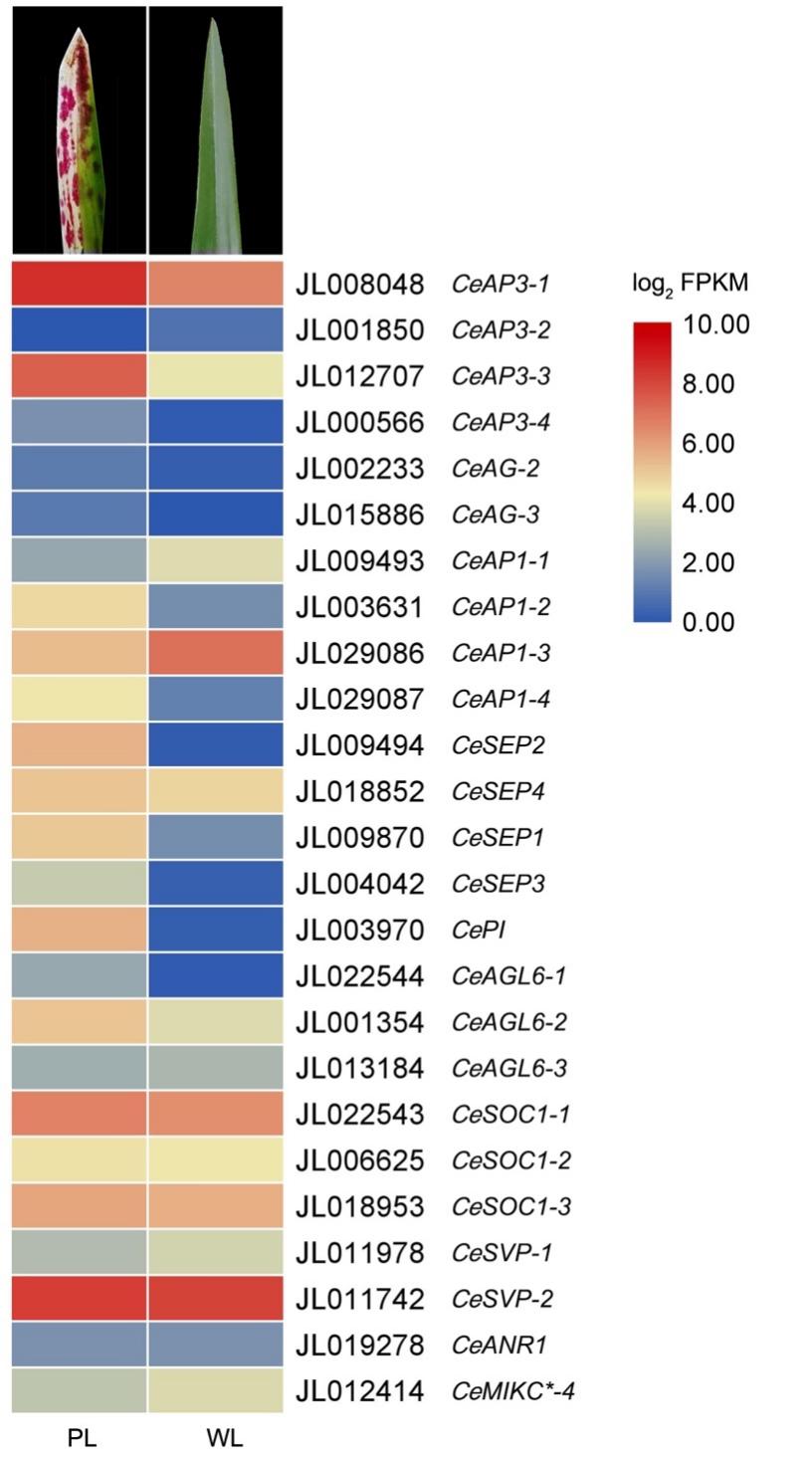


## Supplementary Fig. 26 The expression of MADS-box genes in the perianth-like leaves and the wild-type leaves of *C. ensifolium*. PL, perianth-like leaf of mutant type; WL, wild-type leaf.

# Supplementary Tables

## Supplementary Table 1. The statistics of raw sequencing data from Illumina sequencing.

| **Illumina sequence** | **Insert size (bp)** | **Read Length (bp)** | **Raw data (bp)** | **Clean data (bp)** |
| --- | --- | --- | --- | --- |
|  | 350.00 | 150.00 | 92,592,060,900 | 84,889,521,000 |

## Supplementary Table 2. *K*-mer statistics of genome sequencing results of *C. ensifolium.*

| ***K*-mer** | ***K*-mer number** | ***K*-mer Depth** | **Genome size (bp)** | **Heterozygosity (%)** |
| --- | --- | --- | --- | --- |
| 17 | 74,756,961,847 | 21 | 3,559,855,326 | 1.4% |

## Supplementary Table 3. The statistics of raw sequencing data from PacBio sequencing.

| **Cells** | **Subreads Mean Length (Kb)** | **Subreads N50 (Kb)** | **Subreads Reads Base (Gb)** |
| --- | --- | --- | --- |
| 43 | 9.9 | 15 | 351 |

Cells, the number of cells used in PacBio sequencing. Subreads Mean Length, mean length of Subreads after removal of adapter sequence. Subreads N50, N50 of Subreads after removal of adapter sequence. Subreads Read Base, total number of bases after removal of adapter sequence.

## Supplementary Table 4. Assembly statistics of the *C. ensifolium* genome

| Illumina sequencing assembly | Scaffold N50 | 1,335 bp |
| --- | --- | --- |
|  | Scaffold N90 | 180 bp |
|  | Longest Scaffold | 55,586 bp |
|  | Total Scaffold length | 1,290,514,812 bp |
| PacBio sequencing assembly | Contig N50 | 1,213,235 bp |
|  | Contig N90 | 69,543 bp |
|  | Total Contig length | 3,620,008,605 bp |
|  | Longest Contig | 9,203,368 bp |

## Supplement Table 5. BUSCO assessment of the *C. ensifolium* assembled genome.

| Type | Number | Percent (%) |
| --- | --- | --- |
| Complete BUSCOs (C) | 1,404 | 87.00 |
| Complete and single-copy BUSCOs (S) | 1,344 | 83.27 |
| Complete and duplicated BUSCOs (D) | 60 | 3.72 |
| Fragmented BUSCOs (F) | 68 | 4.21 |
| Missing BUSCOs (M) | 142 | 8.80 |
| Total BUSCO groups searched | 1,614 | - |

## Supplementary Table 6. Illumina sequence alignment statistics.

| **Total Reads** | **Mapped Reads** | **Mapped and Paired Reads** | **Mapping Ratio** | **Genome Coverage** | **Error Rate** |
| --- | --- | --- | --- | --- | --- |
| 618,850,567 | 612,676,142 | 559,680,494 | 98.87% | 91.6% | 0.024% |

Total Reads, reads statistics of filtered data. Mapped Reads, reads statistics of matched genomes; Mapped and Paired Reads, reads statistics of mapped genomes and paired.

## Supplementary Table 7. The statistical results of Hi-C assembly.

|  | **Scaffold** | | **Contig** | |
| --- | --- | --- | --- | --- |
|  | **Size (bp)** | **Number** | **Size (bp)** | **Number** |
| N10 | 228,087,709 | 2 | 4,265,413 | 65 |
| N20 | 214,310,273 | 4 | 3,025,686 | 169 |
| N30 | 213,225,312 | 5 | 2,284,889 | 308 |
| N40 | 178,440,102 | 7 | 1,692,781 | 493 |
| N50 | 154,884,659 | 9 | 1,210,201 | 747 |
| N60 | 143,505,607 | 12 | 772,116 | 1118 |
| N70 | 135,974,837 | 14 | 414,835 | 1756 |
| N80 | 120,029,532 | 17 | 178,139 | 3115 |
| N90 | 335,207 | 125 | 69,533 | 6426 |
| Total_length | 3,625,423,105 |  | 3,620,008,605 |  |
| number>=100bp | 9,021 | 19,850 |  |  |
| number>=2000bp | 9,021 | 19,850 |  |  |
| GC_rate | 33.5% | | 33.6% | |
| Anchored rate | 88.62% | | | |

## Supplementary Table 8. Chromosome length by Hi-C assembly.

| **Chromosome ID** | **Length(bp)** |
| --- | --- |
| Chr01 | 235,644,469 |
| Chr02 | 228,087,709 |
| Chr03 | 225,830,478 |
| Chr04 | 214,310,273 |
| Chr05 | 213,225,312 |
| Chr06 | 209,817,597 |
| Chr07 | 178,440,102 |
| Chr08 | 169,879,220 |
| Chr09 | 154,884,659 |
| Chr10 | 149,742,728 |
| Chr11 | 144,184,317 |
| Chr12 | 143,505,607 |
| Chr13 | 138,107,492 |
| Chr14 | 135,974,837 |
| Chr15 | 128,691,706 |
| Chr16 | 123,859,933 |
| Chr17 | 120,029,532 |
| Chr18 | 107,754,813 |
| Chr19 | 107,576,695 |
| Chr20 | 83,290,319 |
| Total | 3,212,837,798 |

## Supplementary Table 9. The prediction of gene structures of *C. ensifolium*.

| **Gene set** | | **Protein-coding gene number** | **Average gene length (bp)** | **Average CDS length (bp)** | **Average exon per gene** | **Average exon length (bp)** | **Average intron length (bp)** |
| --- | --- | --- | --- | --- | --- | --- | --- |
|  |  |  |  |  |  |  |  |
|  |  |  |  |  |  |  |  |
|  |  |  |  |  |  |  |  |
| De novo | AUGUSTUS | 49,262 | 11,527.94 | 800.61 | 3.45 | 231.90 | 4,374.15 |
|  | GlimmerHMM | 21,848 | 18,287.36 | 432.27 | 2.62 | 165.15 | 11,039.36 |
| Homolog | *P. equestris* | 63,851 | 5,234.81 | 644.79 | 2.48 | 259.57 | 3,092.94 |
|  | *D. catenatum* | 64,717 | 5,206.93 | 665.15 | 2.47 | 269.42 | 3,092.05 |
|  | *A. shenzhenica* | 51,399 | 5,774.22 | 693.82 | 2.66 | 261.07 | 3,064.91 |
|  | *G.* *elata* | 50,965 | 5,569.33 | 688.35 | 2.64 | 260.74 | 2,976.26 |
|  | *A. officinalis* | 45,630 | 5,819.04 | 669.60 | 2.73 | 244.83 | 2,968.13 |
|  | *A. comosus* | 54,318 | 6,050.95 | 658.41 | 2.61 | 251.79 | 3,339.25 |
| RNA | | 25,674 | 20,784.33 | 1,174.48 | 5.37 | 218.59 | 4,484.28 |
| CEGMA | | 442 | 22,607.26 | 1,189.45 | 8.01 | 148.43 | 3,053.77 |
| MAKER | | 47,736 | 13,807.38 | 810.83 | 3.83 | 231.42 | 4,570.61 |
| Final set | | 29,073 | 13,081.04 | 969.61 | 4.12 | 253.19 | 3,854.81 |

## Supplementary Table 10. The number of protein-coding genes supported by *de novo*, transcriptome data and homology prediction.

|  | >=20% Overlap | | >=50% Overlap | | >=80% Overlap | |
| --- | --- | --- | --- | --- | --- | --- |
|  | No. | Ratio (%) | No. | Ratio (%) | No. | Ratio (%) |
| P(single) | 5 | 0.02 | 78 | 0.27 | 1,188 | 4.09 |
| P(more) | 1 | 0 | 4 | 0.01 | 53 | 0.18 |
| H(single) | 485 | 1.67 | 641 | 2.2 | 1,325 | 4.56 |
| H(more) | 3,698 | 12.72 | 4,361 | 15 | 4,140 | 14.24 |
| R | 170 | 0.58 | 318 | 1.09 | 1,201 | 4.13 |
| P+H | 6,904 | 23.75 | 6,727 | 23.14 | 5,269 | 18.12 |
| P+R | 260 | 0.89 | 359 | 1.23 | 992 | 3.41 |
| H+R | 2,570 | 8.84 | 3,394 | 11.67 | 4,365 | 15.01 |
| P+H+R | 14,974 | 51.5 | 13,112 | 45.1 | 9,034 | 31.07 |

P, number of genes supported by the results of *de novo* prediction. H, number of genes supported by the homology prediction. R, number of genes supported by transcriptome data. Single, only one data evidence to support. More, much supporting data evidence. Overlap, ration of the final gene set to the overlap of the CDS region of various predicted results.

## Supplementary Table 11. BUSCO assessment of the *C. ensifolium*’s genome annotation.

| **Type** | **Match percentage (%)** | **Number** | **Percentage (%)** |
| --- | --- | --- | --- |
| Complete | Complete BUSCOs (C) | 1,262 | 78.19 |
|  | Complete and single-copy BUSCOs (S) | 1,213 | 75.15 |
|  | Complete and duplicated BUSCOs (D) | 49 | 3.04 |
|  | Fragmented BUSCOs (F) | 160 | 9.91 |
|  | Missing BUSCOs (M) | 192 | 11.90 |
|  | Total BUSCO groups searched | 1,614 | 100 |

## Supplementary Table 12. The statistical results of functional annotation.

|  | | **Number** | **Percent (%)** |
| --- | --- | --- | --- |
| Total | | 29,073 | 100.00 |
| Annotated | InterPro | 28,098 | 96.65 |
|  | GO | 25,721 | 88.47 |
|  | KEGG | 26,803 | 92.19 |
|  | Swissprot | 16,217 | 55.78 |
|  | TrEMBL | 26,511 | 91.19 |
|  | Nr | 26,420 | 90.87 |
| Unannotated | | 334 | 1.15 |

## Supplementary Table 13. Statistics on the annotation of non-coding RNA of the *C. ensifolium* genome.

| **Type** | | **Copy** | **Average length (bp)** | **Total length (bp)** | **% of genome** |
| --- | --- | --- | --- | --- | --- |
| miRNA | | 71 | 118.49 | 8,413 | 0.000232 |
| tRNA | | 2,018 | 74.93 | 151,207 | 0.004177 |
| rRNA | rRNA | 782 | 304.62 | 238,211 | 0.00658 |
|  | 18S | 239 | 725.36 | 173,361 | 0.004789 |
|  | 28S | 119 | 185.33 | 22,054 | 0.000609 |
|  | 5.8S | 25 | 162.08 | 4,052 | 0.000112 |
|  | 5S | 399 | 97.10 | 38,744 | 0.00107 |
|  | snRNA | 139 | 127.75 | 17,757 | 0.000491 |
| snRNA | CD-box | 49 | 111.59 | 5,468 | 0.000151 |
|  | HACA-box | 0 | 0 | 0 | 0 |
|  | splicing | 90 | 136.54 | 12,289 | 0.000339 |
|  | scaRNA | 0 | 0 | 0 | 0 |

## Supplementary Table 14. The statistical results of repeat sequences.

| **Type** | **Repeat Size (bp)** | **% of genome** |
| --- | --- | --- |
| TRF | 741,112,536 | 20.47 |
| RepeatMasker | 474,329,288 | 13.10 |
| RepeatProteinMask | 425,504,246 | 11.75 |
| De novo | 2,735,577,553 | 75.57 |
| Total | 2,916,940,149 | 80.58 |

## Supplementary Table 15. Statistics of repeat sequences in *C. ensifolium*.

|  | **RepBase TEs** | | **TE Proteins** | | **De novo** | | **Combined TEs** | |
| --- | --- | --- | --- | --- | --- | --- | --- | --- |
| **Length**  **(bp)** | **% in Genome** | **Length (bp)** | **% in Genome** | **Length**  **(bp)** | **% in Genome** | **Length**  **(bp)** | **% in Genome** |  |
| DNA | 53,272,938 | 1.47 | 13,668,451 | 0.38 | 511,217,905 | 14.12 | 545,903,675 | 15.08 |
| LINE | 144,916,716 | 4.00 | 112,810,303 | 3.12 | 571,068,572 | 15.78 | 595,821,116 | 16.46 |
| SINE | 32,290 | 0.00 | 0 | 0.00 | 2,247,809 | 0.06 | 2,279,928 | 0.06 |
| LTR | 254,899,618 | 7.04 | 299,103,554 | 8.26 | 1,733,913,470 | 47.90 | 1,773,085,406 | 48.98 |
| Other | 33,697 | 0.00 | 0 | 0.00 | 0 | 0.00 | 33,697 | 0.00 |
| Unknown | 241,815 | 0.01 | 0 | 0.00 | 154,373,990 | 4.26 | 154,613,982 | 4.27 |
| Total | 474,329,288 | 13.10 | 425,504,246 | 11.75 | 2,544,711,782 | 70.30 | 2,597,763,066 | 71.76 |

LINE, long interspersed nuclear element. SINE, short interspersed element. LTR, long terminal repeat. De novo+RepBase denotes transposable elements identified by RepeatMasker (<http://www.repeatmasker.org>) with default options after RepeatModeler/RepeatScout/Piler/LTR_finder software use with RepBase database prediction. TE proteins, were transposable elements identified in the genome through the annotation of Repeat ProteinMask software using the RepBase database. Combined TEs, involved a combination of the above two methods. Unknown, repeat sequences could not be clustered by Repeat Masker.

## Supplementary Table 16. Statistical results of clustered gene families.

| **Species** | **Genes** | **Unclustered genes** | **Clustered genes** | **Families** | **Unique families** | **Unique families**  **genes** | **Common families** | **Common**  **families**  **genes** | **Single copy families** | **Average**  **genes**  **per**  **family** |
| --- | --- | --- | --- | --- | --- | --- | --- | --- | --- | --- |
| *A. comosus* | 27,024 | 5,852 | 21,172 | 12,932 | 786 | 3,053 | 3,915 | 7,025 | 277 | 1.637 |
| *A. officinalis* | 27,375 | 8,092 | 19,283 | 11,828 | 826 | 3,580 | 3,915 | 6,848 | 277 | 1.63 |
| *A. shenzhenica* | 20,560 | 4,223 | 16,337 | 11,794 | 325 | 1,236 | 3,915 | 6,078 | 277 | 1.385 |
| *A. thaliana* | 26,637 | 3,728 | 22,909 | 12,434 | 782 | 3,263 | 3,915 | 8,046 | 277 | 1.842 |
| *A. trichopoda* | 25,933 | 7,622 | 18,311 | 12,084 | 938 | 4,034 | 3,915 | 5,643 | 277 | 1.515 |
| *B. distachyon* | 26,415 | 3,639 | 22,776 | 14,753 | 376 | 1,078 | 3,915 | 7,766 | 277 | 1.544 |
| *D. catenatum* | 26,791 | 5,660 | 21,131 | 13,857 | 661 | 2,648 | 3,915 | 6,721 | 277 | 1.525 |
| *G. elata* | 18,019 | 3,848 | 14,171 | 10,564 | 217 | 665 | 3,915 | 5,639 | 277 | 1.341 |
| *C. ensifolium* | 29,073 | 5,940 | 23,133 | 14,267 | 786 | 2,104 | 3,915 | 7,138 | 277 | 1.621 |
| *M. acuminata* | 34,241 | 8,624 | 25,617 | 12,523 | 490 | 1,241 | 3,915 | 10,872 | 277 | 2.046 |
| *O. sativa* | 35,402 | 11,052 | 24,350 | 15,760 | 910 | 2,392 | 3,915 | 7,636 | 277 | 1.545 |
| *P. aphrodite* | 28,910 | 4,012 | 24,898 | 13,790 | 407 | 2,083 | 3,915 | 6,657 | 277 | 1.806 |
| *P. dactylifera* | 23,890 | 6,080 | 17,810 | 10,807 | 367 | 1,075 | 3,915 | 7,325 | 277 | 1.648 |
| *P. equestris* | 26,471 | 7,141 | 19,330 | 14,164 | 454 | 1,122 | 3,915 | 6,491 | 277 | 1.365 |
| *P. trichocarpa* | 40,984 | 7,633 | 33,351 | 14,052 | 1,241 | 3,767 | 3,915 | 11,441 | 277 | 2.373 |
| *S. bicolor* | 27,160 | 3,692 | 23,468 | 15,110 | 332 | 915 | 3,915 | 7,938 | 277 | 1.553 |
| *S. polyrhiza* | 18,357 | 4,987 | 13,370 | 10,000 | 239 | 713 | 3,915 | 5,604 | 277 | 1.337 |
| *V. vinifera* | 25,328 | 5,964 | 19,364 | 12,547 | 590 | 1,741 | 3,915 | 7,066 | 277 | 1.543 |

## Supplementary Table 17. GO enrichment of significantly expanded (*p* <0.05) gene families of the *C. ensifolium* genome.

| **GO ID** | **GO Term** | **GO**  **Class** | **P value** | **Adjusted P value** | **x1** | **x2** | **n** | **N** | **GO**  **level** |
| --- | --- | --- | --- | --- | --- | --- | --- | --- | --- |
| GO:0003824 | catalytic activity | MF | 8.27E-54 | 1.16E-50 | 723 | 10586 | 1261 | 29073 | 2 |
| GO:0008236 | serine-type peptidase activity | MF | 5.5E-36 | 7.73E-33 | 85 | 387 | 1261 | 29073 | 5 |
| GO:0004252 | serine-type endopeptidase activity | MF | 8.68E-35 | 1.22E-31 | 68 | 253 | 1261 | 29073 | 6 |
| GO:0004175 | endopeptidase activity | MF | 1.83E-32 | 2.57E-29 | 114 | 742 | 1261 | 29073 | 6 |
| GO:0008152 | metabolic process | BP | 7.33E-31 | 1.03E-27 | 755 | 12809 | 1261 | 29073 | 2 |
| GO:0055085 | transmembrane transport | BP | 1.31E-29 | 1.85E-26 | 145 | 1197 | 1261 | 29073 | 4 |
| GO:0016485 | protein processing | BP | 8.32E-29 | 1.17E-25 | 161 | 1442 | 1261 | 29073 | 6 |
| GO:0070011 | peptidase activity, acting on L-amino acid peptides | MF | 3.09E-26 | 4.35E-23 | 136 | 1164 | 1261 | 29073 | 5 |
| GO:0006508 | proteolysis | BP | 1.07E-25 | 1.5E-22 | 136 | 1179 | 1261 | 29073 | 7 |
| GO:0042802 | identical protein binding | MF | 1.05E-24 | 1.48E-21 | 31 | 67 | 1261 | 29073 | 4 |
| GO:0043086 | negative regulation of catalytic activity | BP | 2.8E-23 | 3.94E-20 | 35 | 97 | 1261 | 29073 | 5 |
| GO:0055114 | oxidation-reduction process | BP | 4.37E-22 | 6.15E-19 | 239 | 2952 | 1261 | 29073 | 4 |
| GO:0044710 | single-organism metabolic process | BP | 4.62E-22 | 6.49E-19 | 358 | 5133 | 1261 | 29073 | 3 |
| GO:0004970 | ionotropic glutamate receptor activity | MF | 1.12E-18 | 1.58E-15 | 14 | 15 | 1261 | 29073 | 6 |
| GO:0005215 | transporter activity | MF | 3.67E-18 | 5.16E-15 | 321 | 4696 | 1261 | 29073 | 2 |
| GO:0016787 | hydrolase activity | MF | 7.02E-18 | 9.86E-15 | 288 | 4089 | 1261 | 29073 | 3 |
| GO:0016491 | oxidoreductase activity | MF | 3.22E-17 | 4.53E-14 | 231 | 3082 | 1261 | 29073 | 3 |
| GO:0016887 | ATPase activity | MF | 4.6E-16 | 6.47E-13 | 80 | 677 | 1261 | 29073 | 8 |
| GO:0005234 | extracellular-glutamate-gated ion channel activity | MF | 7.38E-16 | 1.04E-12 | 14 | 19 | 1261 | 29073 | 10 |
| GO:0019538 | protein metabolic process | BP | 1.43E-15 | 2.01E-12 | 265 | 3818 | 1261 | 29073 | 4 |
| GO:0003333 | amino acid transmembrane transport | BP | 2.12E-15 | 2.98E-12 | 18 | 37 | 1261 | 29073 | 6 |
| GO:0016740 | transferase activity | MF | 2.14E-15 | 3.01E-12 | 232 | 3216 | 1261 | 29073 | 3 |
| GO:0043167 | ion binding | MF | 8.39E-15 | 1.18E-11 | 407 | 6688 | 1261 | 29073 | 3 |
| GO:0004497 | monooxygenase activity | MF | 2.77E-14 | 3.89E-11 | 81 | 743 | 1261 | 29073 | 4 |
| GO:0016758 | transferase activity, transferring hexosyl groups | MF | 4.01E-14 | 5.64E-11 | 59 | 450 | 1261 | 29073 | 5 |
| GO:0046906 | tetrapyrrole binding | MF | 4.63E-14 | 6.5E-11 | 106 | 1124 | 1261 | 29073 | 4 |
| GO:0051234 | establishment of localization | BP | 1.6E-13 | 2.25E-10 | 362 | 5891 | 1261 | 29073 | 2 |
| GO:0006810 | transport | BP | 2.05E-13 | 2.89E-10 | 361 | 5881 | 1261 | 29073 | 3 |
| GO:0071805 | potassium ion transmembrane transport | BP | 2.32E-13 | 3.27E-10 | 31 | 148 | 1261 | 29073 | 5 |
| GO:0016020 | membrane | CC | 2.54E-13 | 3.57E-10 | 619 | 11406 | 1261 | 29073 | 2 |
| GO:0034220 | ion transmembrane transport | BP | 1.4E-12 | 1.97E-09 | 70 | 640 | 1261 | 29073 | 5 |
| GO:0015979 | photosynthesis | BP | 1.41E-12 | 1.98E-09 | 34 | 188 | 1261 | 29073 | 4 |
| GO:0051179 | localization | BP | 1.91E-12 | 2.68E-09 | 368 | 6119 | 1261 | 29073 | 2 |
| GO:0016705 | oxidoreductase activity, acting on paired donors, with incorporation or reduction of molecular oxygen | MF | 2.53E-12 | 3.56E-09 | 100 | 1102 | 1261 | 29073 | 4 |
| GO:0022857 | transmembrane transporter activity | MF | 3.5E-12 | 4.93E-09 | 264 | 4064 | 1261 | 29073 | 3 |
| GO:0016829 | lyase activity | MF | 4.48E-12 | 6.3E-09 | 47 | 346 | 1261 | 29073 | 3 |
| GO:0016757 | transferase activity, transferring glycosyl groups | MF | 1.63E-11 | 2.29E-08 | 63 | 574 | 1261 | 29073 | 4 |
| GO:0016984 | ribulose-bisphosphate carboxylase activity | MF | 1.94E-11 | 2.73E-08 | 10 | 14 | 1261 | 29073 | 6 |
| GO:0004190 | aspartic-type endopeptidase activity | MF | 3.25E-11 | 4.57E-08 | 39 | 267 | 1261 | 29073 | 7 |
| GO:0015405 | P-P-bond-hydrolysis-driven transmembrane transporter activity | MF | 3.43E-11 | 4.83E-08 | 43 | 316 | 1261 | 29073 | 6 |
| GO:0009055 | electron carrier activity | MF | 3.51E-11 | 4.93E-08 | 71 | 701 | 1261 | 29073 | 2 |
| GO:0009767 | photosynthetic electron transport chain | BP | 4.14E-11 | 5.82E-08 | 16 | 46 | 1261 | 29073 | 5 |
| GO:0071705 | nitrogen compound transport | BP | 4.4E-11 | 6.19E-08 | 58 | 517 | 1261 | 29073 | 4 |
| GO:0016746 | transferase activity, transferring acyl groups | MF | 4.88E-11 | 6.86E-08 | 42 | 307 | 1261 | 29073 | 4 |
| GO:0016307 | phosphatidylinositol phosphate kinase activity | MF | 5.37E-11 | 7.55E-08 | 11 | 19 | 1261 | 29073 | 6 |
| GO:0020037 | heme binding | MF | 5.62E-11 | 7.9E-08 | 96 | 1098 | 1261 | 29073 | 5 |
| GO:0016820 | hydrolase activity, acting on acid anhydrides, catalyzing transmembrane movement of substances | MF | 6.93E-11 | 9.74E-08 | 41 | 298 | 1261 | 29073 | 4 |
| GO:0005488 | binding | MF | 8.45E-11 | 1.19E-07 | 687 | 13274 | 1261 | 29073 | 2 |
| GO:0044699 | single-organism process | BP | 1.04E-10 | 1.47E-07 | 795 | 15801 | 1261 | 29073 | 2 |
| GO:0030674 | protein binding, bridging | MF | 1.06E-10 | 1.49E-07 | 8 | 9 | 1261 | 29073 | 4 |
| GO:0051015 | actin filament binding | MF | 1.06E-10 | 1.49E-07 | 8 | 9 | 1261 | 29073 | 5 |
| GO:0005664 | nuclear origin of replication recognition complex | CC | 1.06E-10 | 1.49E-07 | 8 | 9 | 1261 | 29073 | 5 |
| GO:0016702 | oxidoreductase activity, acting on single donors with incorporation of molecular oxygen, incorporation of two atoms of oxygen | MF | 1.8E-10 | 2.53E-07 | 13 | 31 | 1261 | 29073 | 5 |
| GO:0005524 | ATP binding | MF | 2.24E-10 | 3.15E-07 | 183 | 2666 | 1261 | 29073 | 6 |
| GO:0034357 | photosynthetic membrane | CC | 2.49E-10 | 3.51E-07 | 28 | 159 | 1261 | 29073 | 3 |
| GO:0010333 | terpene synthase activity | MF | 4.04E-10 | 5.68E-07 | 17 | 60 | 1261 | 29073 | 6 |
| GO:0000287 | magnesium ion binding | MF | 4.07E-10 | 5.72E-07 | 30 | 184 | 1261 | 29073 | 6 |
| GO:0044765 | single-organism transport | BP | 9.67E-10 | 1.36E-06 | 295 | 4913 | 1261 | 29073 | 3 |
| GO:0042626 | ATPase activity, coupled to transmembrane movement of substances | MF | 1.15E-09 | 1.61E-06 | 38 | 288 | 1261 | 29073 | 5 |
| GO:0015977 | carbon fixation | BP | 1.47E-09 | 2.07E-06 | 10 | 19 | 1261 | 29073 | 4 |
| GO:0035639 | purine ribonucleoside triphosphate binding | MF | 1.73E-09 | 2.43E-06 | 203 | 3118 | 1261 | 29073 | 5 |
| GO:0000166 | nucleotide binding | MF | 2.53E-09 | 3.55E-06 | 227 | 3599 | 1261 | 29073 | 4 |
| GO:0032550 | purine ribonucleoside binding | MF | 5.24E-09 | 7.37E-06 | 203 | 3161 | 1261 | 29073 | 6 |
| GO:0032555 | purine ribonucleotide binding | MF | 5.94E-09 | 8.35E-06 | 203 | 3166 | 1261 | 29073 | 5 |
| GO:0022900 | electron transport chain | BP | 6.84E-09 | 9.61E-06 | 21 | 108 | 1261 | 29073 | 4 |
| GO:0004523 | RNA-DNA hybrid ribonuclease activity | MF | 9.47E-09 | 1.33E-05 | 7 | 9 | 1261 | 29073 | 9 |
| GO:0043168 | anion binding | MF | 1.51E-08 | 2.12E-05 | 223 | 3596 | 1261 | 29073 | 4 |
| GO:1901363 | heterocyclic compound binding | MF | 1.77E-08 | 2.49E-05 | 447 | 8263 | 1261 | 29073 | 3 |
| GO:0017004 | cytochrome complex assembly | BP | 1.9E-08 | 2.68E-05 | 34 | 266 | 1261 | 29073 | 7 |
| GO:0017076 | purine nucleotide binding | MF | 2.01E-08 | 2.82E-05 | 204 | 3235 | 1261 | 29073 | 5 |
| GO:0097159 | organic cyclic compound binding | MF | 2.05E-08 | 2.88E-05 | 447 | 8272 | 1261 | 29073 | 3 |
| GO:0016539 | intein-mediated protein splicing | BP | 3.65E-08 | 5.13E-05 | 34 | 273 | 1261 | 29073 | 8 |
| GO:0009521 | photosystem | CC | 5.7E-08 | 8.02E-05 | 22 | 132 | 1261 | 29073 | 3 |
| GO:1901678 | iron coordination entity transport | BP | 6.33E-08 | 8.9E-05 | 33 | 266 | 1261 | 29073 | 4 |
| GO:0016835 | carbon-oxygen lyase activity | MF | 6.48E-08 | 9.11E-05 | 23 | 144 | 1261 | 29073 | 4 |
| GO:0046488 | phosphatidylinositol metabolic process | BP | 7.18E-08 | 0.000101 | 15 | 64 | 1261 | 29073 | 7 |
| GO:0046486 | glycerolipid metabolic process | BP | 7.29E-08 | 0.000102 | 16 | 73 | 1261 | 29073 | 5 |
| GO:0016817 | hydrolase activity, acting on acid anhydrides | MF | 9.41E-08 | 0.000132 | 102 | 1377 | 1261 | 29073 | 4 |
| GO:0000910 | cytokinesis | BP | 1.16E-07 | 0.000162 | 8 | 16 | 1261 | 29073 | 5 |
| GO:0006865 | amino acid transport | BP | 1.33E-07 | 0.000187 | 25 | 173 | 1261 | 29073 | 5 |
| GO:0033178 | proton-transporting two-sector ATPase complex, catalytic domain | CC | 1.39E-07 | 0.000195 | 12 | 42 | 1261 | 29073 | 3 |
| GO:0003998 | acylphosphatase activity | MF | 1.71E-07 | 0.00024 | 6 | 8 | 1261 | 29073 | 6 |
| GO:0015232 | heme transporter activity | MF | 1.74E-07 | 0.000245 | 31 | 251 | 1261 | 29073 | 4 |
| GO:0015886 | heme transport | BP | 1.74E-07 | 0.000245 | 31 | 251 | 1261 | 29073 | 5 |
| GO:0016168 | chlorophyll binding | MF | 2.14E-07 | 0.000301 | 10 | 29 | 1261 | 29073 | 5 |
| GO:0010467 | gene expression | BP | 2.35E-07 | 0.000331 | 282 | 4924 | 1261 | 29073 | 5 |
| GO:0015991 | ATP hydrolysis coupled proton transport | BP | 2.45E-07 | 0.000344 | 12 | 44 | 1261 | 29073 | 8 |
| GO:0006629 | lipid metabolic process | BP | 2.58E-07 | 0.000363 | 45 | 454 | 1261 | 29073 | 4 |
| GO:0016818 | hydrolase activity, acting on acid anhydrides, in phosphorus-containing anhydrides | MF | 3.89E-07 | 0.000547 | 99 | 1365 | 1261 | 29073 | 5 |
| GO:0016830 | carbon-carbon lyase activity | MF | 5.5E-07 | 0.000773 | 21 | 138 | 1261 | 29073 | 4 |
| GO:0015171 | amino acid transmembrane transporter activity | MF | 5.64E-07 | 0.000793 | 24 | 174 | 1261 | 29073 | 7 |
| GO:0070008 | serine-type exopeptidase activity | MF | 6.81E-07 | 0.000958 | 19 | 117 | 1261 | 29073 | 6 |
| GO:0044255 | cellular lipid metabolic process | BP | 8.46E-07 | 0.00119 | 30 | 256 | 1261 | 29073 | 4 |
| GO:0045735 | nutrient reservoir activity | MF | 1.01E-06 | 0.001427 | 19 | 120 | 1261 | 29073 | 2 |
| GO:0004185 | serine-type carboxypeptidase activity | MF | 1.19E-06 | 0.001667 | 18 | 110 | 1261 | 29073 | 7 |
| GO:0022891 | substrate-specific transmembrane transporter activity | MF | 1.47E-06 | 0.002069 | 226 | 3876 | 1261 | 29073 | 4 |
| GO:0004180 | carboxypeptidase activity | MF | 1.49E-06 | 0.002096 | 19 | 123 | 1261 | 29073 | 7 |
| GO:0005337 | nucleoside transmembrane transporter activity | MF | 1.75E-06 | 0.002456 | 30 | 265 | 1261 | 29073 | 4 |
| GO:0004478 | methionine adenosyltransferase activity | MF | 2.97E-06 | 0.00418 | 5 | 7 | 1261 | 29073 | 5 |
| GO:0006556 | S-adenosylmethionine biosynthetic process | BP | 2.97E-06 | 0.00418 | 5 | 7 | 1261 | 29073 | 6 |
| GO:0017111 | nucleoside-triphosphatase activity | MF | 3.49E-06 | 0.00491 | 93 | 1325 | 1261 | 29073 | 7 |
| GO:0006091 | generation of precursor metabolites and energy | BP | 5.77E-06 | 0.008109 | 30 | 281 | 1261 | 29073 | 4 |
| GO:0022892 | substrate-specific transporter activity | MF | 7.96E-06 | 0.011188 | 228 | 4011 | 1261 | 29073 | 3 |
| GO:0005506 | iron ion binding | MF | 9.54E-06 | 0.013407 | 63 | 821 | 1261 | 29073 | 7 |
| GO:0006813 | potassium ion transport | BP | 1.38E-05 | 0.019392 | 100 | 1502 | 1261 | 29073 | 7 |
| GO:0004332 | fructose-bisphosphate aldolase activity | MF | 1.46E-05 | 0.020573 | 6 | 14 | 1261 | 29073 | 6 |
| GO:0046943 | carboxylic acid transmembrane transporter activity | MF | 1.46E-05 | 0.020589 | 25 | 223 | 1261 | 29073 | 6 |
| GO:0051213 | dioxygenase activity | MF | 1.47E-05 | 0.020685 | 21 | 169 | 1261 | 29073 | 4 |
| GO:0046942 | carboxylic acid transport | BP | 1.7E-05 | 0.023882 | 26 | 239 | 1261 | 29073 | 5 |
| GO:0004089 | carbonate dehydratase activity | MF | 1.7E-05 | 0.02391 | 4 | 5 | 1261 | 29073 | 6 |
| GO:0050661 | NADP binding | MF | 1.71E-05 | 0.02403 | 12 | 64 | 1261 | 29073 | 5 |
| GO:0004499 | N, N-dimethylaniline monooxygenase activity | MF | 2.11E-05 | 0.029621 | 40 | 455 | 1261 | 29073 | 6 |
| GO:0071704 | organic substance metabolic process | BP | 2.6E-05 | 0.036492 | 494 | 9829 | 1261 | 29073 | 3 |
| GO:0016884 | carbon-nitrogen ligase activity, with glutamine as amido-N-donor | MF | 2.76E-05 | 0.038742 | 9 | 38 | 1261 | 29073 | 5 |
| GO:0000155 | phosphorelay sensor kinase activity | MF | 3.05E-05 | 0.042908 | 8 | 30 | 1261 | 29073 | 4 |
| GO:0015672 | monovalent inorganic cation transport | BP | 3.12E-05 | 0.04383 | 126 | 2033 | 1261 | 29073 | 6 |
| GO:0003899 | DNA-directed RNA polymerase activity | MF | 3.36E-05 | 0.047238 | 16 | 114 | 1261 | 29073 | 7 |
| GO:0016831 | carboxy-lyase activity | MF | 3.36E-05 | 0.047268 | 15 | 102 | 1261 | 29073 | 5 |

## Supplementary Table 18. KEGG pathway enrichment of significantly expanded (*p* <0.01) gene families of the *C. ensifolium* genome.

| Map ID | Map Title | P value | Adjusted P value | x | y | n | N |
| --- | --- | --- | --- | --- | --- | --- | --- |
| map02010 | ABC transporters | 3.92E-25 | 1.92E-23 | 28 | 51 | 1261 | 29073 |
| map00195 | Photosynthesis | 1.30E-14 | 6.39E-13 | 37 | 190 | 1261 | 29073 |
| map00562 | Inositol phosphate metabolism | 9.59E-14 | 4.70E-12 | 23 | 77 | 1261 | 29073 |
| map00591 | Linoleic acid metabolism | 2.66E-13 | 1.30E-11 | 13 | 21 | 1261 | 29073 |
| map04070 | Phosphatidylinositol signaling system | 1.67E-12 | 8.19E-11 | 23 | 87 | 1261 | 29073 |
| map00941 | Flavonoid biosynthesis | 1.74E-10 | 8.55E-09 | 16 | 50 | 1261 | 29073 |
| map01100 | Metabolic pathways | 1.85E-10 | 9.07E-09 | 168 | 2382 | 1261 | 29073 |
| map00062 | Fatty acid elongation | 1.86E-10 | 9.11E-09 | 14 | 37 | 1261 | 29073 |
| map04712 | Circadian rhythm-plant | 3.38E-10 | 1.66E-08 | 16 | 52 | 1261 | 29073 |
| map03020 | RNA polymerase | 9.25E-07 | 4.53E-05 | 16 | 87 | 1261 | 29073 |
| map01110 | Biosynthesis of secondary metabolites | 3.22E-06 | 0.000158 | 84 | 1160 | 1261 | 29073 |
| map00190 | Oxidative phosphorylation | 9.30E-06 | 0.000456 | 27 | 245 | 1261 | 29073 |
| map00710 | Carbon fixation in photosynthetic organisms | 6.52E-05 | 0.003193 | 17 | 133 | 1261 | 29073 |
| map00900 | Terpenoid backbone biosynthesis | 0.000472 | 0.023151 | 11 | 77 | 1261 | 29073 |
| map00100 | Steroid biosynthesis | 0.000888 | 0.043532 | 6 | 27 | 1261 | 29073 |

## Supplementary Table 19. GO enrichment of significantly contracted (*p* <0.05) gene families of the *C. ensifolium* genome.

| GO ID | GO Term | GO Class | P value | Adjusted P value | x1 | x2 | n | N | GO  level |
| --- | --- | --- | --- | --- | --- | --- | --- | --- | --- |
| GO:0016459 | myosin complex | CC | 4.37E-15 | 9.83E-13 | 5 | 10 | 15 | 29073 | 4 |
| GO:0003774 | motor activity | MF | 7.65E-08 | 1.72E-05 | 5 | 226 | 15 | 29073 | 8 |
| GO:0005524 | ATP binding | MF | 1.76E-05 | 0.003969 | 8 | 2666 | 15 | 29073 | 6 |
| GO:0043167 | ion binding | MF | 5.04E-05 | 0.011348 | 11 | 6688 | 15 | 29073 | 3 |
| GO:0005515 | protein binding | MF | 0.000104 | 0.023402 | 9 | 4502 | 15 | 29073 | 3 |

## Supplementary Table 20. GO enrichment of unique gene families in the *C. ensifolium* genome.

| GO ID | GO Term | GO Class | P value | Adjusted P value | x1 | x2 | n | N | GO level |
| --- | --- | --- | --- | --- | --- | --- | --- | --- | --- |
| GO:0008234 | cysteine-type peptidase activity | MF | 9.93E-11 | 1.89E-07 | 46 | 224 | 2104 | 29073 | 6 |
| GO:0008375 | acetylglucosaminyltransferase activity | MF | 3.98E-08 | 7.59E-05 | 20 | 68 | 2104 | 29073 | 6 |
| GO:0009496 | plastoquinol-plastocyanin reductase activity | MF | 7.72E-08 | 0.000147 | 7 | 8 | 2104 | 29073 | 6 |
| GO:0008569 | minus-end-directed microtubule motor activity | MF | 9.90E-06 | 0.01889 | 6 | 9 | 2104 | 29073 | 10 |

## Supplementary Table 21. KEGG pathway enrichment of unique gene families in the *C. ensifolium* genome.

| Map ID | Map Title | P value | Adjusted P value | x | y | n | N |
| --- | --- | --- | --- | --- | --- | --- | --- |
| map00730 | Thiamine metabolism | 5.64E-06 | 0.000496 | 8 | 16 | 2104 | 29073 |
| map00073 | Cutin, suberin and wax biosynthesis | 9.17E-06 | 0.000807 | 12 | 38 | 2104 | 29073 |
| map03018 | RNA degradation | 0.000105 | 0.00924 | 25 | 153 | 2104 | 29073 |

## Supplementary Table 22. Chloroplast genes of *C. ensifolium.*

| **Category for genes** | **Group of genes** | **Name of genes** |
| --- | --- | --- |
| Self-replication | Ribosomal RNAs | *rrn4.5*, *rrn5*, *rrn16*, *rrn23* |
|  | Transfer RNAs | *trnA^UGC^*, *trnC^GCA^*, *trnD^GUC^*, *trnE^UUC^*, *trnF^GAA^*, *trnfM^CAU^*, *trnG^GCC^*, *trnG^UCC^*, *trnH^GUG^*, *trnI^CAU^*, *trnI^GAU^*, *trnK^UUU^*, *trnL^CAA^*, *trnL^UAA^*, *trnL^UAG^*, *trnM^CAU^*, *trnN^GUU^*, *trnP^UGG^*, *trnQ^UUG^*, *trnR^ACG^*, *trnR^UCU^*, *trnS^GCU^*, *trnS^GGA^*, *trnS^UGA^*, *trnT^GGU^*, *trnT^UGU^*, *trnV^GAC^*, *trnV^UAC^*, *trnW^CCA^*, *trnY^GUA^* |
|  | Small Ribosomal protein | *rps2*, *rps3*, *rps4*, *rps7*, *rps8*, *rps11*, *rps12*, *rps14*, *rps15*, *rps16*, *rps18*, *rps19* |
|  | Large Ribosomal protein | *rpl2*, *rpl14*, *rpl16*, *rpl20*, *rpl22*, *rpl23*, *rpl32*, *rpl33*, *rpl36* |
|  | RNA polymerase | *rpoA*, *rpoB*, *rpoC1*, *rpoC2* |
| Genes for photosynthesis | Photosystem I | *psaA*, *psaB*, *psaC*, *psaI*, *psaJ* |
|  | Photosystem II | *psbA*, *psbB*, *psbC*, *psbD*, *psbF*, *psbG*, *psbH*, *psbI*, *psbJ*, *psbK*, *psbL*, *psbM*, *psbN*, *psbT*, *psbZ* |
|  | Cytochrome b/f complex | *petA*, *petB*, *petD*, *petG*, *petL*, *petN*, *psbE* |
|  | ATP synthase | *atpA*, *atpB*, *atpE*, *atpF*, *atpH*, *atpI* |
|  | ATP-dependent protease subunit p gene | *clpP* |
|  | RuBisCO large subunit | *rbcL* |
|  | NADH dehydrogenase | *ndhA^Ψ^*, *ndhB^Ψ^*, *ndhC*, *ndhD*, *ndhE*, *ndhG^Ψ^*, *ndhI*, *ndhJ*, *ndhK^Ψ^* (loss *ndhF* and *ndhH*) |
| Other genes | Maturase | *matK* |
|  | Envelop membrane protein | *cemA* |
|  | Subunit of acetyl-CoA-carboxylase | *accD* |
|  | c-type cytochrome synthesis ccsA gene | *ccsA* |
|  | Translation initiation factor IF-1 | *infA* |
| Genes of unknown function | Hypothetical chloroplast reading frames | *ycf1*, *ycf2*, *ycf3*, *ycf4* |

Note: gene*^Ψ^* means Pseudogene.

## Supplementary Table 23. Mitochondrial genes of *C. ensifolium*.

| **NO.** | **Gene type** | ***C. ensifolium*** |
| --- | --- | --- |
| 1 | ATP synthase protein | *atp1* |
| 2 |  | *atp4* |
| 3 |  | *atp6* |
| 4 |  | *atp8* |
| 5 |  | *atp9* |
| 6 | heme exporter protein | *ccmB* |
| 7 | cytochrome c biogenesis | *ccmC* |
| 8 | cytochrome c maturation | *ccmFc* |
| 9 |  | *ccmFn* |
| 10 | cytochrome b | *cob* |
| 11 | cytochrome c oxidase | *cox1* |
| 12 |  | *cox2* |
| 13 |  | *cox3* |
| 14 | Maturase | *matR* |
| 15 | Trimethylamine methyltransferase | *mttB* |
| 16 | NADH dehydrogenase | *nad1* |
| 17 |  | *nad2* |
| 18 |  | *nad3* |
| 19 |  | *nad4* |
| 20 |  | *nad4L* |
| 21 |  | *nad5* |
| 22 |  | *nad6* |
| 23 |  | *nad7* |
| 24 |  | *nad9* |
| 25 | ribosomal protein | *rpl16* |
| 26 |  | *rpl2* |
| 27 |  | *rpl23* |
| 28 |  | *rpl5* |
| 29 |  | *rps1* |
| 30 |  | *rps10* |
| 31 |  | *rps11* |
| 32 |  | *rps12* |
| 33 |  | *rps13* |
| 34 |  | *rps14* |
| 35 |  | *rps19* |
| 36 |  | *rps2* |
| 37 |  | *rps3* |
| 38 |  | *rps4* |
| 39 |  | *rps7* |
| 40 | Photosynthetic-related genes | *psaJ* |
| 41 |  | *psbK* |
| 42 |  | *petL* |
| 43 | ribosomal RNA | *rrn5* |
| 44 |  | *rrn18* |
| 45 |  | *rrn26* |
| 46 | transfer RNA | *trnC^GCA^* |
| 47 |  | *trnD^GTC^* |
| 48 |  | *trnE^TTC^* |
| 49 |  | *trnfM* |
| 50 |  | *trnfM^CAT^* |
| 51 |  | *trnH^GTG^* |
| 52 |  | *trnH^GTG^* |
| 53 |  | *trnI* |
| 54 |  | *trnI^AAT^* |
| 55 |  | *trnK^TTT^* |
| 56 |  | *trnM^CAT^* |
| 57 |  | *trnN^GTT^* |
| 58 |  | *trnQ^TTG^* |
| 59 |  | *trnR^ACG^* |
| 60 |  | *trnR^TCT^* |
| 61 |  | *trnS^GGA^* |
| 62 |  | *trnT^GGT^* |
| 63 |  | *trnV^TAC^* |
| 64 |  | *trnW^CCA^* |
| 65 |  | *trnY^AUA^* |
| 66 |  | *trnY^GTA^* |

## Supplementary Table 24. Analysis of the MADS-box genes of *C. ensifolium*.

| **Gene ID** | **Annotation** | **ORF (bp)** | **Protein length (aa)** | **Type** | **Subfamily** |
| --- | --- | --- | --- | --- | --- |
| JL008048 | *CeAP3-1* | 777 | 258 | Type II | B-AP3 |
| JL001850 | *CeAP3-2* | 684 | 227 | Type II | B-AP3 |
| JL012707 | *CeAP3-3* | 669 | 222 | Type II | B-AP3 |
| JL000566 | *CeAP3-4* | 678 | 225 | Type II | B-AP3 |
| JL018389 | *CeAG-1* | 705 | 234 | Type II | C |
| JL002233 | *CeAG-2* | 702 | 233 | Type II | C |
| JL015886 | *CeAG-3* | 669 | 222 | Type II | C |
| JL007940 | *CeSTK* | 675 | 224 | Type II | D |
| JL009493 | *CeAP1-1* | 705 | 234 | Type II | A |
| JL003631 | *CeAP1-2* | 744 | 247 | Type II | A |
| JL029086 | *CeAP1-3* | 726 | 241 | Type II | A |
| JL029087 | *CeAP1-4* | 726 | 241 | Type II | A |
| JL009494 | *CeSEP-2* | 738 | 245 | Type II | E |
| JL018852 | *CeSEP-4* | 744 | 247 | Type II | E |
| JL009870 | *CeSEP-1* | 612 | 203 | Type II | E |
| JL004042 | *CeSEP-3* | 660 | 219 | Type II | E |
| JL003970 | *CePI* | 558 | 185 | Type II | B-PI |
| JL022544 | *CeAGL6-1* | 729 | 242 | Type II | AGL6 |
| JL001354 | *CeAGL6-2* | 720 | 239 | Type II | AGL6 |
| JL013184 | *CeAGL6-3* | 684 | 227 | Type II | AGL6 |
| JL022543 | *CeSOC1-1* | 771 | 256 | Type II | SOC1 |
| JL006625 | *CeSOC1-2* | 669 | 222 | Type II | SOC1 |
| JL018953 | *CeSOC1-3* | 633 | 210 | Type II | SOC1 |
| JL011978 | *CeSVP-1* | 687 | 228 | Type II | SVP |
| JL011742 | *CeSVP-2* | 477 | 158 | Type II | SVP |
| JL019278 | *CeANR1* | 699 | 232 | Type II | ANR1 |
| JL015610 | *CeMIKC*-1* | 978 | 325 | Type II | MIKC* |
| JL027588 | *CeMIKC*-2* | 771 | 256 | Type II | MIKC* |
| JL024141 | *CeMIKC*-3* | 399 | 132 | Type II | MIKC* |
| JL012414 | *CeMIKC*-4* | 1053 | 350 | Type II | MIKC* |
| JL017169 | *CeMADS32* | 534 | 177 | Type II | CFO/MADS32 |
| JL025329 | *CeBs-1* | 609 | 202 | Type II | Bs |
| JL027956 | *CeBs-2* | 501 | 166 | Type II | Bs |
| JL026663 | *CeBs-3* | 654 | 217 | Type II | Bs |
| JL028086 | *CeBs-4* | 600 | 199 | Type II | Bs |
| JL025744 | *CeBs-5* | 606 | 201 | Type II | Bs |
| JL023912 | *CeBs-6* | 609 | 202 | Type II | Bs |
| JL026468 | *CeBs-7* | 636 | 211 | Type II | Bs |
| JL022727 |  | 657 | 218 | Type I | Mα |
| JL012642 |  | 690 | 229 | Type I | Mα |
| JL020276 |  | 474 | 157 | Type I | Mα |
| JL020277 |  | 537 | 178 | Type I | Mα |
| JL026477 |  | 615 | 204 | Type I | Mα |
| JL026478 |  | 615 | 204 | Type I | Mα |
| JL026171 |  | 723 | 240 | Type I | Mγ |
| JL028757 |  | 723 | 240 | Type I | Mγ |
| JL023734 |  | 678 | 225 | Type I | Mγ |
| JL011109 |  | 795 | 264 | Type I | Mγ |
| JL002443 |  | 777 | 258 | Type I | Mγ |
| JL012964 |  | 627 | 208 | Type I | Mγ |
| JL023877 |  | 534 | 177 | Type I |  |
| JL023880 |  | 534 | 177 | Type I |  |
| JL025738 |  | 411 | 136 | Type I |  |
| JL025905 |  | 549 | 182 | Type I |  |
| JL028221 |  | 621 | 206 | Type I |  |
| JL025739 |  | 456 | 151 | Type I |  |
| JL028222 |  | 438 | 145 | Type I |  |
| JL026588 |  | 645 | 214 | Type I |  |
| JL026732 |  | 645 | 214 | Type I |  |
| JL026760 |  | 1053 | 350 | Type I |  |
| JL026928 |  | 621 | 206 | Type I |  |
| JL027017 |  | 645 | 214 | Type I |  |
| JL028631 |  | 546 | 181 | Type I |  |
| JL028249 |  | 1029 | 342 | Type I |  |
| JL025560 |  | 645 | 214 | Type I |  |
| JL024818 |  | 642 | 213 | Type I |  |
| JL024437 |  | 768 | 255 | Type I |  |
| JL008456 |  | 996 | 331 | Type I |  |
| JL009973 |  | 546 | 181 | Type I |  |
| JL009975 |  | 603 | 200 | Type I |  |
| JL024820 |  | 813 | 270 | Type I |  |

## Supplementary Table 25. Volatile constituents of *C. ensifolium*’s flowers.

| **No.** | **Compound** | **Molecular formula ([Mol](javascript:;).**[**wt**](javascript:;)**.)** | **Relative amount (%)** | **Matched degree (%)** |
| --- | --- | --- | --- | --- |
| 1 | Methyl jasmonate | C_13_H_20_O_3_（224） | 12.1 | 91 |
| 2 | Acacia alcohol (sesquiterpenes) | C_15_H_26_O（222） | 1.23 | 78 |
| 3 | Linalool (monoterpenes) | C_10_H_18_O（154） | 1.01 | 85 |
| 4 | β-pinene (monoterpenes) | C_10_H_16_（136） | 0.24 | 66 |
| 5 | Basilene (monoterpenes) | C_10_H_16_（136） | 0.21 | 87 |
| 6 | α-Citral (monoterpenes) | C_10_H_16_O（152） | 0.11 | 56 |
| 7 | Bemulone (Sesquiterpenes) | C_15_H_24_O（220） | 0.07 | 57 |
| 8 | Longifolene (sesquiterpenes) | C_15_H_24_（204） | 0.04 | 38 |
| 9 | E, E-Acaciaol (sesquiterpenes) | C_15_H_24_O（220） | 0.01 | 76 |
| 10 | Geranylatone | C_13_H_22_O（194） | 0.002 | 75 |
| 11 | Isospinone (monoterpenoids) | C_10_H_16_O（152） | 0.001 | 49 |
| 12 | Nonanal (monoterpenes) | C_10_H_16_O（152） | 0.0001 | 27 |

## Supplementary Table 26. The enzymes related to fragrance regulatory networks in *C. ensifolium*.

| **Function** | **Abbreviated names** | **Enzyme** |
| --- | --- | --- |
| Jasmonate biosynthesis | PLD | phospholipase D |
|  | DAD1 | defective in anther dehiscence 1 |
|  | LOX | lipoxygenase |
|  | AOS | allene oxide synthase |
|  | AOC | allene oxide cyclase |
|  | OPR3 | 12-oxophytodienoate reductase 3 |
|  | JMT | jasmonic acid carboxyl methyltransferase |
| Terpene backbone biosynthesis | AACT | acetyl-CoA-C-acetyltransferase |
|  | HMGS | hydroxymethylglutaryl-CoA synthase |
|  | HMGR | hydroxymethylglutaryl-CoA reductase |
|  | MVK | mevalonate kinase |
|  | PMK | phosphomevalonate kinase |
|  | MVD | diphosphomevalonate decarboxylase |
|  | IDI1 | isopentenyl-diphosphate delta-isomerase 1 |
|  | IDI2 | isopentenyl-diphosphate delta-isomerase 2 |
|  | FDPS | farnesyl diphosphate synthase |
|  | DXS | 1-deoxy-D-xylulose-5-phosphate synthase |
|  | DXR | 1-deoxy-D-xylulose-5-phosphate reductoisomerase |
|  | MCT | 2-C-methyl-D-erythritol 4-phosphate cytidylyltransferase |
|  | CMK | 4-diphosphocytidyl-2-C-methyl-D-erythritol kinase |
|  | MDS | 2-C-methyl-D-erythritol 2 |
|  | HDS | 4-hydroxy-3-methylbut-2-enyl-diphosphate synthase |
|  | HDR | 4-hydroxy-3-methylbut-2-enyl diphosphate reductase |
|  | GDPS | geranyl diphosphate synthase |
|  | LIS | linalool synthase |

## Supplementary Table 27. The wild type and mutants of *C. ensifolium*.

| **No.** | **Materials** |
| --- | --- |
| 1 | The branched inflorescence with multitepal flowers mutant type (**Fig. 4b**) |
| 2 | The peloric flower mutant type (**Fig. 4c**) |
| 3 | The column-like petal mutant type (**Fig. 4d**) |
| 4 | The lip-like petal mutant type (**Fig. 4e**) |
| 5 | The lip-like sepal mutant type (**Fig. 4f**) |
| 6 | The yellow-green leaf mutant type (**Supplementary Fig. 23b**) |
| 7 | The yellow leaf mutant type (**Supplementary Fig. 23c**) |
| 8 | The white leaf mutant type (**Supplementary Fig. 25**) |
| 9 | The perianth-like leaf mutant type (**Supplementary Fig. 26**) |
| 10 | The wild type 1 (**Fig. 4a**) |
| 11 | The wild type 2 (**Supplementary Figs. 16–18**) |

# Supplementary Data

## Supplementary Data S1. MADS-box proteins of *C. ensifolium*.

>JL000566

MGRGKIEIKKIENPTNRQVTYSKRRAGIMKKAREITVLCDAQVSLVMFSSTGKFSEYCSPSTGMKKIFERYQQVSGINLWSAQSVKMQNTLNHLKEVNRNLRREVRQRMGEDLEGLDIKELRGLEQNIDEALKLVRNRKYHVISTQTDTYKKKLKNSEETHRSLMRELEIVEDHPAFGFDDESSNYEHGVLAIANEGPLTYAFRVHPSQQNLHGMGHRSHHLRLA*

>JL001354

MGRGRVELKRIENKINRQVTFSKRRNGIMKKAYELSVLCDAEIALIIFSSRGKLFEFGSPDITKTLERYQRCTFTPQTIHPNDHETLNWYQELSKLKAKYESLQRSQRHLLGEDLDMLSLKELQQLERQLESSLSQARQKRTQLMLDQMEELKKKERHLGDINKQLKHKLGADGGSMRALQGSWRPEAGTSNDTFRNHSINLDAEPTLQIGRYHQYVPSEATIPRNGGAGNGFISGWAV*

>JL001850

MGRGKIEIKKIENPTNRQVTYSKRRVGILKKAKELTVLCDAQVSLIMFSTTGKLADYCSPSTDLKGIYERYQIVTGMDLWNAQYERMQNTLNHLKEINQNLRKEIRQRNGEELEGMDIKELRGLEQTFEESIRIVRQRKYHVISTQTDTYKKKLKSTRETYRALIHELEMKDENPNYNFSEENHSRVYQNSIPMATECPQMFSFRVVQPIQPNLLGLGYESHDLSLA*

>JL002233

MEPKEKMGRGKIEIKRIENTTNRQVTFCKRRNGLLKKAYELSVLCDAEVALVIFSSRGRLYEYANNSVKGTIDRYKKACTDNSSTGSISEANSQYYQQEATKLRQQITNLQNSNRNLLGDALTTMSLRDLKQLETRLEKGISKIRSKKNELLHAEIDYMQKREMDLQTDNMYLRSKIADNERAQQHQHMSILPSTSTEYEVMPPFDSRSFLQVNLLDPSDHYSHQQQTALQLG*

>JL002443

MTRKKVTLAYITNDATRRATLKKRRRGLLKKVNELSILCGVPACAVVYSPQSDQPEVFPSAEEAKRILTELASLPEIDKNKRMVNQRTFLEQRLVKLSQQVRRLEHENKELVKAVCLRQCLTGRPVDTLSKEEAEDLLDLVDRKGKALQVRMHQLGPLMLPPVVEDMDVKEGYSFDGAAIEAMRQCDWISGCGGGRAGMMGVGGGGEEVMAGVGGSGGGGSLVAGGGGGGDDEMLKILIRQLSGNRSEWEEFPPETCF*

>JL003631

MGRGRVQLKRIENKINRQVTFSKRRSGLLKKAHEISVLCDAEVALIVFSNKGKLYEYSTEASMEKILERYERHSYAERALFSNEANSQADWRLEYNKLKARVESLQKSKRHLMGEQLDSLSTKELQHLEQQLESSLKHIRSRKTQLMLDSISELQKKEKLLLDQNKTLEKEIMAKEKAKALVQNAPWEKQNQCQYSSAPSHAEISNFGSTPASRTLRARASEEESPQPQLRLGNTLLPPWMLTRMNG*

>JL003970

MGRGKIEIKRIENSTNRQVTFSKRRNGIMKKAKEISVLCDAQVSLVIFSSLGKMFEYCSPSTTLSKILEKYQQNSGKKLWDAKHENLSAEIDRIKKENDNMQIELRHLKGEDLNSLNPKELIPIEETLQNGLTSVRNKQHHQQLAMEGSMRELDIGYHQKDREYAAQMPMTFRVQPIQPNLQGNK*

>JL004042

MGRGRVELKRIENKINRQVTFAKRRNGLLKKAYELSVLCDAEVALIIFSNRGKLYEFCSNNSMMKTIEKYQKSNYGAPETNVISRETQSSQQEYLKLKSRVEALQRSQRNLLGEDLGPLSSKELEQLERQLDSSLKQIRSTRTQFMLDQLADLQRREQMLCEANKTLKRRQLEESNQANPQQIWDPSTAHAMGYDRQPAQPHGDAFYHPLECEPTLQIG*

>JL006625

MVRGRTEMRRIENPTSRQVTFSKRRNGLLKKAFELSVLCDAEVGLIVFSPRGKLSEFASSSMLKTIERYRMNSKEMISNKKSTDEDIQQWKQDTDFMSKKIDSLEDSKRKLMGENLESCSVEELHELESRLEQSIRKVRGRKNHLLEEQVVQLKERERVLLEENALLQKQGRHATFSLWKEPVLCLNTSMEVVPQCDEYRDVETELYVGWPGRGRTQQLMKG*

>JL007940

MGRGKIEIKRIENTTNRQVTFCKRRNGLLKKAYELSILCDAEIALIVFSSRGRLYEYCSNSTKATIERYKKASANSSNSVVEINSQQYYQQEAAKLRHQIQILQNTNSHLAGEGVTSLSIKELKQLESRLERGLTRIRSKKHELLFAEMEFMQKREEDLQNENIFLRAKIAENERAQANIATNAASLDTLSTFDSRNYYHVNMLEAAANYHNQDQTALHLGYDN*

>JL008048

MRTSALLNCRTTLACLRKREREREREKKGELLLKEDKEVKAMGRGKIDIKKIVNPTNRQVTYSKRRLGIMKKAKELTVLCDAQVSLIMFSSSGKLADYCSPSTEIKDIFERYQQVTCIDFWDPQYQRMQNTLKNLREINHNLQKEIRQRKGENLEGLDVKALRGLEQKLEESIKLVRQRKYHVIATQTDTFKKKLRSTTEIYAALLHELKLEDDNQRSSFVAEDLSGVYDCAISMANQQHSEPVMQKVVYESHHLRFP*

>JL008456

MIFSFSGTRFLSFVYSFCFSSVSYFSPLLRCCKKEERRGEGRTAGDMGRAKLEMKYLENTRARRTTYMARMKGVKKKAGELSLLCGVDVLVASVSPELNTVEFWPKKDTPEFRRIRERYICFRKKKNDINCESNGFGDEGNDNSEQQIPCLPMHSPFLGLPSFHEEMIAMEIKLKEVRERLCFLGDCSLSENIIREHQEKEERLQAQFTSFMSSQEDLVHLHSIDQTSLFSQCLPLQFTDFNIPLQLDIHPYEKASPSVQSLISHQQPSPNAQTLPPYQQPSPFAHFLTSDLVDFNTMNPLVSFMPSDSSLWSHLDDLNWCNSLLMDYESG*

>JL009493

MGRGRVQLKRIESKINRQVTFSKRRSGLLKKAHEISVLCDAELALIIFSSRGKLYEYSTGTCMERILEQYERYRYAERALLINESDPQEDLRNEYGYLKSKVDALQRSRSNLLGEKLDTLSLEELQQLEQQLETALKRTRSQMNQHLLDCIAEHQRKEKSLLEQKIALKKKIIESENSIKMLQQMCTNKHCQAQISSSSPPTFQATNSVPTINIGTYLACSAGQDVFDEPLLFG*

>JL009494

MGRGRVELRRIENKINRQVTFAKRRNGLLKKAYELSVLCDAEVALIVFSNRGRLFEFCSSSSMAKTLERYQKCSYNASDSMVPSKDTQHSYQEYLKLKAKVEYLQLSQRNLLGEDLVELSSKELEELELQLEMSLKHIRSTKTQLMLDQLCDLKRKEQILHEANRALKRKLQDDGPEIPLELTWPGGGANGSCERHQPQPDELFFQPLPCDPSLQIGYSPIYIDQQLNTGSTSAHNINGFFSGWM*

>JL009870

MGRGRVELKRIENKINRQVTFAKRRNGLLKKAYELSILCEAEVALIVFSNRGKLYEFCSSTRNLLGEDLGPLSSKELEQLERQLDASLKQIRSTRTQFMLDQLADLQRREQMLCEANKALKRRFVETSQTNPNLVWDTSNTHAVGYGRQHAQHHEDAFYHPLESEPTLQIGYHSDITMAPGTATSVSNFMPSGWLGQISGSYE*

>JL009973

MGRAKLEVKYKEDTRGRIHTYKTRINGIEKKAKELSVLCGVDVLFASVSPDFNALHYWPNNPSDFHRILDRYKSSSSNPPCLVSPLPLNSSFQEELVDFNKKLEEVRQRICFLEAEKMASLSQQISEEISMEEKEDCCPSAVAVNDDDIYSTLEDLDEDLILKDDCNIACSENYFVPTNVC*

>JL009975

MGRAKLEVKYREDARVRINTYRTRMKGVEKKAKELSVLCGVDVLLASFSPDLNALQYWPNNPSDFRRIVDRYKSISSNPPFLIPPLPLNSSYQEELVDFNKKLEEVRQRICFLEAEKMTASSQQISEERSMEEKEDCYPSAVAVNDHDDIYSTLEDLGEDLILKDDWNVACYENYFVPTNTCADFEGFFYVSVSVDRLVL*

>JL011109

MARKKVKLAWICNDTTRRATLKKRRKGLLKKVKELSILCDVRTCAIIYSPNEHQPEVWPSAPEATRLLSCFRAMPEMEQCKKMMNQETFLRQRVAKLHVKLRRHDRENHELESAALLGECMAGASLNNLRIEELVVLAWILDQKTRAVQDRIDHLRLRAALERPVAGSVSNVAVECMAVIPTPQPPRSQLPMLIPTSIFRDNKLGPYCPEMRWFLNGDEGGAATGGDHSYPQMDQLGCAGEGSCGVAAAFVEQNPDDWENFFPY*

>JL011742

MAREKIKIRKIDNATARQVTFSKRRKGLFKKAQELAILCDADVGLIIFSSTGKLFQFSSSSMKEIIEKHSLQSKSISRGDQPSLVLQLDNSDDGARLCKQASELTLQLRRLRGEDIDKLDLEELQKIESTLESGLSRVLEKKEQKIVEQINDLQQKVE*

>JL011978

MAREKIKIRKIDNATARQVTFSKRRRGLFKKAEELAILCDADVGLIVFSATAKLFDFSTSSMKEIIDRYNMHSKNLTAGDLPSLDLQLENGNLTILRKQVTEATRQLRNMRGEDLNGLTIQELQQLEKTLETGLSRVMQKKSQLCSSFSYLVGTQGIELMEENTRLRYQVAELAKIGKQAVVDPENALHEDGQSSESVTNVTHCGNPFENDDSSDTCLKLGLSCSGWK*

>JL012414

MGRVKLKIKRLENTSGRQVTYSKRRAGILKKAKELSILCDIDILLLMFSPTGKPTLCVGENSNVEEVITKFAQLTPQERTKRKLESLEALKKTFKKLDHDVNIPDFLGSSSHTVEELSNHLHNLQSQLLDMQKKLSFWMDPENVNTVEQITQMEDALREALNRIQMQKKCFGNELMPLDCVGEFHNGMHLPMNMTTNEQLPSNIQWIQDGDDRTFMFSGIPGFLPQRNYMYSTETSLQDYHGYFIDEKQAVCNKHGQDESLNDPSQNSCLQLQLGAQHPYNSYGMNFLGEKKFQTNGEVSLQADHVDFQVNGFDPARHGFDAELQTWATTGSCGVSMFDGHPYTQQPKQT*

>JL012642

MVKKKRQSMGRQKIAMKRIENEEARQVCFSKRRAGLFKKATELSILCGAEIGIVVFSPAGKPFSFGHPSLDYITRRFLGGGGVAVGQLCGIFVEGEKLHHLNQEHAGLMEKLEEARGRKAELEAQGAAQRVEPGLPCDMEVHEMSLEDLESFQKALEELRRNVANRREELELHCMDTKAIRGVPQRLGYGTGYLGGYNGLYLTSGPVGGFYGHGQDMSVGFRFGDRWLG*

>JL012707

MGRGKIEIKKIENPTNRQVTYSKRRAGIMKKASELTVLCDAQLSLVMFSSTGKFSEYCSPTTDTKSIYDRYQQVSGINLWSSQYEKMQNTLNHLKEINQTLRREIRQRMGEDLDGLEIKELRGLEQNMDESLKLVRNRKYHVISTQTDTYKKKLKNSQETHRNLLRELEAEHAVYYVDDDPNSYDGALALGNGPSYLYSFRSQPSQPNLQGMGYGPHDLRLA*

>JL012964

MARKKTRLAYITNDAARRITLRKRRLGVLKKVYELSVLCNVPACAIIYSPQETEPEVWPSAAEARNLLSRFNAMDSFSKNNNMMNPEKFLLKQIVKLRGQLGRLMAENDDLAGGVLLLECFAGRLVSDFESYELGLLGSALARRIEAVQDRLLMLRNSGAESQPLPLLLPPPAMEETAAGGVDSRTDGVLGWEQGLLTGCEDFFQPNV*

>JL013184

MGRGRVELKLIENKINRQVTFSKRRNGLMKKAFELSVLCDAEVALIIFSSRGKLFEFGSPDIKKTIGRYLRCTFSPQAMDPSDIEAQSWYIELSKLKAKYESLQQSQRHLLGEDLDDLSLKDLEQLEQQLESALSLGRQRRTEIMLNQMEELKKKLAAEYRPRSIHRSWGPNVVLTTRAFPMHPTHPNIMDPEPILQIGYHHYESSEATTSQRNGGAQSNFTQGWTL*

>JL015610

MGRVKLQIKRIENNTNRQVTFSKRRNGLIKKAYELSVLCDIDIALIMFSPSGRLSHFSGRRRIEDVIGRYVNLPEHDRAGVNMNKEYLMRSLNKLKCESDMTVQLSNDGVANTKAEKLQQEISMCQQQLQICVERLRYFDAAPMSISPCTPSTDLESCEKLFMDSLALVCDKKKLLLSNSEHPLPAYDSTAEFSVYLQPQQGGMPNGFGYDIVQWVPEIISNHGHPMFQNSDPFMSFRDDGIYNSITAQMEPQVGGDTWQQAYASAELLSSLIPPASLPLEQSPLATVEPPTVAGSTRYPHVPVEVEGMCDGGGIVDSSVRENVG*

>JL015886

MERNMKGRGKIEIKKIENATNRQVTFCKRRNGLLKKAYELAVLCDAEVALVIFSSRGRLYEYANNSVKGTIERYKKASTDNPNAGPTPQTNSQYYQQEASKLLQQINNLQNSNRNLMGEALSTMNLRDLKQLETRLEKGINKIRSKKNELLYAEIEYMQKKEIELQNDNMYLRNKIADNERVRQQQMNMLPSTRNQFEVMPPFDSRSFPQVNLLTGNSLPSN*

>JL017169

MMGRGRLEIKRIENPSQRQSTFYKRRDGLFKKARELAVLCDADLLLLLFSSSGKLYHYHSPSVSNVQELLRRYEVASRTRILKDQEGGKKAEEVERMCEILEREIRFMRIDDGEEYTLPVLQAIEQNLEVAVQKVREEKDRKIQAEMETLHRMVCSVLIFELSFCLMDLILKRVSSL*

>JL018389

MMEPKEKMGRGKIEIKRIENTTNRQVTFCKRRNGLLKKAYELSVLCDAEVALVIFSTRGRLYEYANNSVKATIEKYKKACSDNSNSGTISETNAQYYMQEASKLRQQITNLQNSNRNLMGEALSTMSLRDLKQLETRLEKGINKIRSKKNELLYAEIEYMQKREMELQNDNMYLRNKIAENERTQQQPHINMVPSTSTEYEVMPPFDSRNFLQVNLMDPSHHYSLQQQTALQLG*

>JL018852

MGRGRVELKMIENKINRQVTFAKRRNGLLKKAYELSVLCDAEVALIIFSNRGRLFEFCSSSSMMKTLERYQKCSYNSSETTIPSKETQNSYQEYLKLKARVEYLQRSQRNLLGEDLGQLTTKELEQLEHQLEMSLKQIRSTKSQLMLDQLCDLKRKEQMLQEANRALRMKLQEDEPEIPLQLSWPGGGGSGRNGRGHCESHPQSEVFFQPLPCDPSLQIGYNPVCLEQQLNTGSSSHSVNGFIPGWM*

>JL018953

MVRGKKEMRRIENAASRQVTFSKRRKGLLKKAFELSVLCDAEIALTVFSSSGKLYEFSSSSMLNTIERYKAFSKDMVDNGRSTEQNSQEWKQDVEAMARKIELLDISKRKLMGESLESCSVEELQELERQLQQSLSSIKKLKNHVLKEQITQLKDRERQLQEENDLLQKQANHLASSLVVLQGENNYKEVETELYIGWPGSARTQQLLKG*

>JL019278

MGRGKIEIRRIDNITNRQVTFSKRRNGLLKKAKELAVLCDAEIGLIIFSSTGRLYDFASSSMKSVIARYNKAKRDHQQAMHANSEHKIWQRKATSLSQQLHNLQENHRQLMGEDIDGLSIKELQNLENQIETSLHAIQQKKDQVLINEVQELNQKIVLMHQENKELYKMLNLCRQENMDLNKKVNATKGLTGGSTSTNSGSPASIYLELSPPQQKISGMHADFQTLGRLQLH*

>JL020276

MVGRGRQKTVLKPIEKQESRLVCFSKRRSGLFKKASELSILTGDKVGAIVFSPAGKLFALETPTIDSLLGCQMEEGANSSTESESVDAMVWKEAPFWWDKPQVQAMSFEELLQFQRKMLDYGEKKFGNCAERLLQGNDSTERTLLIQVSAGRTQLSQ*

>JL020277

MVGRGRQKTVLKPIEKQESRLVCFSKRRSGLFKKASELSILTGDKVGAIVFSPAGKLFALETPTIDSLMGCQMEEGANSSTESESVDAMVWKEAPFWWDKPQVQAMSFEELLQFQRKMLDYGEKKFGNCAERLLQGNDSTERTLLIQVSAGRTQLASQQLHADRNRDVPLRIRLKFGF*

>JL022543

MDWGKTEIRRIENSTSRQVTFSKRRNGLLKKAVELSVLCDVKLGIIVFSPQGKVYELASSRGIASPSILDPAASLEEIILGDLISAVCSVRRMQNVIERYKMHLVDLSSNSSATEQNIEMISFALHHVALIRHWKQEAALSAKKIEFLEASKRMLMGENLESCSLEDLNKLEGQLEEGIGNIRGVKARLLSGRVTQLKEKTGVLSEENELLQNQKCMETQLILDSLVALQDNNDQPKDMDIETDLHIGCPGRGKKF*

>JL022544

MGRGRVELKRIENKINRQVTFSKRRNGLLKKAYELSVLCDAEVALIIFSSRGKLYEFGSAGTCKTLERYQRSCLNSQATNSIDRETQSWYQEVSKLKSKFESLQRSHRNLLGEDLGPLNVKELQQLERQLETALSQARQRKTQIMLDQMEELRKKERQLGEINKQLKMKLEAGGGSLRLMQGSWESDAVVEGNAFQMHPYQSSSLECEPTLHIGYHHFVPPETVIPRTPGVENNNFMLGWML*

>JL022727

MDPIEPKKKSKGRKKIEIKRIEKEESRQVCFSKRRQGLFKKANELCTLCGAEVAIVAFSLAGKPYSFGHPSVDHVLRRFLDAPAPFDTFTSATNCSEPMSQELAYLRESLEGEMRRRGALEEALKVPWPVPKPFWWDSDVSSMGIEELKDYGKLLLEYQDMVAQAAQQSCFQVPEAAMGVIDSLPALPAPAGAGEADYFRWLQQQDVELAGSGFNFEA*

>JL023734

MARKKVKLEWIANDSARRATFKKRRKGLVKEVRSACLIVYAPRETQPEVWPSPPEAKRVLAKLRRLPEMEQSKKMNQECDDSSHLYVEKEEERLRRKFESEGKELLPKQEYYEVSDSNVITPGTIFVQKLSKALEYHIRLKLNNDSSWKSIMLIELKLKEVKWRLENLSREMAAKEEVKKEELPPMEEIMKETASPILDGKEKTTFDEAMEVLQKQDWHCKILNP*

>JL023877

MGRAKLEIKYRDESRARMNTYKTRIKGIEKKAEELSVLCGVDVLFSSFSPDLNTFHYWPRNPYEFHRIVDRYKSVSRNQRSVVPSQAQNSASQEELLDFNKKLEEVRQRICFLEAEKVKEDEDIYSMLEDLDEDLTLSDIPSISCAKIFNDDWNICTENYFDSTVMCSDLEGLSQIF*

>JL023880

MGRAKLEIKYRDESRARMNTYKTRIKGIEKKAEELSVLCGVDVLFSSFSPDLNTFHYWPRNPYEFHRIVDRYKSVSRNQRSVVPSQAQNSASQEELLDFNKKLEEVRQRICFLEAEKIKEDEDIYSMLEDLDEDLTLSDIPSISCAKIFNDDWNICTENYFDSTVMCSDLEGLSQIF*

>JL023912

MGRGKIEIRRIENSANRQVTFSKRRGGLLKKANELAILTDALIGIIIFSASGKLYKYNSPSQQLHCEMMKVRNETDMLQASIKQLTGEDLTGLIINDLNQLEEQLEFSVAKVCICMHELLHQQLENLRRQVRKTFSYIMSEQQLMIDDHKLWEKGLLEPIGQYLDDSRSLLQLGTQIHQFHLQPAQLNLQDAELHGPGLQLW*

>JL024141

MGRVKLQIRRIENNTNRQVTLSKRRNGLIKKTYELSVLCDIDIALIKFSPSGRLCQVSGRRRIEDVIGRYVNLPEHDRAGFLNAYLQYLMRTLNKLQCESDMAIQLSKSSTYLETCEKIFYGCYGRPDNLYL*

>JL024437

MPPASQVRFFSLPYRFLVFSFLFQGGREGTERAMGRAKLEMKYRENTRARRATYMTRMQGLKKKAAELSILCGVDVLIASLSPEVNAVQFWPEQDAVEFRRIREVYIHFRRSRKSDGCSDEENNPFQPMPSPSFPSIPEEMADMNIKLREVRERLCFLEAASSDAANEEQTVLQPLQVVISSQLEEKQTLQVQGMFPSSLPSLDQPSPSGQALISHSDFTLEGPPIFLALPSNSALWRFMDDCNWCDSLVDYDKW*

>JL024818

MGRAKLEIKYRENLRARHSTYMARMKGVKKKASELSLLCGVDVLVASLSPELNTVEFWPEKDTPEFHRIRERYLHFRKKKNNCSGDEGNENNEQQTPCLSVPFPSFSLSIFHEEMVAMEMKLKEVRERIYFFQASSQNQNHVDQCQVEEQLQAQSSIFRPFHHDLLRPVEQPFLYTQFLPHYLIDFNMPWQQDLLHPLEQPSPFVQNSPLHLP*

>JL024820

MGCAKLEMKYLENTRARRTTYMARMKGVKKKAGELSLLCGVDVLVASVSPELNTVEFWPKKDTPEFRRIRERYICFRKKKNDINCESNGFGDEGNDNSEQQIPCLPMHSPFLGLPSFHEEMIAIACSHSENIIREHQEKEERLQAQFTNFIPSQEDLVHLHSIDQTSLFSQYLPLQFTDFNIPLQLDIHPYEKASPSVQSLISHQQPSPYAQSLPPYQQPSPFAHFLTSDLVDFNTMNPLVSFMPSDSSLWSHLDDLNWCNSLLIDYESG*

>JL025329

MGRGKIEIKRLENTANRQVTFSKRRGGLLKKANELAILTDAQIGLIIFSASGKLYQYNSPSMQLHLEMMKVRNEADMLQASIKQLTGEDLTGLTINDLNQLEEQLEFLVAKVRTRMHQLLHQQLGNLRQHVRKTFSYIMSEQQLMIEDHKLREMGLLEPIGQYLDDSRSLLQLGTQIHQFHLQPAHLNLQDVELHGPGLHLW*

>JL025560

MGRAKLEMKYRDESRARLNTYKTRIKGMEKKAEELSVLCGVDVLFSSFSPDLNTFHYWPRNPSEFHRIVDRYKSVSRNQRSVVPSRGLNSAPEEELLDFNKKLGEVRQRICFLEAKKSRASSSSTSQQSCQVINFMEDKENDCASTVVLNEEKEEEDVYSRLEDLGENLILSDIPSISCSKNFNDDWNICTGNYFDPAVMFSDLEGLSQIYSYR*

>JL025738

MGRAKLEIKYRENLRARHSTYMARMKGVKKKASELSLLCGVDVLVASLSPELNTVEFWPEKDTPEFHRIRERYLHFRKKKNNCSGDEGAFSLSIFHEEMVAMEMKLKEVRERIYFFQASSQNENHVDQCQVEEEQL*

>JL025739

MGRAKLEIKYRENLRTRHSTYMARMKGVKKKASELSLLCGVDVLVVSLSPELNTVEFWPEKHTLEFHRIRERYLHFRKKKNNCSGDEGNENNEQQTPCLSVPFPSFSLSIFHEEMVAMEMKLKEVREIIYFFQAYSQNENHVDQCQVEEEQ*

>JL025744

MGRRKIEIKRLENTANRRVTFSKRRGGLLKKANELSILTDAQNGLIIFSASGKLYQYNSPSMSMEEIIEEYMKAKNIDIEDFSSHQFLLNSQLHCEMMKVRNETDMLQASIKQLTGEDLTGLTINDLNQLEEQLEFSVSKVRTLKIMSEQQKVMEQQLMIEDNKLRKMGLLKPIGQYLDDSRSLLQLGTLYPSISFATCPS*

>JL025905

MGRAKLEIKYRDESRARMNTYKTRIKGIEKKAEELSVLCGVDVLFSSFSPDLNTFHYWPRNPYEFHRIVDRYKSVSRNQRSVVPSQAQNSASQEELLDFNKKLEEVRQRICFLEAEKRWGSSIKEDQDIYSMLEDLDEDLTLSDIPSISCAKIFNDDWNICTENYFDSTVMCSDFEDLSQIF*

>JL026171

MARKKVKLEWIANDSARRATFKKRRKGLVKKVEELSTLCDVKACLIVYAPSESQPEVWPSPPEANRVLAKLRRLPEMEQSKKMMNQEGFMRHRIAKLQEQIRKQERENRELETLLLMHQGLMGKINLQNTESRSLTSLAWLIELKLKEVKRRLENLSREMAAKEEVKKEELPPMAEIMKETAAPTLEGKEKTAFDEAMEVLQKQDWYCEILNPQEEIMATVPPFFEHASPQWLDAYFPKN*

>JL026468

MGRGKIEIKRLENTANRQVTFSKRRGGLLKKANELAILTDAQIGLIIFSASGKLYQYNSPNSQQLHCEMMKVRNETDMLQASIKQLTGEDLTGLTINDLNQLEEQLEFSVAKVRTRMHQLLHQQLENLRRQEHKLEDQNNYLYRAVSSEQVKQLMIDDHKLTEKGLLEPIGQYLDDSRSLLQLGTQIHQFHLQPAQLNLQDAELHGPGLQL*

>JL026477

MAGKGRKRVENKLIDNPSSRMVCFSKRRKGLFKKAEELSILSGAKVTALTFSQAGKAYCSDFQTLDSLFCPQVSISSEEDGRESAFAEATAADSLVCPQLGMFAEKEAEKDTISSEKEVYMDENFYCSHVATFNPLFCPQFNVFVENEAENCWEFDFSEAMAGKELHFWYNKSNVQAMGCEELLRLERAILEYRQMQLTKNCYR*

>JL026478

MAGKGRKRVENKLIDNPSSRMVCFSKRRKGLFKKAEELSILSGAKVTALTFSQAGKAYCSDFQTLDSLFCPQVSISSEEDGRESAFAEATAADSLVCPQLGMFAEKEAEKDTISSEKEVYMDENFYCSHVATFNPLFCPQFNVFVENEAENCWEFDFSEAMAGKELHFWYNKSNVQAMGCEELLRLERAILEYRQMQLTKNCYR*

>JL026588

MGRAKLEMKYRDESRARMNTYKTRIKGMEKKAEELSVLCGVDVLFSSFSPDLNTFHYWPRNPSEFHRIVDRYKSVSRNQRSVVPSRGLNSAPQEELLDFNKKLEEVRQRICFLEAKKSRASSSSTSQQSCQVINFMEDKENDCASIVVLNEEKEDEDVYSRLEDLGEDLILSDIPSISCSKNFNEDWNIYTENYFDSAVMFSDLEGLSQIYSYR*

>JL026663

MGRGKIEIKRIENTANRQVTFSKRRGGLLKKANELAILTDAQIGLIIFSASGKLYQYNSPSMSMEEIIEEYMKAKNIDFEDFSSHQFLLNSQQLHCEMMKVRNETDMLQASIKQLTGEDLTGLTINDLNKLEEQLEFSIAKVRTRMIMFEQQKVMEQQLMIEDHKLREMGLLEPIGQYLDDSRSLLQLGTQIHQFHLQPAHLNLQDAELHGPGLHLW*

>JL026732

MGRAKLEMKYRDESRARLNTYKTRIKGMEKKAEELSVLCGVDVLFSSFSPDLNTFHYWPRNPSEFHRIVDRYKSVSRNQRSVVPSRGLNSAPEEELLDFNKKLEEVRQRICFLEAKKSRASSSSTSQQSCQVINFMEDKENDCASIVVLNEEKEEEDVYSRLDDLGEDLILSDIPSISCSKNFNDDWNIYTGNYFDPAVMFSDLEGLSQIYSYR*

>JL026760

MKPRSSAIIEAQRPYLVSIKREDDLLILRYEVFEFRLFLLLLFSVSYFSPLLRCCKKEERRGEGRTAGDMGRAKLEMKYLENTRARRTTYMARMKGVKKKAGELSLLCGVDVLVASVSPELNTVEFWPKKDTPEFRRIRERYICFRRKKNDINCESNGFGDEGNDNSEQQIPCLPMHSPFLGLPSFHEEMIAMEIKLKEVRERLCFLGACSHSENIIREHQEKEERLQAQFTNFIPSQEDLVHLHSIDQTSLFSQYLPLQFTDFNIPLQLDIHPYEKASPSVQSLVSHQQPSPYAQSLPPYQQPSPFAHFLTSDLVDFNTMNPLVSFMPSDSSLWSHLDDLNWCNSLLMD*

>JL026928

MGRTKLEIKYRENLRARHSTYMARMKGVKKKASELSLLCGVDVLVASLSPELNTVEFWPEKDTPEFRRIRERYLHFRNKKNNCSGDEGNENNEQQTPCLSVPFPSFSLSIFHEEMVAMEMKLKEVRERIYFFQACSQNENHVDQCQVEEEQLQAQSSIFRPFHHDLLHPVEQPFLYTQFLPHYLIDFNMSLQQDLLHPLEQPSPFV*

>JL027017

MGRAKLEMKYRDESRARMNTYKIRIKGMEKKAEELSVLCGVDVLFSSFSPDLNTFHYWPRNPSEFHRIVDRYKSVSRNQRSVVPSRGLNSAPEEELLDFNKKLEEVRQRICFLEAKKSRASSSSTSQQSCQVINFMEDKENDCASIVVLNEEKEEEDVYSRLDDLGEDLILSDIPSISCSKNFNDDWNICTGNYFDPAVMFSDLEGLSQIYSYR*

>JL027588

MGRVKLQIRRIENNTNRQVTFSKRRNGLIKKAYELSVLCDIDIALIMFSPSGRLSHFSGRRRIEDVIGRYVNLPEHDRAGVNMHKEYLMRTLNKLQCESDMAIQLSNAGMANTKAEKLQQEISMCQQQLQICVERLRYFDAAPMGISPCTTSTDLESCEKFFMDSLARVCDKKKLLLSNSQHPLPAYDPSTAEFSVYLQPQQGGMPNGFGYDVVQWVPEIISNHGHPMFQNSDPFMSFRYNISLAVFLKTLEFLTV*

>JL027956

MGRGKIDIKRVENTANRKVTFSKRRGGLLKKANELAILIDAQISLIIFSASGKLYQYNSPQLHCEMMKVRNETDMLQASIKQLTGEDLTGLTINDLNKLEEQLEFSVAKVRDHKLREMGLLEPIGQYLDDSRSLLQLGTQINQFHLQPAHLNLQDAELHGPGLHLW*

>JL028086

MGRGKIEIKRLENTANRQMTFSKRRGGLLKKANELAILIDAQIGLIIFSTNGELYQYNNPSMSVEEIVEEYMKAKNIDIEDFSSHHQLHCEMMKVRNETDMLQASIKQLTGEDLTGLTINDLNQLEEQLEFSVAKIMSEKQLMIDDHKLWEKGLLEPIGPYLEDSRSLLQLGTQIHQFHLQPAQLNLQDAELHGPGLQL*

>JL028221

MGRAKLEIKYRENLHACHSTYMARMKGVKKKASELSLLCGVDVLVASLSRELNTVEFWPEKDTLEFQRIRERYLHFRKKKNNYSGDEGNENNEQQTPCLSVPFPSFSLSILHEEIVATEMKLKEVRESIYFFQASSQNENHVDQCQVEEEQLQAQSSIFRPFHHDLLHPVEQPFLYTQFLPHYIIDFNMPLQQDDLHPLEQPSPFV*

>JL028222

MGRAKLEIKYRENLRARHSTYMMRMKGVKKKASELSLLCGVDVLVASLSPELNTVEFWLEKDTPEFHQIRERYLHFCKKNNNCSADEGNENNEQQTPCLSVPFPSFSLSIFHEEMVAMEMKLKEVRERIYFFQASSQNENYVDQC*

>JL028249

MKPRSSAIIEAQRPYLVSIKREDDLLILRYEVFDFRLFLLLLFSVSYFSPLLRCCKKEERRGEGRTAGDMGRAKLEMKYLENTRARRTTYMARMKGVKKKAGELSLLCGVDVLVASVSPELNTVEFWPKKDTPEFHRIRERYICFRKKKNDINCESNGFGDEGNDNSEQQIPCLPMHSPFLGLPSFHEEMIAMEIKLKEVRERLCFLGACSLSENIIREHQEKEERLQAQFTNFMSSQEDLVHLHSIDQTSLFSQCLPLQFTDFNIPLQLDIHPYEKASPSVQSLISHQQPSPNAQTLPPYQQPSPFAHFLTSDLVDFNTMNPLVSFMPSDSSLWSHLDDLN*

>JL028631

MGRAKLEVKYKEDTRGRIHTYKTRINGIEKKAKELSVLCGVDVLFASVSPDFNALHYWPNNPSDFHRILDRYKSSSSNPPCLVSPLPLNSSFQEELVDFNKKLEEVRQRICFLEAEKMASLSQQISEEISMEEKEDCCPSAVAVNDDDIYSTLEDLDEDLILKDDCNIACSENYFVPTNVC*

>JL028757

MARKKVKLEWIANDSARRATFKNRRKALVKKVEELSTLCDVKACLIVYAPSESQPEVWPSPPEANRVLAKLRRLPEMEQSKKMMNQEGFMRHRIAKLQEQIRKQERENRELETLQLMHQGLMGKINLQNTESRSLTSLAWLIELKLREVKRRLENLAREMAAKEEVKKEELPPMAEIMNETAAPMLEGKEKTAFDEAMEVLQKQDWYCEILNPQKEIMATVPPFFEHASRQWLDTYFPKK*

>JL029086

MGRGRVQLKRIENKINRQVTFSKRRSGLLKKAHELSILCDAEVALIIFSTKGKLYEYSTDSCMDRILERYERYCYAEKALQVTEPESQGDKCHEYGKLKNKIEALQKSRSHLMGEKLDTLSLKELQHLDQQLETALKHIRSQRTQLLLNSIAELQRKEKSLLEHNSLLEKKITENELATKWKQQRQQDQESSSPPPFLPTNDLPTLNLGTYPVSNGQEVAEPTLTRMNSNGLPPWMLRSST*

>JL029087

MGRGRVQLKRIENKIKRQVTFSKRRSGLLKKANEISVLCDADVALIIFSNKGKLYEYSTDSCMEKILERYESFSYAEKAILCNEDDPQADWCLEYSKLKARVESLQRSQRHLIGEELDTLSNKELQHLEQQLENSLKHIRSKKTDLMLDSIAKLQNKEKMLREQKKNMEEIIATEKAKAFAQNESVDQQKQLPHNSSPHRILISDSVPTPISRSNEEEASQAELREGIGLLPSWMFNHLKG*

# Supplementary References

1. Chao, Y. T. *et al*. Chromosome-level assembly, genetic and physical mapping of *Phalaenopsis aphrodite* genome provides new insights into species adaptation and resources for orchid breeding. *Plant Biotechnol. J.* **16**, 2027–2041 (2018).
